# Ultraviolet photodissociation and collision-induced dissociation for qualitative/quantitative analysis of low molecular weight compounds by liquid chromatography-mass spectrometry

**DOI:** 10.1007/s00216-023-04977-0

**Published:** 2023-10-07

**Authors:** Romain Giraud, Yves J. C. Le Blanc, Mircea Guna, Gérard Hopfgartner

**Affiliations:** 1 Life Sciences Mass Spectrometry, Department of Inorganic and Analytical Chemistry, University of Geneva, 24 Quai Ernest Ansermet, CH-1205 Geneva 4, Switzerland; 2 SCIEX, Concord, ON L4K 4 V8 Canada

**Keywords:** LC–MS/MS, Ultraviolet photodissociation, Collision-induced dissociation, Qualitative analysis, Quantitative analysis, Low molecular weight compounds

## Abstract

Collision-induced dissociation (CID) is the most wildly used fragmentation technique for qualitative and quantitative determination of low molecular weight compounds (LMWC). Ultraviolet photodissociation (UVPD) has been mainly investigated for the analysis of peptides and lipids while only in a limited way for LMWC. A triple quadrupole linear ion trap instrument has been modified to allow ultraviolet photodissociation (UVPD) in the end of the q2 region enabling various workflows with and without data-dependent acquisition (DDA) combining CID and UVPD in the same LC–MS analysis. The performance of UVPD, with a 266-nm laser, is compared to CID for a mix of 90 molecules from different classes of LMWC including peptides, pesticides, pharmaceuticals, metabolites, and drugs of abuse. These two activation methods offer complementary fragments as well as common fragments with similar sensitivities for most analytes investigated. The versatility of UVPD and CID is also demonstrated for quantitative analysis in human plasma of bosentan and its desmethyl metabolite, used as model analytes. Different background signals are observed for both fragmentation methods as well as unique fragments which opens the possibility of developing a selective quantitative assay with improved sample throughput, in particular for analytes present in different matrices.

**Graphical Abstract:**

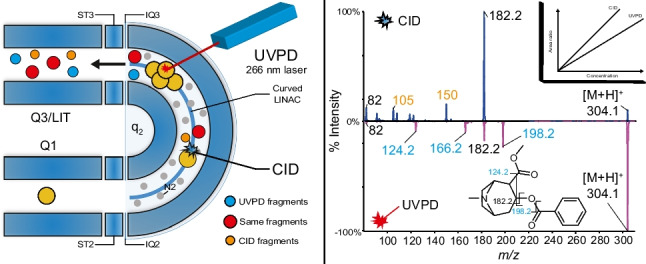

**Supplementary Information:**

The online version contains supplementary material available at 10.1007/s00216-023-04977-0.

## Introduction

Liquid chromatography coupled to tandem mass spectrometry is largely used for analysis of low molecular weight compounds (LMWC) in pharmaceuticals, environmental, food, medical sciences, biology and forensic using triple quadrupole mass analyser, quadrupole time of flight or Orbitrap systems. Generation of product ions used for qualitative and quantitative analysis is generally achieved by collision of selected accelerated precursors ions with a neutral gas such argon and nitrogen (ion/neutral) and referred as collision-induced dissociation (CID) [1]. CID fragmentation is non-specific and strongly dependent on the analyte and the collision energy. A limited number of product ion fragments jeopardize compound identification or affect the limit of quantification in case of interferences and calls for alternative dissociation methods. Various strategies have been developed over the years based on different types of interactions such as electron-based methods (ion/electron) or photon activated based methods such as UV photodissociation (ion/photon) [2]. The main objectives are to enhance selectivity and/or to obtain complementary information. UV photodissociation is largely applied for the analysis of biological molecules of interest such as proteins, peptides, nucleic acid, and lipids [3, 4] but far less for the analysis of singly charged LMWC where CID remains the most popular method. Ultraviolet photodissociation mass spectrometry appeared in the mid-1980s on Fourier transform mass spectrometers (FTMS) using 193-nm laser to sequence peptides [5, 6] where similar spectra with higher efficiency compared to CID were generated. 2D and 3D ion traps are particularly suitable for UVPD, firstly because of ease of optical access and secondly as the selected ions can be trapped enabling a good overlap with the laser beam [7]. Hybrid triple quadrupole linear ion trap mass spectrometers are well suited for CID as well as for UVPD [8] offering the possibility to acquire in the selected reaction monitoring mode (photo-SRM) [9]. Time of flight (TOF) offer high resolution and accurate mass for UVPD but its implementation on TOF or TOF-TOF [10] has often been described to be challenging because of the need to match fast acquisition and photodissociation efficiencies. Lai et al. [8] reported that pressure is an important factor to generate unique UVPD fragments (266- and 355-nm laser) and describe implementation of UVPD in a pressure linear quadrupole ion trap in a QTOF instrument. UVPD (266-nm laser) has also been implemented in the transfer cell region of a travelling wave ion mobility spectrometry (TWIMS)-enabled Q-ToF mass spectrometer [11].

More recently, an Orbitrap mass spectrometer became commercially available with different activation methods including UVPD (213-nm laser), photodissociation occurring in the linear ion trap [12]. The application of UVPD has also been recently reported on Omnitrap platform [13].

A large variety of light sources have been used for UVPD and cover a wide range of wavelengths (150–400 nm) including excimer laser, solid-state lasers, gas-discharge lamps, synchrotron radiation and light-emitting diodes [4]. UPVD is generally performed on intact molecules, but derivatization approaches have been developed and extended to the visible range using chromophore tags for improved detection selectivity of the analytes [9, 14].

Despite the technological advances made in terms of combination between mass spectrometers and UVPD, most published applications [4] focus on proteins, peptides, lipids and carbohydrates where UVPD provides improved information or selectivity compared to CID but limited work has been reported on LMWC. Untargeted LC–MS screening of organic micro-pollutants (*n* = 46) in water samples on a Orbitrap Fusion Lumos equipped with UVPD showed that informative fragments could be generated at 213 nm for compounds that poorly fragment with HCD. Derivatization of a telmisartan, a pharmaceutical compound, with an -idine containing reagent showed at 213-nm rich UVPD spectra compared to HCD which yielded a single fragment [15]. Comparisons of UVPD and CID spectra on a QqTOF system using a set of aromatic chromophore-containing pharmaceutical compounds (desmethyl bosentan, haloperidol, nelfinavir) showed distinct fragments resulting from photodissociation at 266 or 355 nm [16].

The first part of the present work describes the implementation of a 266-nm Nd:YaG laser on a triple quadrupole linear ion trap with a curved collision cell which enables the combination of different UVPD and CID acquisition schemes in the same LC–MS analysis. The performance of both activation methods is compared for a mix of 90 molecules from different classes of LMWC compounds including peptides, pesticides, pharmaceuticals, metabolites and drugs of abuse using different quadrupole and trap acquisition modes. In a second part, LC–MS/MS quantification of bosentan and its desmethyl metabolite in human plasma, as model compounds, with confirmatory analysis is investigated. Two acquisition schemes were explored: (i) quantification in the selected reaction monitoring mode and enhanced product ion scan in CID and UVPD for confirmatory analysis, (ii) quantification in enhanced product ion scan with only UVPD or with combined UVPD and CID.

## Material and methods

### Chemicals

A custom mix of 90 molecules (mix90) of different concentration level used for analysis was produced by Sciex (AB Sciex, Concord, ON, Canada) (Table S1). Standards of bosentan and its desmethyl metabolite and their deuterated (d4) analogues were obtained from F. Hoffmann-La Roche (Basel, Switzerland). Stock solutions of 1 mg/mL were prepared in methanol and stored at 5 °C.

Water (UHPLC-MS grade) was purchased from Huberlab (Aesch, Switzerland). Methanol (HPLC grade) was provided by Fisher Scientific AG (Reinach, Switzerland), acetonitrile by VWR Chemicals (Darmstadt, Germany) and formic acid (FA) by Merck (Darmstadt, Germany).

Anonymized pooled human plasma samples (4 donors), used for quantification experiments, were obtained from human blood donors from the Centre de Transfusion Sanguine (HUG, Geneva, Switzerland).

### Standards solutions, calibrants and quality control samples

For the analysis of the mix90, the stock solution was diluted ten times with mobile phase prior to injection. Calibration samples were prepared by spiking bosentan and its desmethyl metabolite in EDTA human plasma to obtain final concentration of 0.5, 1, 5, 10, 30, 50, 100, 300 and 500 ng/mL. Six quality control samples were prepared at the following concentrations: 0.5, 1.5, 5, 15, 50, 500 ng/mL in EDTA human plasma.

### Plasma sample preparation

To 50 μL of plasma, 5 μL of IS (200 ng/mL) was added followed by 100 μL of cold acetonitrile for protein precipitation. The samples were centrifuged (14,000 rpm,10 min, 4 °C) (Megafuge 1.0R, Heraeus Instruments, Schaffhausen. Switzerland) and the transferred supernatant was evaporated until dryness in a vacuum centrifuge at 55 °C until dryness (UNIVAPO 150 ECH, BioLabo, Châtel-St-Denis, Switzerland). The extracts were reconstituted in 50 μL mixture of 40/60 ACN/H_2_O v/v and 0.1% of FA and vortexed for 30 s and centrifuged prior analysis.

### Liquid chromatography

The chromatographic system consisted of an HTC PAL autosampler (CTC Analytics, Zwingen, Switzerland), equipped with a 5-μL injection loop, and a high-pressure liquid chromatographic Nexera LC-30AD UHPLC pump equipped with a low-pressure gradient unit (Shimadzu, Kyoto, Japan). Chromatographic separation was performed on a reverse-phase Luna C18 column (100 mm length, 2 mm I.D., 2.5 μm particle size, Phenomenex) protected by a C18 guard column (Phenomenex, Switzerland).

The LC mobile phase were A — 0.1% formic acid in H_2_O and B — 0.1% formic acid in CH_3_CN. For the mix90, two different gradients were applied at a flow rate of 0.2 mL/min. Gradient 1: 3% B increased after 1 min to 90% B in 15 min and hold for 3 min and gradient 2: 3% B increased after 1 min to 90% B in 28 min and hold for 3 min. For the analysis of bosentan and its desmethyl metabolite, the flow rate was 0.3 mL/min and the gradient started at 40% B for 0.5 min and increased to 90% B in 2.5 min and hold for 0.5 min.

### Mass spectrometry–ultraviolet photodissociation and collision-induced dissociation

**Fig. 1:**
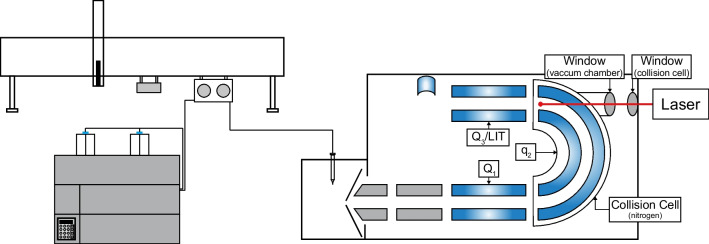
Schematic of the UVPD implementation on the triple quadrupole linear ion trap (QTrap 6500^+^). The instrument was modified by adding two ultraviolet windows to allow the laser pulse to dissociate the selected precursor ions at the end of the curved collision cell (q2)

A QTrap 6500^+^, hybrid triple quadrupole and linear ion trap (Sciex, Concord, ON, Canada), was used to perform the analyses. Ionization, in positive mode, was achieved with a Turbo V ion source (Sciex). The source parameters were as follows: temperature (TEM) 300 °C, ion spray voltage (IS) 5500 V, curtain gas (CUR), ion gas source 1 and 2 (GS1, GS2) 20 psi, and declustering potential (DP) 80*.* For mix90, data were acquired in data-dependent acquisition (DDA) mode using dynamic fill time (DFT) and dynamic background subtraction (DBS). The DDA criteria intensity threshold was at 10,000 and analytes were never excluded or excluded after 4 occurrences, dependent on the experiment. Figure 1 provides a schematic of the modified instrument on which UVPD experiments were performed. Modifications were made to the vacuum chamber of the instrument to allow the laser pulse to dissociate the selected precursor ions at the end of the curved collision cell (q2) by adding two ultraviolet windows (High transmission UV Grade Calcium Fluoride CaF_2_). The laser was a 266-nm Nd:YaG laser (Teem Photonics S.A, Meylan, France) with a power of ~ 0.5 µJ (4.7 eV per photon) and a frequency of 19 kHz. Ions are focused as an ion cloud by collisional cooling at the end of q2 prior irradiation by the laser. Collisional cooling increases the probability of interaction between the ions and the UV photons and provides higher fragmentation yield. UVPD occurs at the same spatial location where the ions are irradiated. Fragments are then transmitted in the linear ion trap to be trapped and detected. UVPD experiments were generally performed with an irradiation time of 170 ms and the Analyst software (1.7.1) was modified to perform UVPD and CID enhanced product experiments (EPI) in the same LC–MS run. For multiple reaction monitoring in CID mode, following transitions were used: bosentan *m/z* 552.2 → *m/z* 202.1, d4-bosentan *m/z* 556.2 → *m/z* 202.1, desmethyl bosentan *m/z* 538.2 → *m/z* 494.2, d4-desmethyl bosentan *m/z* 542.2 → *m/z* 494.2, while for UVPD quantification in enhanced product ion mode *m/z* 508.2 and *m/z* 322.2 were used respectively for bosentan and desmethyl bosentan (Table S2). Nevertheless, MRM CID transition of the internal standard were used to build EPI UVPD calibration curve with the following area ratio: analyte/internal standards. Peakview software (Sciex, version 2.0 and 2.2) was used to process all LC–MS/MS acquisition. For quantification of bosentan and its metabolite, in MRM and EPI mode, Multiquant 2.0 software (Sciex) was used with the MQ4 algorithm using 1/*x* for weighting and a gaussian smoothing of 1.

## Results and discussion

**Fig. 2:**
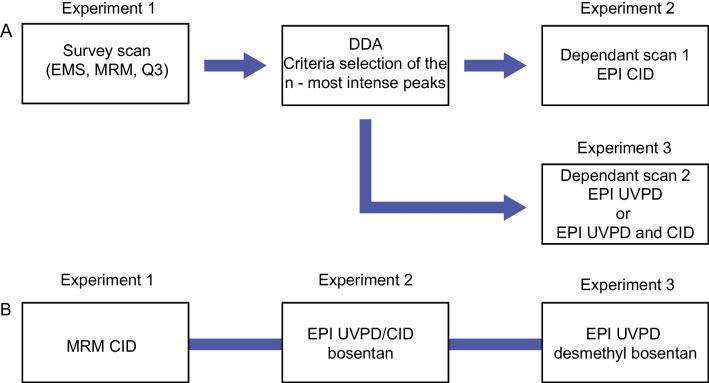
LC-MS/MS workflow with CID and UVPD for quantitative and qualitative analysis (**A**) and quantitative analysis (**B**)

Various acquisition scenarios were considered to enable qualitative and quantitative analysis (QUAL/QUANT) and are summarized in Fig. 2. The first workflow (Fig. 2A) was based on data-dependent acquisition mode with Q3, Enhanced MS or MRM as survey scan followed by enhanced product ion (EPI) with CID and UVPD as dependent scan. This workflow was used to generated spectra of mix90 as well as for quantitative analysis of bosentan in plasma. The MS cycle time for different analyses was in the range 611 to 912 ms. The second workflow (Fig. 2B) was designed for targeted quantitative analysis with CID/MRM, CID and UVPD EPI spectra in the same LC–MS/M analysis.

### Comparison of UVPD and CID fragmentation

**Fig. 3:**
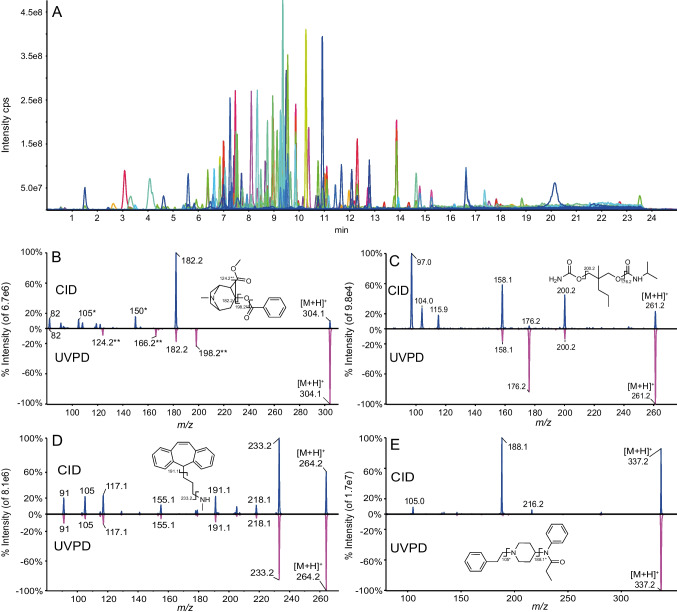
**A** Overlay of extracted ion chromatogram traces of mix90. **B**–**E** Product ion spectra of representative analytes, top panel CID — 30 eV and bottom panel UVPD — 170 ms irradiation time **B** cocaine, **C** carisoprodol, **D** protriptyline and **E** fentanyl. CID (*)/UVPD (**) specific and common (no asterisk) proposed fragments are highlighted on their structures

The mix90 (*n* = 90 compounds) used in this work is made up of different types of molecules (pesticides, peptides, illicit drugs and pharmaceuticals). The product ion spectra were recorded at 30 eV for CID and for UVPD laser at 266 nm operated at a fixed frequency of 19 kHz with 170-ms irradiation time (Fig. 3 and Figures S1–S85). Most of the analytes did produce product spectra in both modes. Tapentadol and erythromycin did fragment in CID at 30 eV but did not generate significative fragments in UVPD. Tamoxifen and buprenorphine neither generated good fragmentation in both modes. All compounds could be classified into main categories: those which generate similar spectra and those which generate in addition to common CID fragments unique UVPD fragments. Representative product ion spectra are presented in Fig. 3 for cocaine, carisoprodol, protriptyline and fentanyl. For cocaine, the fragment at *m/z* 182.1 is common to both fragmentation modes while those at *m/z* 198.2, *m/z* 166.2 and *m/z* 124.2 were found to be unique for UVPD (Fig. 3B). Carisoprodol (Fig. 3C) does not shows unique fragments for UVPD. For protriptyline (Fig. 3D), similar product ion spectra were recorded while for fentanyl (Fig. 3E) no fragment could be observed in UVPD.

Collision-induced dissociation spectra are generated by the gas interaction between a neutral gas (N_2_) and an energized precursor ion. In comparison, ultraviolet photodissociation is based on the absorption of photons by the precursor ions. Obviously, the photon interaction is only effective if the molecule has chromophores which enable the absorption at the wavelength of the laser, in our case at 266 nm. However, if the condition is satisfied, the photodissociation will occur by a direct dissociation phenomenon close to the absorption site but can also occur somewhere else on the molecule by an internal conversion as for CID.

**Fig. 4:**
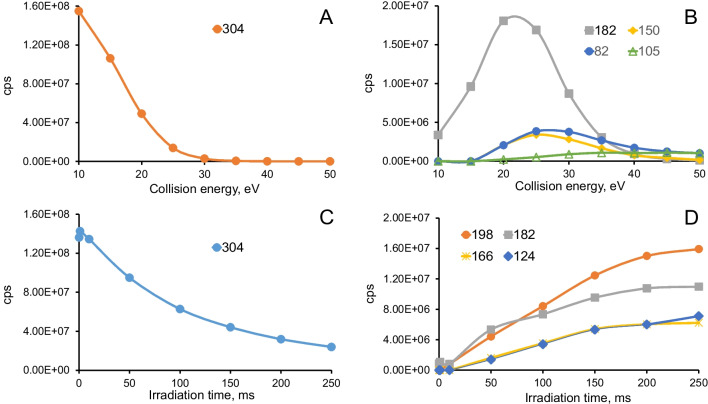
Signal intensities of cocaine precursor and fragments versus collision energy (CID) and irradiation time (UVPD). **A** CID: precursor ions of cocaine (10–50 eV) and **B** CID: selected fragments of cocaine, **C** UVPD precursor ions of cocaine (0.1–250 ms), **D** UVPD: main fragments of cocaine

Figure 4 compares the cocaine precursor and fragments intensities versus collision energy (10 to 50 eV) for CID and versus irradiation time (0.1 to 250 ms) for UVPD. On the QTrap system for standard operation and for UVPD, the collision cell is set at 10 eV to maintain ion transmitted through the cell and does not generate significant CID fragments for most analytes. For both methods, a decrease of precursor ion is observed (Fig. 4A, C), but less pronounced for UVPD. In CID, the formation of fragments is dependent on the CE applied (> 10 eV) as observed for cocaine at *m/z* 182 and *m/z* 150 (Fig. 4B). Those fragments go through a maximum while the precursor disappears after 30 eV. On the other hand, in UVPD, the appearance of cocaine fragments is not dependent on the irradiation time but only the intensities (Fig. 4D). No significant signal intensity increase is observed after 100 ms (within factor of 2). A similar behaviour is observed for benzoylecgonine and for haloperidol (Figures S86–S93).

**Fig. 5:**
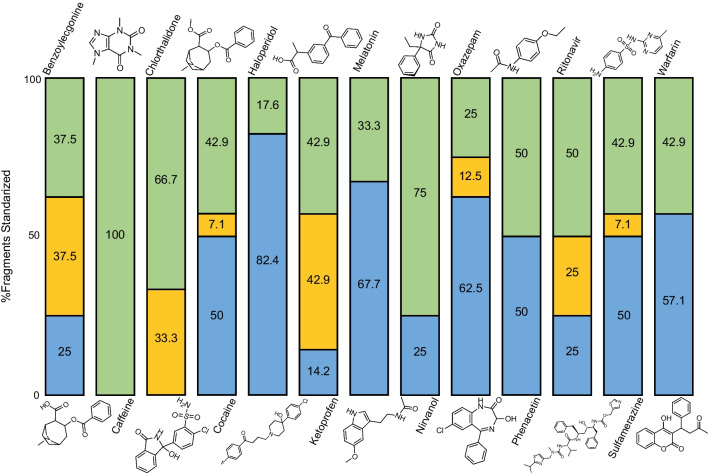
Fragment distribution (% fragments standardized) according for 13 representative molecules from the mix90 where the green colour represents the common fragments between UVPD (170-ms irradiation time) and CID (30 eV), orange the CID-specific fragments and blue the UVPD-specific fragments

The complementarity of both fragmentation techniques is illustrated in Fig. 5 which shows the fragmentation distribution of a set of 13 analytes from the mix90. The data collected in this bar chart has been acquired at fixed collision energy of 30 eV and fixed irradiation time of 170 ms separately and then merged by comparing the fragments observed above a threshold of 5% in both UVPD and CID spectra. In summary, it is observed that both fragmentation techniques generate for the analytes (i) the same fragments, (ii) specific CID fragments or (iii) specific UVPD fragments with similar sensitivities. As both spectra can be acquired in a single LC–MS analysis, using the two fragmentation techniques can improve compound identification/characterization or enhance the selectivity for quantitative analysis.

### Ultraviolet photodissociation and collision-induced dissociation for qualitative and quantitative LC–MS analysis of bosentan and its desmethyl metabolite in human plasma

**Fig. 6:**
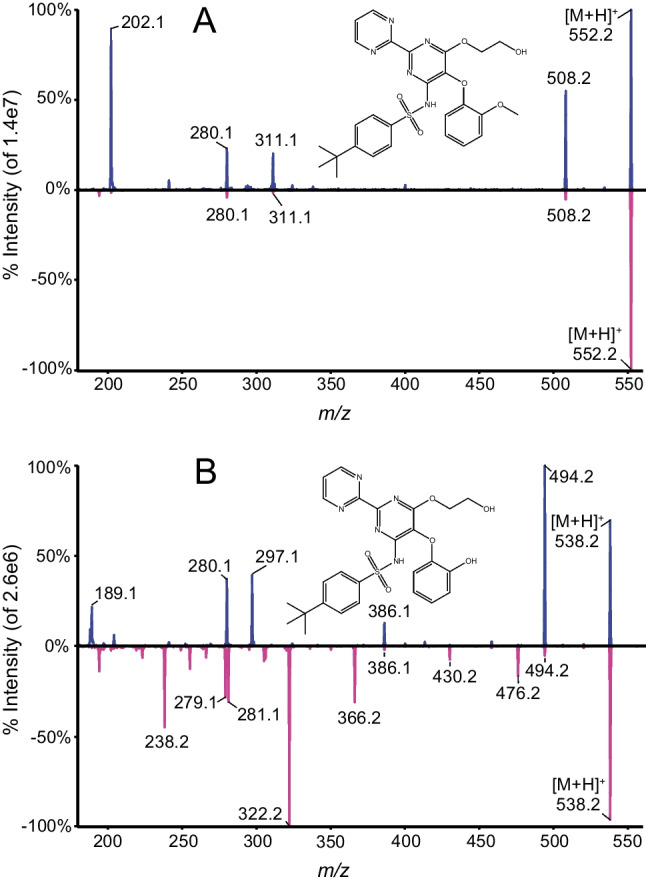
Product ion spectra of **A** bosentan and **B** its desmethyl metabolite, top panel CID (30 eV) and bottom panel UVPD with 170-ms irradiation time

The potential of quantitative LC–MS/MS analysis using UVPD or combined CID/UVPD has been investigated for the analysis in human plasma on bosentan and its desmethyl metabolite after protein precipitation. Many different LC–MS assays have been reported for the quantification of bosentan and its metabolites in human plasma [17]. The purpose of the present work was not to develop a novel LC–MS assay for bosentan based on UVPD but to demonstrate its potential on well-characterized analytes; therefore, full assay validation was not performed [18]. The CID fragmentation of bosentan and its metabolites has been extensively explored [19] and generates specific fragments at *m/z* 311, 280 and 202 for bosentan and at *m/z* 297, 280 and 189 for desmethyl bosentan. Hao et al*.* [16] reported the UVPD fragmentation of desmethyl bosentan in a pressurized linear quadrupole ion trap at two different wavelengths (266 and 355 nm) and observed unique fragments at *m/z* 430, 366 and 322. The product ion spectra of desmethyl bosentan in CID at 30 eV (lower spectrum) and UVPD at 266 nm (upper spectrum) is presented in Fig. 6B. The presence of unique fragments at *m/z* 366 and *m/z* 322 can be observed. Collision energy and irradiation profiles are shown in Figures S94 to S97. Interestingly bosentan did not fragment well in UVPD at 266 nm despite that both analytes have similar structures and contain conjugated p-bonds in the form of (hetero) aromatic rings and were therefore expected to undergo UV absorptions.

### Workflow A: LC–MS quantification in CID multiple reaction mode (MRM) and CID and UVPD scan for confirmation

The data-dependent acquisition (DDA) mode was used with CID multiple reaction monitoring as survey scan for quantification and two enhanced product ion scans, one using CID and another one using UVPD/CID as dependent scan for confirmatory analysis. Bosentan and desmethyl bosentan could be quantified over 3 orders of magnitude from 0.5 up to 500 ng/mL. Accuracy and precision based on quality control samples are presented in Table 1 and are within acceptable values (< 15%) for bioanalytical work.

**Table 1:** Accuracy and precision obtained from the analysis of human plasma quality control samples spiked with bosentan and its metabolite analysed by LC-MRM CID. *LOQQC* limit of quantification QC, *LQC* low QC, *MQC* medium QC, *HQC* high QC used for calibration range 0.5 to 500 ng/mL

	Amount added (ng/mL)	Bosentan		Desmethyl bosentan		*n*
		CV (%)	Accuracy (%)	CV (%)	Accuracy (%)	
LOQQC1	0.5	8.6	91.9	7.1	91.5	5
LQC1	1.5	4.9	97.9	6	99.6	5
MQC1	50	5.5	97.4	2.8	100	5
HQC1	500	2.9	90.4	1.8	96.1	5

**Fig. 7:**
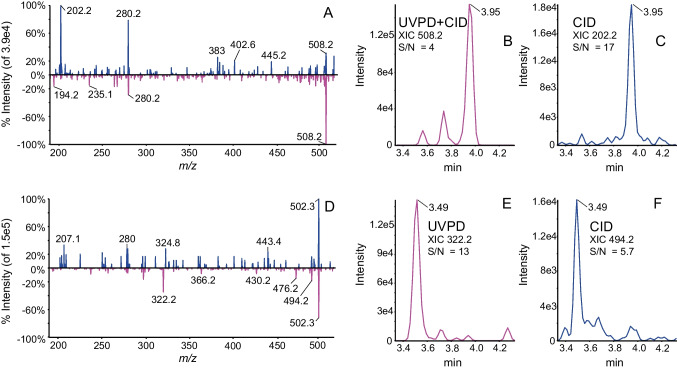
LC–MS analysis of human plasma sample at the LOQ (0.5 ng/mL). **A** Enhanced product ion spectra (zoom *m/z* 180–520) of bosentan top panel CID at 40 eV and bottom panel with UVPD and CID at 30 eV. **B** XIC of *m/z* 508 fragment of bosentan obtained by UVPD/CID conditions. **C** XIC of *m/z* 202 fragment of bosentan obtained by CID. **D** Enhanced product ion spectra (zoom *m/z* 180–520) of desmethyl bosentan top CID at 40 eV and bottom UVPD with CID at 30 eV. **D** XIC of fragment *m/z* 322.2 from desmethyl bosentan obtained at 40 eV and **E** XIC of fragment *m/z* 322.2 from desmethyl bosentan obtained by UVPD and **F** XIC of fragment *m/z* 494.2 obtained by CID at 40 eV

Figure 7 presents a representative LC–MS analysis of a human plasma spiked at the LOQ with 0.5 ng/mL of each analyte. As the laser is focused on the end of the curved collision cell, precursors ions can be fragmented first by CID and the residual precursor ion by UVPD. CID fragment ions can be also fragmented by UVPD where a combination of 30-eV collision energy and a 170-ms irradiation time was applied as shown for bosentan in Fig. 7A. Figure 7B and C present the XIC of the most intense fragment of bosentan under UVPD/CID and CID, respectively. In the case of bosentan, UVPD is not very informative for confirmatory analysis while for CID good quality spectrum is obtain. For desmethyl bosentan, a completely different situation is observed as illustrated in Fig. 7D. Figure 7E and F present the XIC of the most intense fragment of desmethyl bosentan under UVPD/CID and CID, respectively. At 0.5 ng/mL, the LOQ, the CID spectrum does not show a good quality spectrum while the UVPD/CID spectrum enables to confirm the presence of desmethyl bosentan in the human plasma. This experiment illustrates the complementarity of both fragmentation techniques which are applied in the same LC–MS analysis for analyte confirmation.

### Workflow B: CID and UVPD LC–MS/MS quantification in enhanced product ion (EPI) scan

**Fig. 8:**
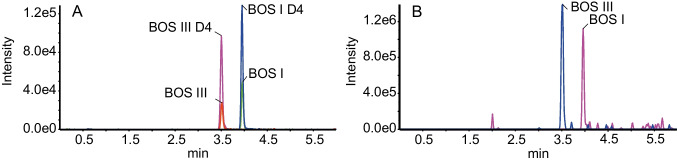
Comparison LC–MS traces of human plasma spiked with bosentan and desmethyl bosentan at 5 ng/mL. **A** MRM bosentan (BOS I) *m/z* 552 → 202 and desmethyl bosentan (BOS III) *m/z* 538 → 508. **B** Enhanced product ion XIC trace at *m/z* 508.2 of bosentan fragment obtained with UVPD and XIC trace of fragment desmethyl bosentan of at *m/z* 332.2 obtained by UVPD

Due to the 266-nm laser characteristics used in the work, it was not possible to perform MRM acquisition in UVPD mode, and therefore, enhanced product ion scan was used to investigate quantitative performance in UVPD. Three experiments were defined as depicted in Fig. 2B. The first experiment includes four MRM transitions: two for bosentan and its d4-IS and two for desmethyl bosentan and its d4-IS. The second experiment was an enhanced product ion scan for bosentan with CID/UVPD and the third experiment was also an enhanced product ion scan for desmethyl bosentan with UVPD. Representative chromatograms for human plasma spiked with 5 ng/mL of each analyte are shown in Fig. 8. For quantification, area ratio between the analyte acquired in EPI mode and the d4-IS acquired in MRM mode was used. The response was found to be linear over two orders of magnitude. The limited dynamic range is mainly due to space charging effects in EPI mode. Precision and accuracy between 5 and 500 ng/mL for human plasma quality control samples are provided in Table 2 and found to be acceptable.

**Table 2:** Accuracy and precision obtained from the analysis of human plasma quality control samples spiked with bosentan and its metabolite analysed by LC with enhanced product ion mode CID/UVPD and UVPD. *LOQQC* limit of quantification QC, *LQC* low QC, *MQC* medium QC, *HQC* high QC used for calibration range 5 to 500 ng/mL

	Amount added (ng/mL)	Bosentan (CID + UVPD)		Desmethyl bosentan (UVPD)		*n*
		CV (%)	Accuracy (%)	CV (%)	Accuracy (%)	
LOQQC2	5	16.7	112	7.6	116	5
LQC2	15	11.1	108	7.5	95.8	5
MQC2	50	9.2	99.8	7.6	91.1	5
HQC2	500	5	112	4.8	121	5

The present investigation illustrated that CID and UVPD fragmentation can also be used with different acquisition strategies for quantitative analysis. One major benefit is the possibility to get different assay selectivity either because the interference background is different between UVPD and CID or because UVPD generates unique fragments compared to CID.

## Conclusions

Ultraviolet photodissociation with a 266-nm laser has been successfully implemented to a triple quadrupole linear ion trap instrument with a curved collision cell. UVPD and CID spectra were compared for a mix of 90 low molecular weight compounds. These two activation methods offer complementary fragments as well as common fragments with similar sensitivities for most analytes investigated using a 266-nm laser. While in CID the MS/MS spectrum are dependent on collision energy applied, in UVPD, irradiation time affects mostly the intensity of the MS/MS spectrum. An irradiation time between 50 and 100 ms was found to be a practical value which make UVPD compatible with a LC time scale. Therefore, several experiments’ combinations, as combining UVPD and CID, can be considered in the same LC–MS analysis. Work is in progress to compare the performance difference of a more energetic laser at 213 nm laser versus the 266 nm for low molecular weight compounds analysis. Also, the use of UVPD MS/MS libraries in addition to CID MS/MS libraries could be used to improve the identification/confirmation of pharmaceuticals, drug of abuse or pesticides in complex samples. The versatility of UVPD and CID was also demonstrated for quantitative analysis of bosentan and its desmethyl metabolite, used as model analytes, in human plasma. Under CID or UVPD, different background signals are observed for both fragmentation methods. Unique fragments could be highlighted which opens the possibility to develop selective quantitative assay with an improved sample throughput, for analytes present in different matrices. Implementation of multimodal fragmentation techniques on low-resolution or high-resolution instruments such as quadrupole time of flight [20] opens the possibility of improved structural information in qualitative analysis as well as improved selectivity in quantitative analysis on a LC time scale.

### Supplementary Information

Below is the link to the electronic supplementary material.

Supplementary file1 (PDF 5388 KB)
