# Supplementary material for: Ultraviolet photodissociation and collision-induced dissociation for qualitative/quantitative analysis of low molecular weight compounds by liquid chromatography-mass spectrometry
Source: Anal Bioanal Chem. 2023 Oct 7;415(29-30):7117–26. doi: 10.1007/s00216-023-04977-0 (PMC10684635; doi:10.1007/s00216-023-04977-0)
Supplement: Supplementary file 1 — Supplementary file1 (PDF 5388 KB) [file 216_2023_4977_MOESM1_ESM.pdf]

Supporting Information

Ultraviolet Photodissociation and Collision Induced Dissociation for Qualitative/Quantitative Analysis of Low Molecular Weight Compounds by Liquid Chromatography-Mass Spectrometry

Romain Giraud[1], Yves LeBlanc[2], Mircea Guna[2], Gérard Hopfgartner[1]*

[1]*Life Sciences Mass Spectrometry, Department of Inorganic and Analytical Chemistry, University of Geneva, 24 Quai Ernest Ansermet, CH-1205, Geneva 4, Switzerland*
[2]*SCIEX, Toronto, Ontario, Canada*

*corresponding author e-mail: gerard.hopfgartner@unige.ch

Table of Contents:

FIGURE S1. BENTAZON CID (UPPER) AND UVPD (LOWER) FRAGMENTATION SPECTRA ZOOM FROM 80 TO 250 M/Z. 10
FIGURE S2. 7-AMINOCLONAZEPAM CID (UPPER) AND UVPD (LOWER) FRAGMENTATION SPECTRA ZOOM FROM 80 TO 320 M/Z. 10
FIGURE S3. BUSCOPAN CID (UPPER) AND UVPD (LOWER) FRAGMENTATION SPECTRA ZOOM FROM 80 TO 380 *M/Z*. 11
FIGURE S4. BENZOYLECGONINE CID (UPPER) AND UVPD (LOWER) FRAGMENTATION SPECTRA ZOOM FROM 80 TO 310 *M/Z*. 11
FIGURE S5. CAFFEINE CID (UPPER) AND UVPD (LOWER) FRAGMENTATION SPECTRA ZOOM FROM 80 TO 210 *M/Z*. 12
FIGURE S6. HALOPERIDOL CID (UPPER) AND UVPD (LOWER) FRAGMENTATION SPECTRA ZOOM FROM 80 TO 400 *M/Z*. 12
FIGURE S7. CHLORTHALIDONE CID (UPPER) AND UVPD (LOWER) FRAGMENTATION SPECTRA ZOOM FROM 80 TO 370 *M/Z*. 13
FIGURE S8. MELATONIN CID (UPPER) AND UVPD (LOWER) FRAGMENTATION SPECTRA ZOOM FROM 80 TO 260 *M/Z*. 14
FIGURE S9. KETOPROFEN CID (UPPER) AND UVPD (LOWER) FRAGMENTATION SPECTRA ZOOM FROM 80 TO 270 *M/Z*. 14
FIGURE S10. SULFAMERAZINE CID (UPPER) AND UVPD (LOWER) FRAGMENTATION SPECTRA ZOOM FROM 80 TO 280 *M/Z*. 15
FIGURE S11. NIRVANOL CID (UPPER) AND UVPD (LOWER) FRAGMENTATION SPECTRA ZOOM FROM 80 TO 220 *M/Z*. 16
FIGURE S12. PHENACETIN CID (UPPER) AND UVPD (LOWER) FRAGMENTATION SPECTRA ZOOM FROM 80 TO 195 *M/Z*. 16
FIGURE S13. OXAZEPAM CID (UPPER) AND UVPD (LOWER) FRAGMENTATION SPECTRA ZOOM FROM 80 TO 300 *M/Z*. 17
FIGURE **S14.** WARFARIN CID (UPPER) AND UVPD (LOWER) FRAGMENTATION SPECTRA ZOOM FROM 80 TO 320 *M/Z*. 17
FIGURE S15. RITONAVIR CID (UPPER) AND UVPD (LOWER) FRAGMENTATION SPECTRA ZOOM FROM 80 TO 750 *M/Z*. 19
FIGURE S16. BUSPIRONE CID (UPPER) AND UVPD (LOWER) FRAGMENTATION SPECTRA ZOOM FROM 80 TO 405 *M/Z*. 19
FIGURE S17. BUFURALOL CID (UPPER) AND UVPD (LOWER) FRAGMENTATION SPECTRA ZOOM FROM 80 TO 295 *M/Z*. 20
FIGURE S18. ACETAMIPRID CID (UPPER) AND UVPD (LOWER) FRAGMENTATION SPECTRA ZOOM FROM 80 TO 240 *M/Z*. 20
FIGURE S19. TYLOSIN CID (UPPER) AND UVPD (LOWER) FRAGMENTATION SPECTRA ZOOM FROM 80 TO 930 *M/Z*. 22
FIGURE S20. AMITRIPTYLINE CID (UPPER) AND UVPD (LOWER) FRAGMENTATION SPECTRA ZOOM FROM 80 TO 305 *M/Z*. 22
FIGURE S21. CHLORPROTHIXENE CID (UPPER) AND UVPD (LOWER) FRAGMENTATION SPECTRA ZOOM FROM 80 TO 345 *M/Z*....23

FIGURE S22. CARBAMAZEPINE CID (UPPER) AND UVPD (LOWER) FRAGMENTATION SPECTRA ZOOM FROM 80 TO 275 *M/Z*. 23
FIGURE S23. ALPRAZOLAM CID (UPPER) AND UVPD (LOWER) FRAGMENTATION SPECTRA ZOOM FROM 80 TO 325 *M/Z*. 24
FIGURE S24. TAMOXIFEN CID (UPPER) AND UVPD (LOWER) FRAGMENTATION SPECTRA ZOOM FROM 80 TO 390 *M/Z*. 25
FIGURE S25. TOLBUTAMIDE CID (UPPER) AND UVPD (LOWER) FRAGMENTATION SPECTRA ZOOM FROM 80 TO 290 *M/Z*. 25
FIGURE S26. DIAZEPAM CID (UPPER) AND UVPD (LOWER) FRAGMENTATION SPECTRA ZOOM FROM 80 TO 305 *M/Z*. 26
FIGURE S27. ANGIOTENSIN II 2+ CID (UPPER) AND UVPD (LOWER) FRAGMENTATION SPECTRA ZOOM FROM 80 TO 780 *M/Z*. 27
FIGURE S28. ANGIOTENSIN I 3+ CID (UPPER) AND UVPD (LOWER) FRAGMENTATION SPECTRA ZOOM FROM 80 TO 780 *M/Z*. 27
FIGURE S29. ACEBUTOLOL CID (UPPER) AND UVPD (LOWER) FRAGMENTATION SPECTRA ZOOM FROM 80 TO 360 *M/Z*. 28
FIGURE S30. CLENBUTEROL CID (UPPER) AND UVPD (LOWER) FRAGMENTATION SPECTRA ZOOM FROM 80 TO 290 *M/Z*. 29
FIGURE S31. AZITHROMYCIN CID (UPPER) AND UVPD (LOWER) FRAGMENTATION SPECTRA ZOOM FROM 80 TO 780 *M/Z*. 29
FIGURE S32. BUPRENORPHINE CID (UPPER) AND UVPD (LOWER) FRAGMENTATION SPECTRA ZOOM FROM 80 TO 500 *M/Z*. 30
FIGURE S33. DESIPRAMINE CID (UPPER) AND UVPD (LOWER) FRAGMENTATION SPECTRA ZOOM FROM 80 TO 290 *M/Z*. 30
FIGURE S34. CHLORAMPHENICOL CID (UPPER) AND UVPD (LOWER) FRAGMENTATION SPECTRA ZOOM FROM 80 TO 370 *M/Z*....................31
FIGURE S35. BENDROFLUMETHIAZIDE CID (UPPER) AND UVPD (LOWER) FRAGMENTATION SPECTRA ZOOM FROM 80 TO 455 *M/Z*....................31
FIGURE S36. L-TRYPTOPHAN CID (UPPER) AND UVPD (LOWER) FRAGMENTATION SPECTRA ZOOM FROM 80 TO 250 *M/Z*. 32
FIGURE S37. MINOXIDIL CID (UPPER) AND UVPD (LOWER) FRAGMENTATION SPECTRA ZOOM FROM 80 TO 225 *M/Z*. 33
FIGURE S38. LAMOTRIGINE CID (UPPER) AND UVPD (LOWER) FRAGMENTATION SPECTRA ZOOM FROM 80 TO 275 *M/Z*. 33
FIGURE S39. TAPENTADOL CID (UPPER) AND UVPD (LOWER) FRAGMENTATION SPECTRA ZOOM FROM 80 TO 240 *M/Z*. 34
FIGURE S40. ZOLPIDEM CID (UPPER) AND UVPD (LOWER) FRAGMENTATION SPECTRA ZOOM FROM 80 TO 315 *M/Z*. 34
FIGURE S41. LACOSAMIDE CID (UPPER) AND UVPD (LOWER) FRAGMENTATION SPECTRA ZOOM FROM 80 TO 315 *M/Z*. 35
FIGURE S42. URAPIDIL CID (UPPER) AND UVPD (LOWER) FRAGMENTATION SPECTRA ZOOM FROM 80 TO 400 *M/Z*. 36
FIGURE S43. IMIDACLOPRID CID (UPPER) AND UVPD (LOWER) FRAGMENTATION SPECTRA ZOOM FROM 80 TO 270 *M/Z*. 37
FIGURE S44. ERYTHROMYCIN CID (UPPER) AND UVPD (LOWER) FRAGMENTATION SPECTRA ZOOM FROM 80 TO 840 *M/Z*. 38

FIGURE S45. KETOCONAZOLE CID (UPPER) AND UVPD (LOWER) FRAGMENTATION SPECTRA ZOOM FROM 80 TO 530 *M/Z*. 38
FIGURE S46. BROMOCRIPTINE CID (UPPER) AND UVPD (LOWER) FRAGMENTATION SPECTRA ZOOM FROM 80 TO 840 *M/Z*. 39
FIGURE S47. METHADONE CID (UPPER) AND UVPD (LOWER) FRAGMENTATION SPECTRA ZOOM FROM 80 TO 320 *M/Z*. 40
FIGURE S48. LOPERAMIDE CID (UPPER) AND UVPD (LOWER) FRAGMENTATION SPECTRA ZOOM FROM 80 TO 500 *M/Z*. 40
FIGURE S49. TERFENADINE CID (UPPER) AND UVPD (LOWER) FRAGMENTATION SPECTRA ZOOM FROM 80 TO 480 *M/Z*. 41
FIGURE S50. KETOROLAC CID (UPPER) AND UVPD (LOWER) FRAGMENTATION SPECTRA ZOOM FROM 80 TO 300 *M/Z*. 42
FIGURE S51. GLYCYRRHIZIC ACID CID (UPPER) AND UVPD (LOWER) FRAGMENTATION SPECTRA ZOOM FROM 80 TO 840 *M/Z*....................42
FIGURE S52. TEMAZEPAM CID (UPPER) AND UVPD (LOWER) FRAGMENTATION SPECTRA ZOOM FROM 80 TO 315 *M/Z*. 43
FIGURE S53. TESTOSTERONE CID (UPPER) AND UVPD (LOWER) FRAGMENTATION SPECTRA ZOOM FROM 80 TO 340 *M/Z*. 44
FIGURE S54. EPITESTOSTERONE CID (UPPER) AND UVPD (LOWER) FRAGMENTATION SPECTRA ZOOM FROM 80 TO 340 *M/Z*. 45
FIGURE S55. TAUROCHOLIC ACID CID (UPPER) AND UVPD (LOWER) FRAGMENTATION SPECTRA ZOOM FROM 80 TO 540 *M/Z*....................45
FIGURE S56. ETODOLAC CID (UPPER) AND UVPD (LOWER) FRAGMENTATION SPECTRA ZOOM FROM 80 TO 295 *M/Z*. 46
FIGURE S57. FLUSILAZOLE CID (UPPER) AND UVPD (LOWER) FRAGMENTATION SPECTRA ZOOM FROM 80 TO 330 *M/Z*. 47
FIGURE S58. LOVASTATIN CID (UPPER) AND UVPD (LOWER) FRAGMENTATION SPECTRA ZOOM FROM 80 TO 425 *M/Z*. 48
FIGURE S59. MIDAZOLAM CID (UPPER) AND UVPD (LOWER) FRAGMENTATION SPECTRA ZOOM FROM 80 TO 350 *M/Z*. 48
FIGURE S60. MEPROBAMATE CID (UPPER) AND UVPD (LOWER) FRAGMENTATION SPECTRA ZOOM FROM 80 TO 250 *M/Z*. 49
FIGURE S61. INDOPROFEN CID (UPPER) AND UVPD (LOWER) FRAGMENTATION SPECTRA ZOOM FROM 80 TO 300 *M/Z*. 49
FIGURE S62. CLONAZEPAM CID (UPPER) AND UVPD (LOWER) FRAGMENTATION SPECTRA ZOOM FROM 80 TO 330 *M/Z*. 50
FIGURE S63. IBUPROFEN CID (UPPER) AND UVPD (LOWER) FRAGMENTATION SPECTRA ZOOM FROM 80 TO 250 *M/Z*. 51
FIGURE S64. SULFADIAZINE CID (UPPER) AND UVPD (LOWER) FRAGMENTATION SPECTRA ZOOM FROM 80 TO 280 *M/Z*. 52
FIGURE S65. TRAMADOL CID (UPPER) AND UVPD (LOWER) FRAGMENTATION SPECTRA ZOOM FROM 80 TO 275 *M/Z*. 52
FIGURE S66. SULFAMIDINE CID (UPPER) AND UVPD (LOWER) FRAGMENTATION SPECTRA ZOOM FROM 80 TO 310 *M/Z*. 53
FIGURE S67. PRAZOSIN CID (UPPER) AND UVPD (LOWER) FRAGMENTATION SPECTRA ZOOM FROM 80 TO 400 *M/Z*. 54

FIGURE S68. NORBUPRENORPHINE CID (UPPER) AND UVPD (LOWER) FRAGMENTATION SPECTRA ZOOM FROM 80 TO 455 *M/Z*. 55
FIGURE S69. PROPRANOLOL CID (UPPER) AND UVPD (LOWER) FRAGMENTATION SPECTRA ZOOM FROM 80 TO 275 *M/Z*. 55
FIGURE S70. PHENYLTOLOXAMINE CID (UPPER) AND UVPD (LOWER) FRAGMENTATION SPECTRA ZOOM FROM 80 TO 270 *M/Z*. 56
FIGURE S71. NORTRIPTYLINE CID (UPPER) AND UVPD (LOWER) FRAGMENTATION SPECTRA ZOOM FROM 80 TO 285 *M/Z*. 56
FIGURE S72. PROPAFENONE CID (UPPER) AND UVPD (LOWER) FRAGMENTATION SPECTRA ZOOM FROM 80 TO 360 *M/Z*. 57
FIGURE S73. VERAPAMIL CID (UPPER) AND UVPD (LOWER) FRAGMENTATION SPECTRA ZOOM FROM 80 TO 470 *M/Z*. 57
FIGURE S74. PREDNISOLONE CID (UPPER) AND UVPD (LOWER) FRAGMENTATION SPECTRA ZOOM FROM 80 TO 380 *M/Z*. 59
FIGURE S75. RESERPINE CID (UPPER) AND UVPD (LOWER) FRAGMENTATION SPECTRA ZOOM FROM 80 TO 640 *M/Z*. 59
FIGURE S76. SECOBARBITAL CID (UPPER) AND UVPD (LOWER) FRAGMENTATION SPECTRA ZOOM FROM 80 TO 270 *M/Z*. 60
FIGURE S77. RIFAMPICIN CID (UPPER) AND UVPD (LOWER) FRAGMENTATION SPECTRA ZOOM FROM 80 TO 840 *M/Z*. 60
FIGURE S78. NAPROXEN CID (UPPER) AND UVPD (LOWER) FRAGMENTATION SPECTRA ZOOM FROM 80 TO 270 *M/Z*. 61
FIGURE S79. PROGESTERONE CID (UPPER) AND UVPD (LOWER) FRAGMENTATION SPECTRA ZOOM FROM 80 TO 360 *M/Z*. 62
FIGURE S80. RITANILIC ACID CID (UPPER) AND UVPD (LOWER) FRAGMENTATION SPECTRA ZOOM FROM 80 TO 230 *M/Z*. 63
FIGURE S81. NORMEPERIDINE CID (UPPER) AND UVPD (LOWER) FRAGMENTATION SPECTRA ZOOM FROM 80 TO 245 *M/Z*. 63
FIGURE S82. PAMAQUINE CID (UPPER) AND UVPD (LOWER) FRAGMENTATION SPECTRA ZOOM FROM 80 TO 340 *M/Z*. 64
FIGURE S83. PERPHENAZINE CID (UPPER) AND UVPD (LOWER) FRAGMENTATION SPECTRA ZOOM FROM 80 TO 430 *M/Z*. 64
FIGURE S84. PROPOXYPHENE CID (UPPER) AND UVPD (LOWER) FRAGMENTATION SPECTRA ZOOM FROM 80 TO 430 *M/Z*. 65
FIGURE S85. NORSERTRALINE HCL CID (UPPER) AND UVPD (LOWER) FRAGMENTATION SPECTRA ZOOM FROM 80 TO 300 *M/Z*. 65
FIGURE S86. BENZOYLECGONINE FRAGMENTS INTENSITIES ACCORDING TO THE IRRADIATION TIME (MSEC) 66
FIGURE S87. BENZOYLECGONINE FRAGMENTS INTENSITIES ACCORDING TO THE COLLISION ENERGY (EV) 66
FIGURE S88. RESIDUAL BENZOYLECGONINE PRECURSOR INTENSITY ACCORDING TO THE IRRADIATION TIME (MSEC) 67
FIGURE S89. RESIDUAL BENZOYLECGONINE PRECURSOR INTENSITY ACCORDING TO THE COLLISION ENERGY (EV) 67
FIGURE S90. HALOPERIDOL FRAGMENTS INTENSITIES ACCORDING TO THE IRRADIATION TIME (MSEC) 68

FIGURE S91. HALOPERIDOL FRAGMENTS INTENSITIES ACCORDING TO THE COLLISION ENERGY (EV) 68
FIGURE S92. RESIDUAL HALOPERIDOL PRECURSOR INTENSITY ACCORDING TO THE IRRADIATION TIME (MSEC) 69
FIGURE S93. RESIDUAL HALOPERIDOL PRECURSOR INTENSITY ACCORDING TO THE COLLISION ENERGY (EV) 69
FIGURE S94. DESMETHYL BOSENTAN FRAGMENTS INTENSITIES ACCORDING TO THE IRRADIATION TIME (MSEC) 70
FIGURE S95. DESMETHYL BOSENTAN FRAGMENTS INTENSITIES ACCORDING TO THE COLLISION ENERGY (EV) 70
FIGURE S96. RESIDUAL DESMETHYL BOSENTAN PRECURSOR INTENSITY ACCORDING TO THE IRRADIATION TIME (MSEC) 71
FIGURE S97. RESIDUAL DESMETHYL BOSENTAN PRECURSOR INTENSITY ACCORDING TO THE COLLISION ENERGY (EV) 71

TABLE S1. LIST OF COMPOUNDS ANALYZED, MRM TRANSITION AND UVPD RESPOND. 7
TABLE S2. TRANSITIONS MRM CID PARAMETERS FOR THE BOSENTAN AND ONE OF ITS METABOLITE QUANTIFICATION METHOD APPLY TO PLASMA SAMPLE. 72

Table S1. List of compounds analyzed, MRM transitions and UVPD respond.

N°	ID	Formula	Q1	Q3	CE (V)	UVPD respond
1	7-Aminoclonazepam	C15H12ClN3O	286.1	222.2	35	yes
2	Acebutolol	C18H28N2O4	337.0	116.0	30	yes
3	Acetamiprid	C10H11ClN4	223.1	126.0	27	yes
4	Alprazolam	C17H13ClN4	309.1	281.3	41	yes
5	Amitriptyline	C20H23N	278.1	91.1	41	yes
6	Angiotensin I 3+	C62H89N17O14	433.1	110.1	30	yes
7	Angiotensin II 2+	C50H71N13O12	523.9	263.2	35	yes
8	Azithromycin	C38H72N2O12	749.5	591.4	35	yes
9	Bendroflumethiazide	C15H14F3N3O4S2	421.8	105.0	25	yes
10	Bentazon	C10H12N2O3S	241.0	198.8	16	yes
11	Benzoylecgonine	C16H19NO4	290.1	168.1	20	yes
12	Bromocriptine	C32H40BrN5O5	654.1	346.1	39	yes
13	Bufuralol	C16H23NO2	256.2	188.1	23	yes
14	Buprenorphine	C29H41NO4	468.3	396.2	50	no
15	Buscopan	C21H30NO4	360.1	194.1	34	weak
16	Buspirone	C21H31N5O2	386.3	122.1	50	yes
17	Caffeine	C8H10N4O2	195.1	138.1	20	yes
18	Carbamazepine	C15H12N2O	237.1	194.1	28	yes
19	Carisoprodol	C12H24N2O4	261.1	176.2	12	yes
20	Chloramphenicol	C11H12Cl2N2O5	323.0	275.0	30	yes
21	Chlorprothixene	C18H18ClNS	316.1	231.1	35	yes
22	Chlorthalidone	C14H11ClN2O4S	338.9	322.0	16	yes
23	Clenbuterol	C12H18Cl2N2O	277.1	203.1	20	yes
24	Clonazepam	C15H10ClN3O3	316.1	270.1	35	yes
25	Cocaine	C17H21NO4	304.1	182.1	20	yes
26	Desipramine	C18H22N2	267.2	72.1	20	yes
27	Diazepam	C16H13ClN2O	285.1	193.1	35	yes
28	Diphenhydramine	C17H21NO	262.0	167.1	20	N/A
29	Epitestosterone	C19H28O2	289.1	97.0	29	yes
30	Erythromycin	C37H67NO13	734.5	158.1	43	no
31	Etodolac	C17H21NO3	288.0	172.2	17	yes
32	Fentanyl	C22H28N2O	337.2	188.1	31	no
33	Flusilazole	C16H15F2N3Si	316.1	247.0	30	yes
34	Glycyrrhizic Acid	C42H62O16	823.4	453.3	38	yes
35	Haloperidol	C21H23ClFNO2	376.1	165.1	35	yes
36	Ibuprofen	C13H18O2	207.1	161.1	20	yes
37	Imidacloprid	C9H10ClN5O2	256.2	209.1	25	yes
38	Indoprofen	C17H15NO3	282.1	236.1	35	yes
39	Ketoconazole	C26H28Cl2N4O4	531.1	489.0	45	yes
40	Ketoprofen	C16H14O3	255.1	209.2	20	yes
41	Ketorolac	C15H13NO3	256.1	105.1	35	yes
42	Lacosamide	C13H18N2O3	251.1	108.1	13	yes

43	Lamotrigine	C9H7Cl2N5	256.0	211.0	35	yes
44	Loperamide	C29H33ClN2O2	477.2	266.4	20	yes
45	Lovastatin	C24H36O5	405.2	285.2	16	weak
46	L-tryptophan	C11H12N2O2	205.1	188.1	20	yes
47	Melatonin	C13H16N2O2	233.1	174.1	20	yes
48	Meprobamate	C9H18N2O4	219.1	158.1	15	yes
49	Methadone	C21H27NO	310.2	265.2	20	yes
50	Midazolam	C18H13ClFN3	326.1	291.1	45	yes
51	Minoxidil	C9H15N5O	210.1	193.1	20	yes
52	Naproxen	C14H14O3	231.1	185.1	30	yes
53	Nirvanol	C11H12N2O2	205.1	134.1	35	yes
54	Norbuprenorphine	C25H35NO4	414.3	83.1	60	yes
55	Normeperidine	C14H19NO2	234.1	160.0	30	yes
56	Norsertraline HCL	C16H15Cl2N	275.1	159.1	20	yes
69	Nortriptyline	C19H21N	264.0	155.0	25	yes
57	Oxazepam	C15H11ClN2O2	287.1	241.2	35	yes
58	Pamaquine	C19H29N3O	316.2	243.1	30	yes
59	Perphenazine	C21H26ClN3OS	404.2	171.2	35	yes
60	Phenacetin	C10H13NO2	180	110.1	28	yes
61	Phenyltoloxamine	C17H21NO	256.2	72.0	35	weak
62	Prazosin	C19H21N5O4	383.8	247.1	40	yes
63	Prednisolone	C21H28O5	361.1	147.1	34	yes
64	Progesterone	C21H30O2	315.2	109.1	35	yes
65	Propafenone	C21H27NO3	342.2	116.1	35	weak
66	Propoxyphene	C22H29NO2	340.1	266.1	20	weak
67	Propranolol	C16H21NO2	260.0	116.1	25	yes
68	Protriptyline	C19H21N	264.0	91.0	25	yes
70	Reserpine	C33H40N2O9	609.2	195.0	47	yes
71	Rifampicin	C43H58N4O12	823.4	95.1	50	yes
72	Ritalinic Acid	C13H17NO2	220.1	84.1	25	weak
73	Ritonavir	C37H48N6O5S2	720.8	296.2	27	yes
74	Secobarbital	C12H18N2O3	239.1	196.0	30	yes
75	Sulfadiazine	C10H10N4O2S	251.1	92.1	35	yes
76	Sulfamerazine	C11H12N4O2S	265.0	156.0	23	yes
77	Sulfamethazine	C12H14N4O2S	278.9	186.0	24	yes
78	Tamoxifen	C26H29NO	372.1	72.1	48	weak
79	Tapentadol	C14H23NO	222.1	107.0	35	no
80	Taurocholic Acid	C26H45NO7S	516.3	462.2	30	yes
81	Temazepam	C16H13ClN2O2	302.1	255.2	20	yes
82	Terfenadine	C32H41NO2	472.3	436.3	35	yes
83	Testosterone	C19H28O2	289.2	109.1	40	yes
84	Tolbutamide	C12H18N2O3S	271.1	155.0	20	yes
85	Tramadol	C16H25NO2	264.2	58.0	20	yes
86	Tylosin	C46H77NO17	916.0	174.1	48	yes

87	Urapidil	C20H29N5O3	388.2	205.2	35	yes
88	Verapamil	C27H38N2O4	455.3	165.2	35	yes
89	Warfarin	C19H16O4	309.1	163.1	20	yes
90	Zolpidem	C19H21N3O	308.0	235.2	50	yes

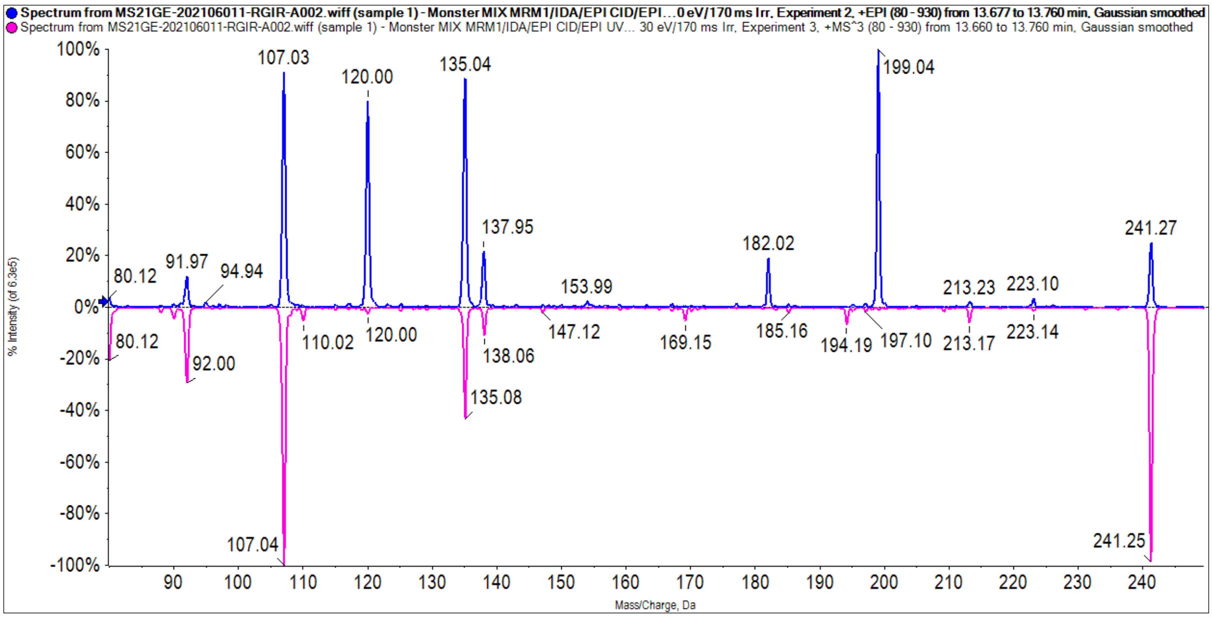

Figure S1. Bentazon CID (upper) and UVPD (lower) fragmentation spectra zoom from 80 to 250 *m/z*.

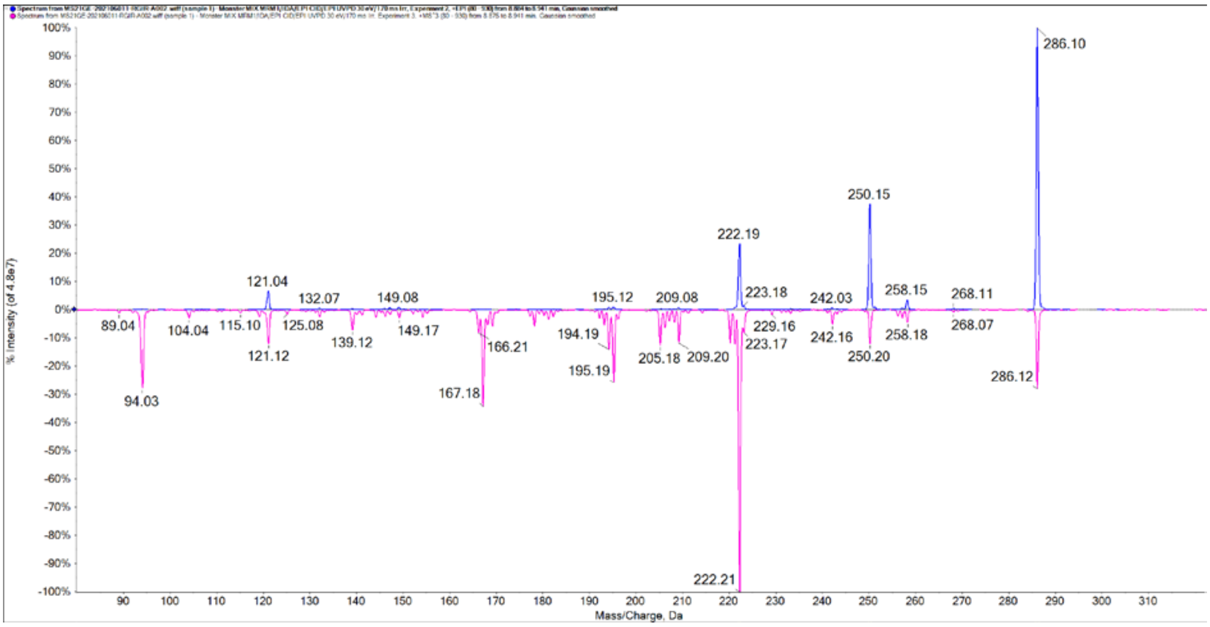

Figure S2. 7-Aminoclonazepam CID (upper) and UVPD (lower) fragmentation spectra zoom from 80 to 320 *m/z.*

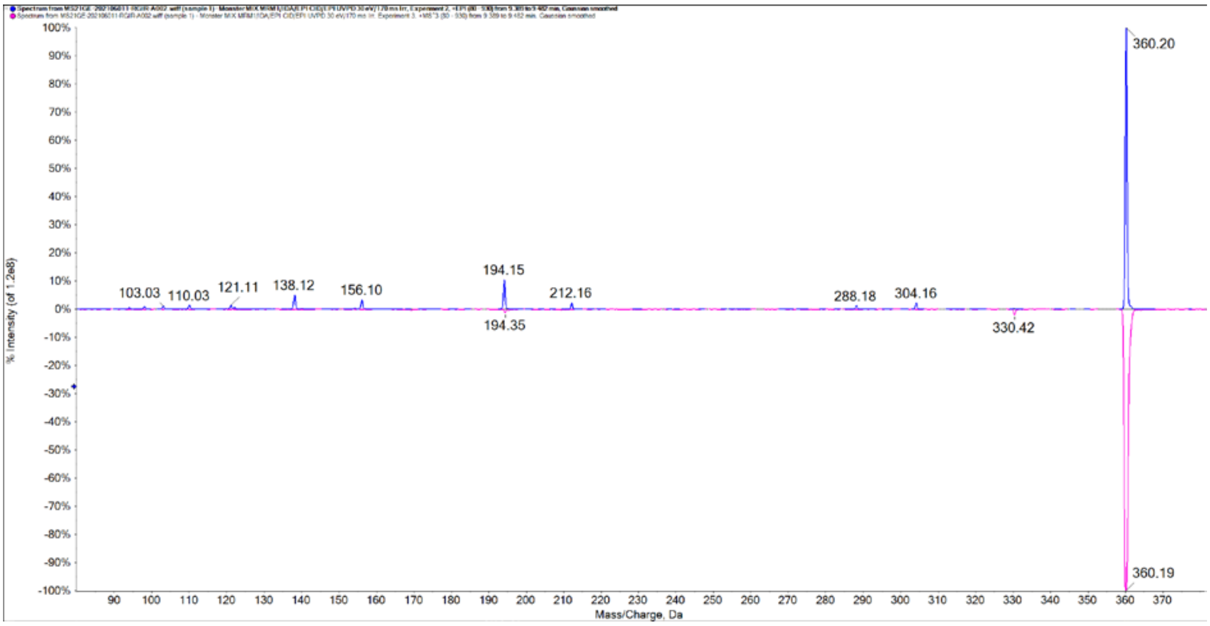

Figure S3. Buscopan CID (upper) and UVPD (lower) fragmentation spectra zoom from 80 to 380 *m/z*.

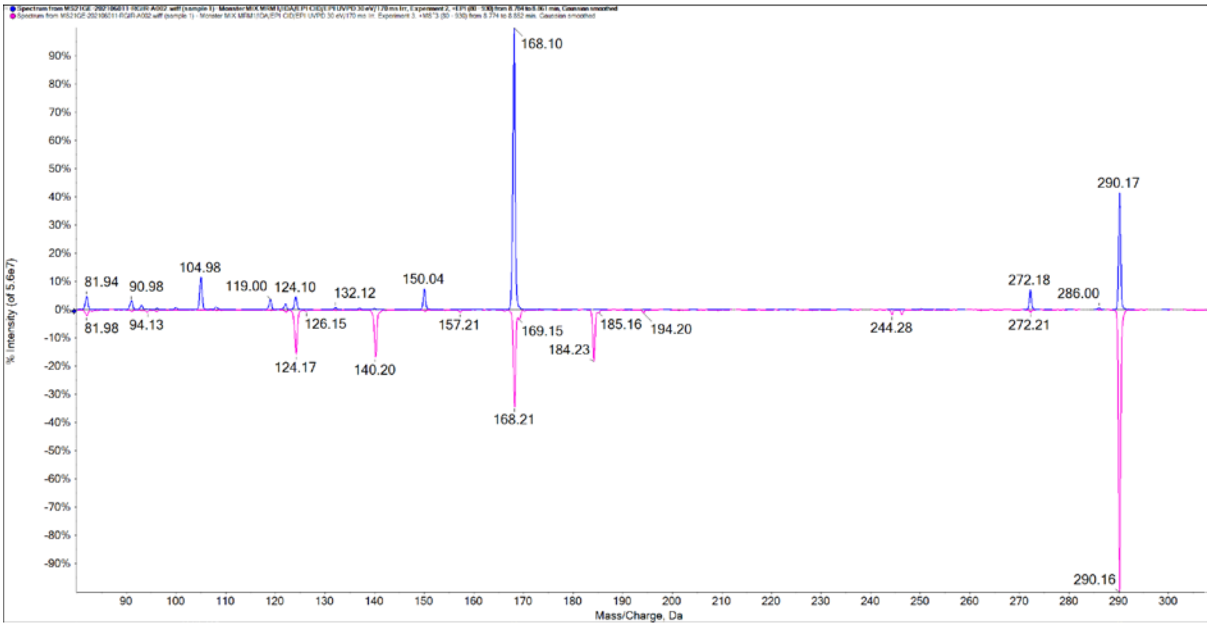

Figure S4. Benzoylecgonine CID (upper) and UVPD (lower) fragmentation spectra zoom from 80 to 310 *m/z*.

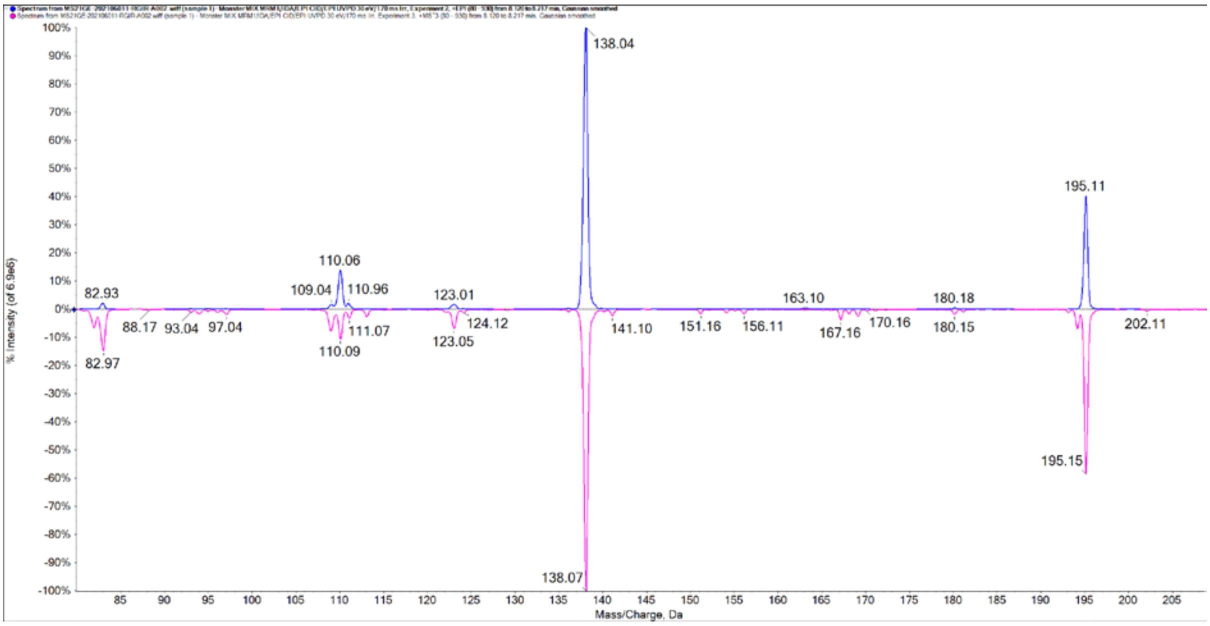

Figure S5. Caffeine CID (upper) and UVPD (lower) fragmentation spectra zoom from 80 to 210 *m/z*.

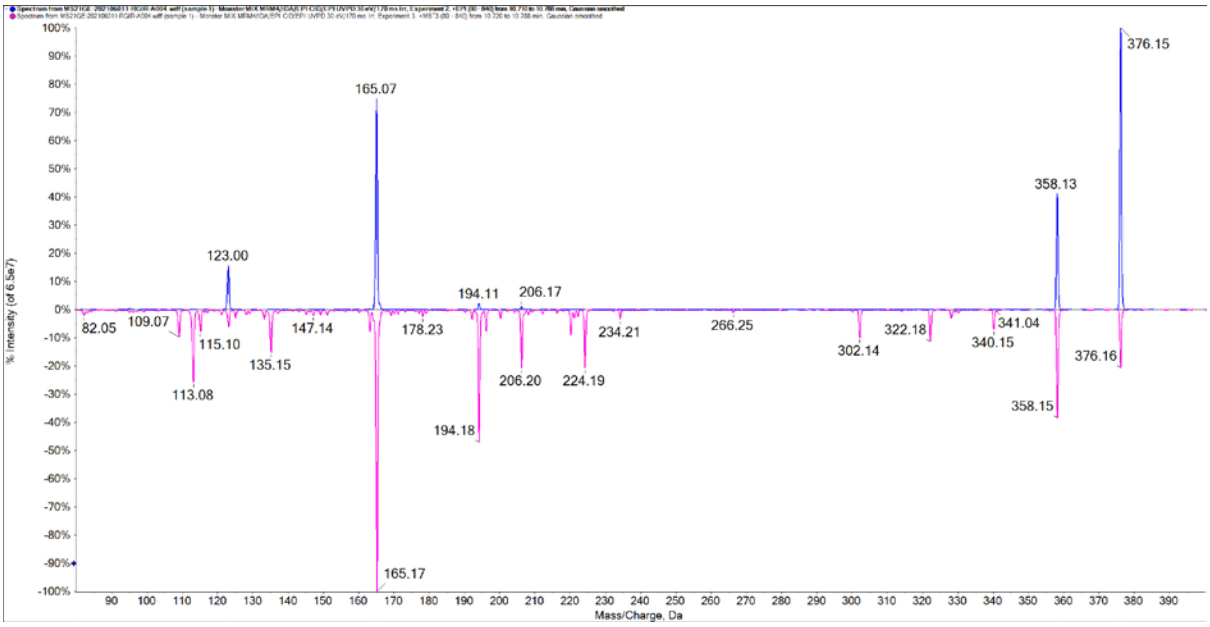

Figure S6. Haloperidol CID (upper) and UVPD (lower) fragmentation spectra zoom from 80 to 400 *m/z*.

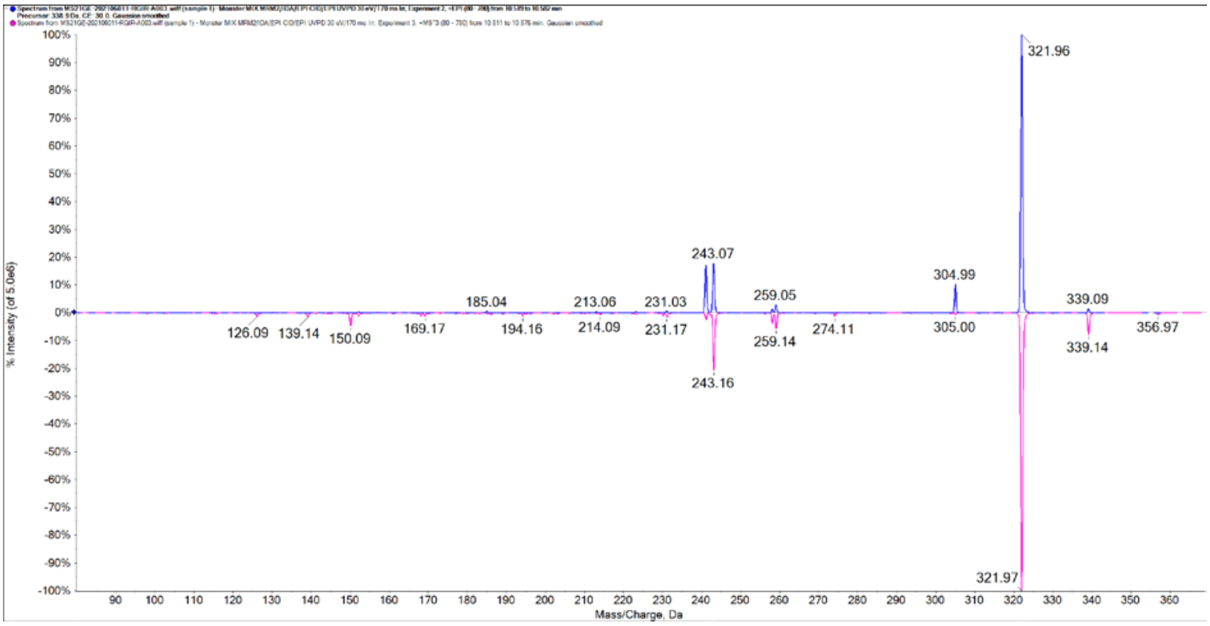

Figure S7. Chlorthalidone CID (upper) and UVPD (lower) fragmentation spectra zoom from 80 to 370 *m/z*.

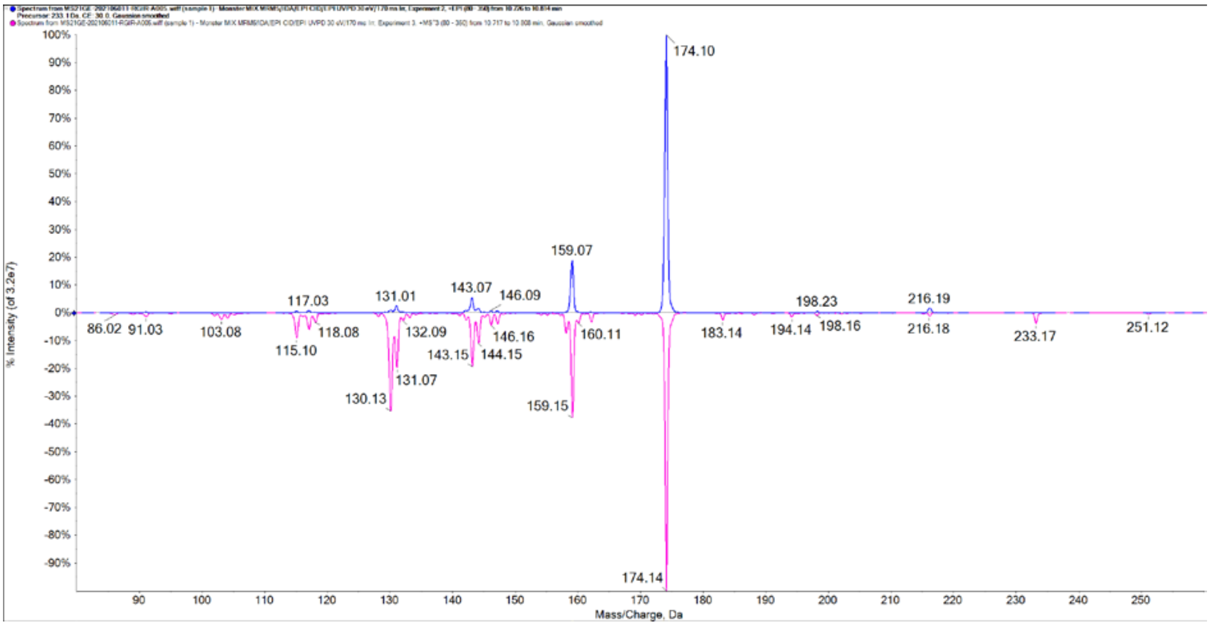

Figure S8. Melatonin CID (upper) and UVPD (lower) fragmentation spectra zoom from 80 to 260 *m/z*.

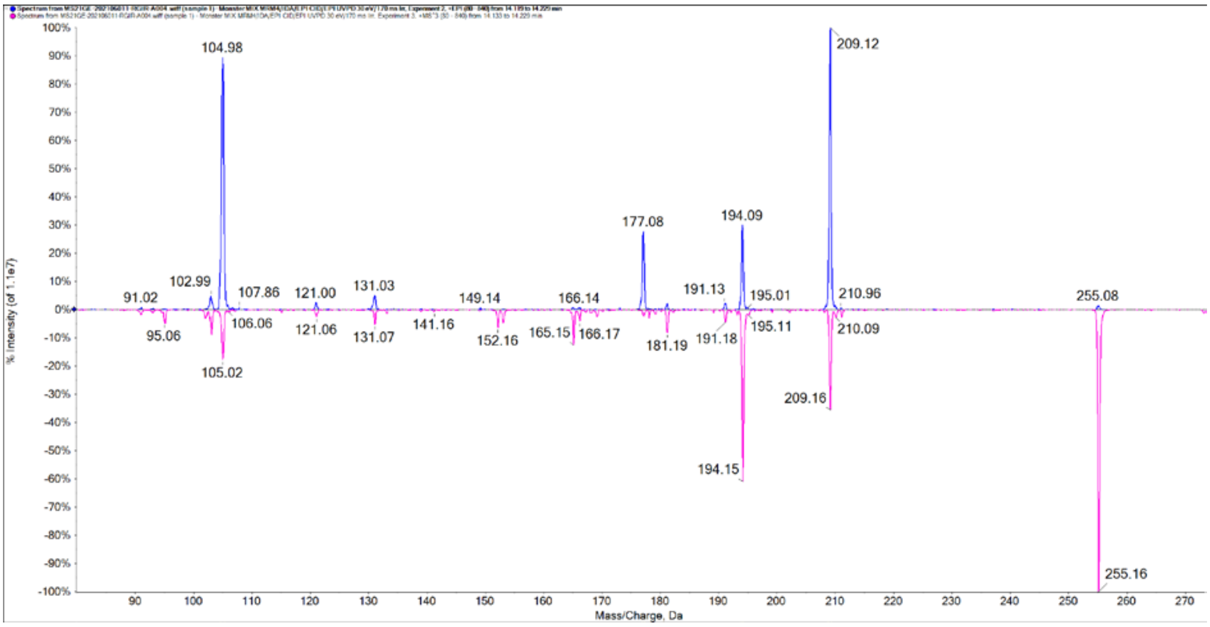

Figure S9. Ketoprofen CID (upper) and UVPD (lower) fragmentation spectra zoom from 80 to 270 *m/z*.

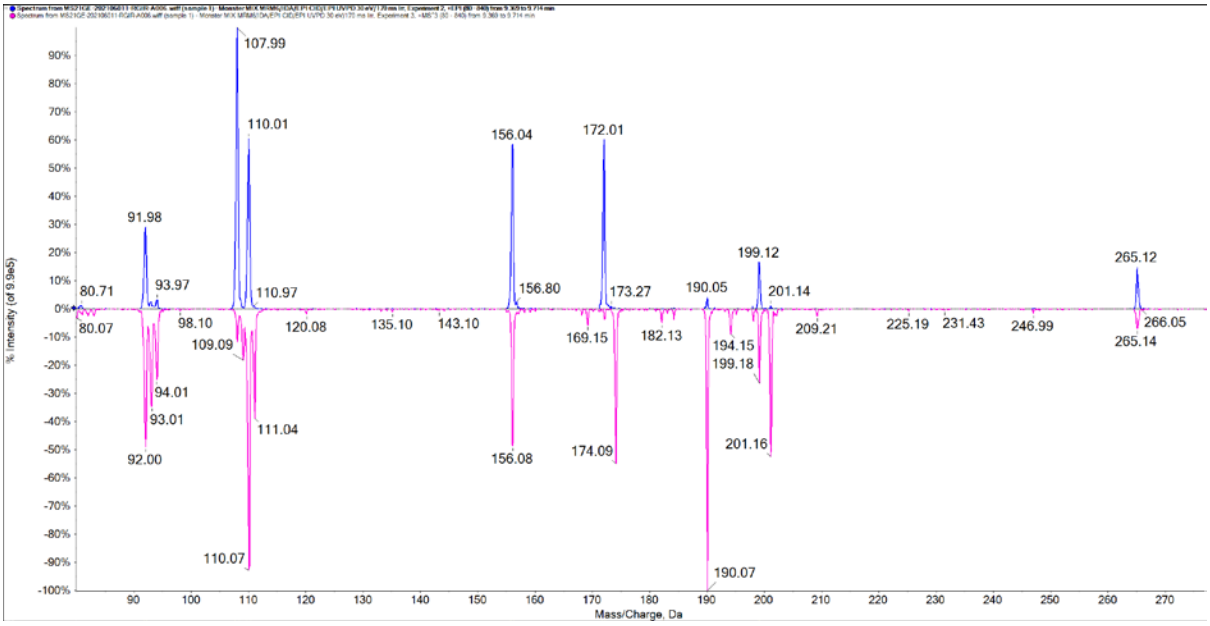

Figure S10. Sulfamerazine CID (upper) and UVPD (lower) fragmentation spectra zoom from 80 to 280 *m/z*.

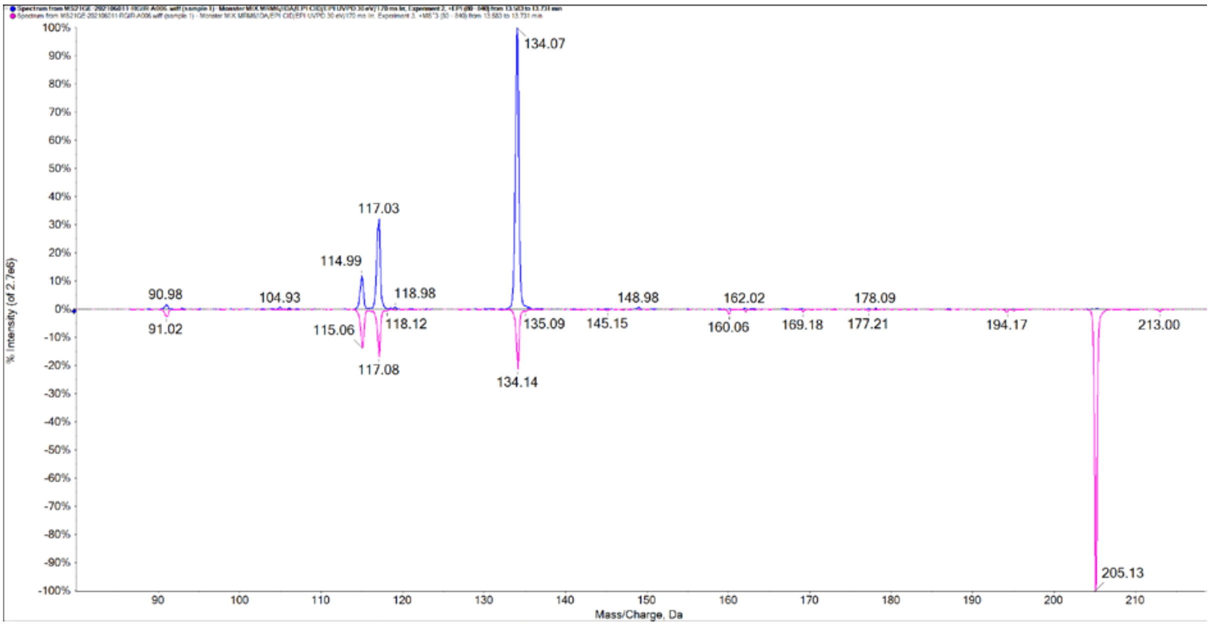

Figure S11. Nirvanol CID (upper) and UVPD (lower) fragmentation spectra zoom from 80 to 220 *m/z*.

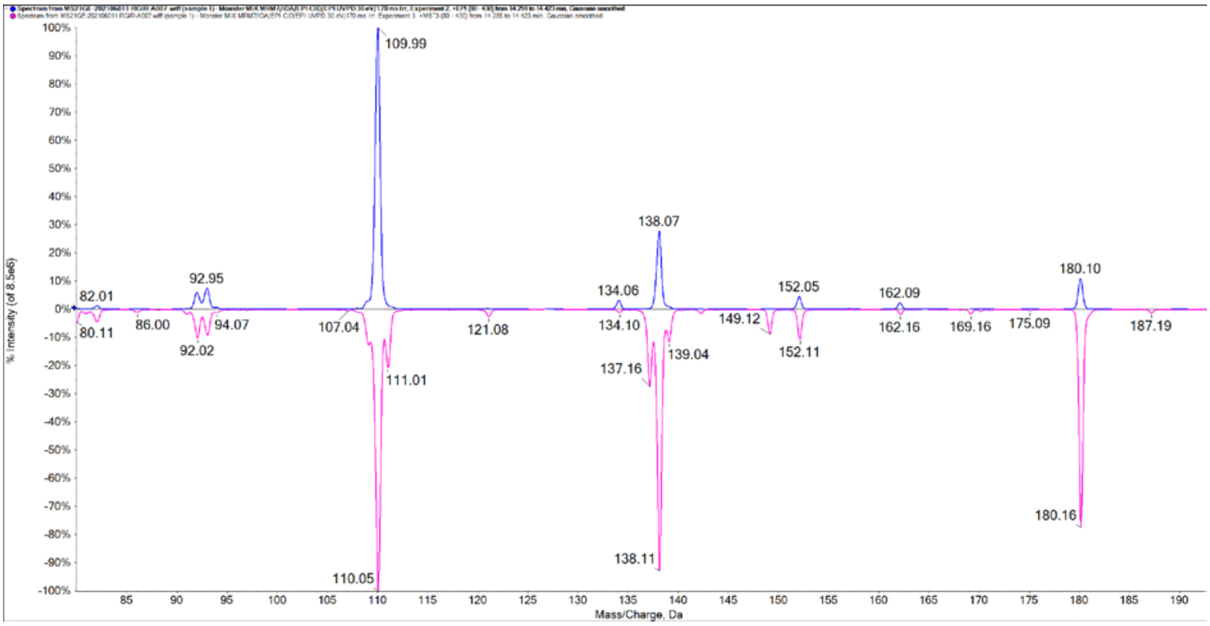

Figure S12. Phenacetin CID (upper) and UVPD (lower) fragmentation spectra zoom from 80 to 195 *m/z*.

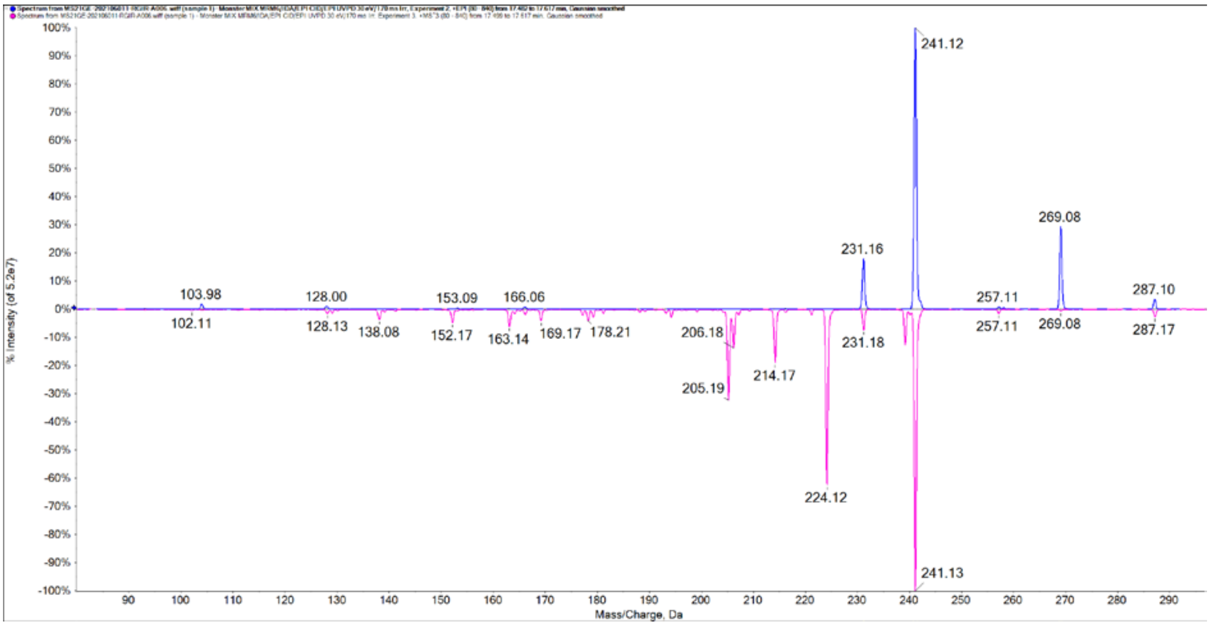

Figure S13. Oxazepam CID (upper) and UVPD (lower) fragmentation spectra zoom from 80 to 300 *m/z*.

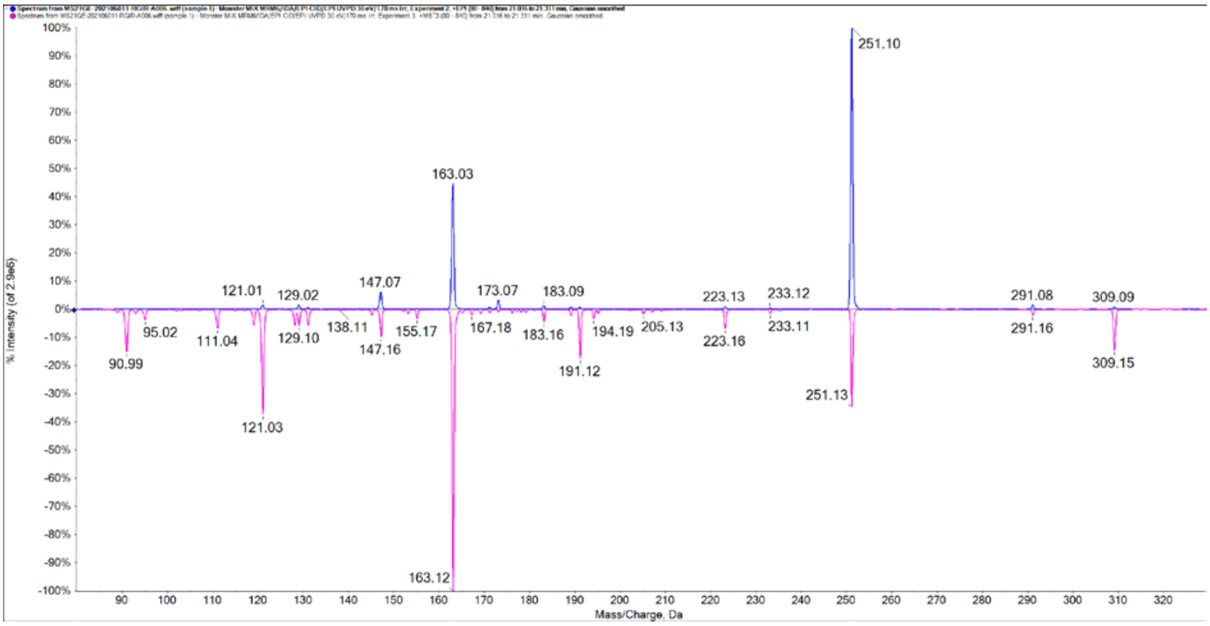

Figure **S14.** Warfarin CID (upper) and UVPD (lower) fragmentation spectra zoom from 80 to 320 *m/z*.

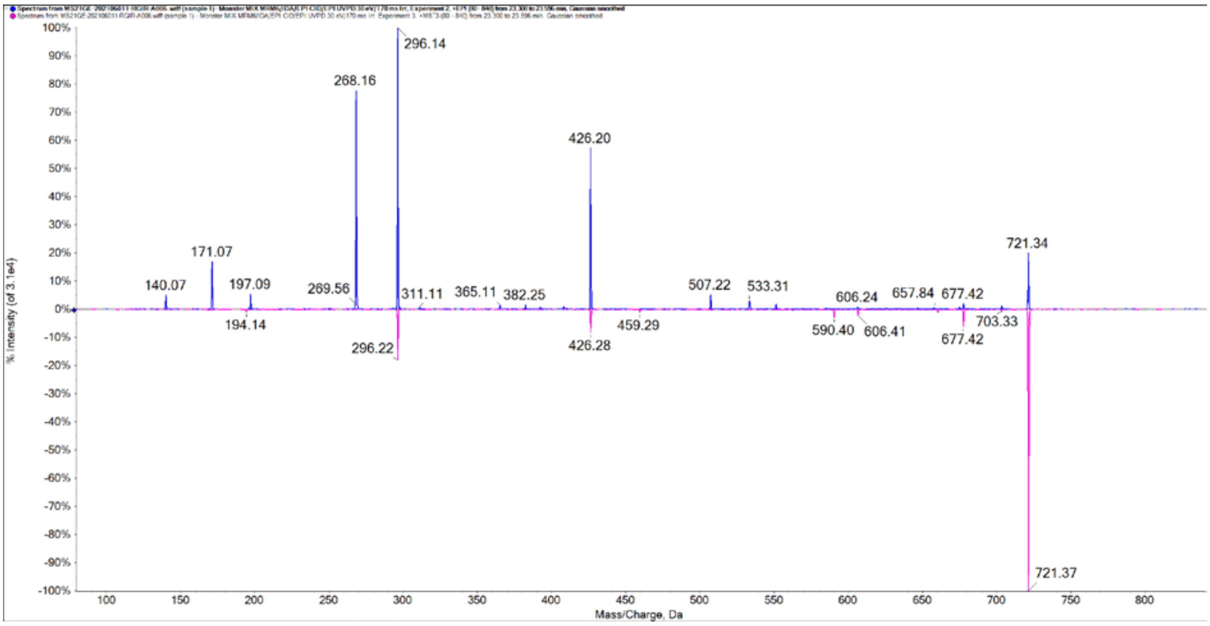

Figure S15. Ritonavir CID (upper) and UVPD (lower) fragmentation spectra zoom from 80 to 750 *m/z*.

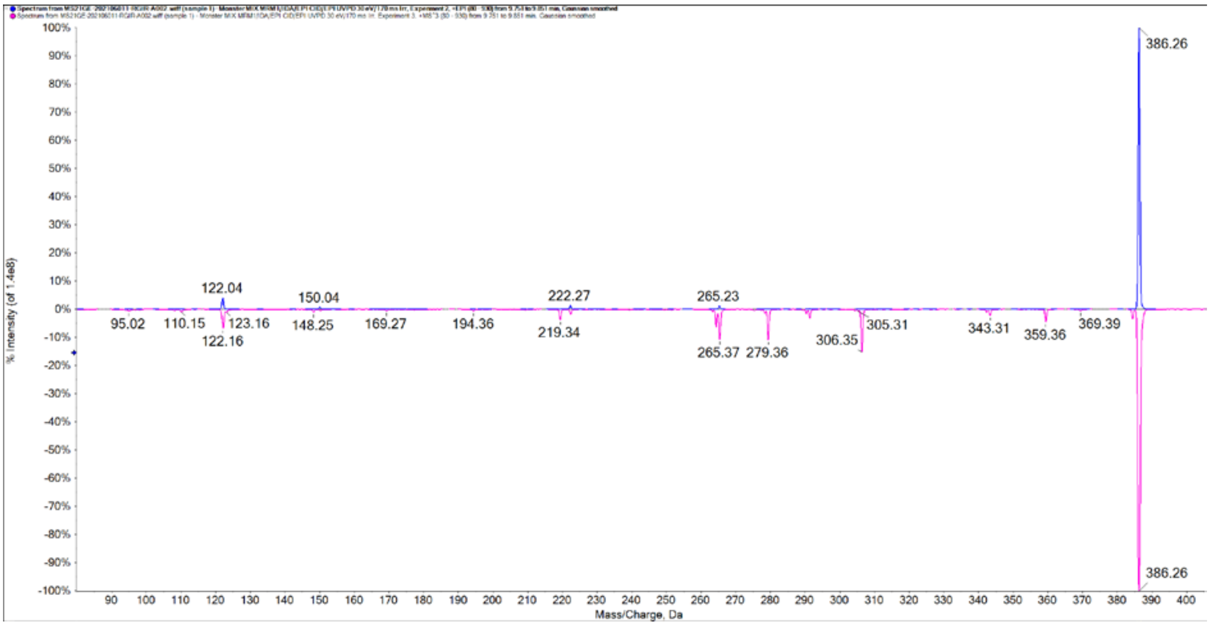

Figure S16. Buspirone CID (upper) and UVPD (lower) fragmentation spectra zoom from 80 to 405 *m/z*.

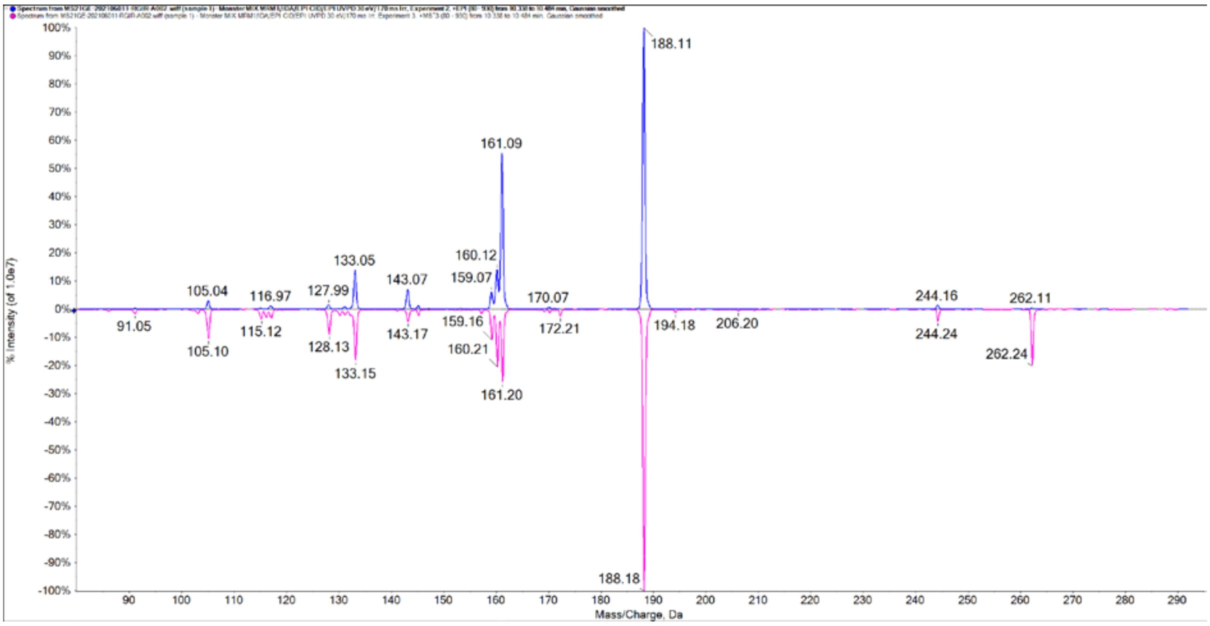

Figure S17. Bufuralol CID (upper) and UVPD (lower) fragmentation spectra zoom from 80 to 295 *m/z*.

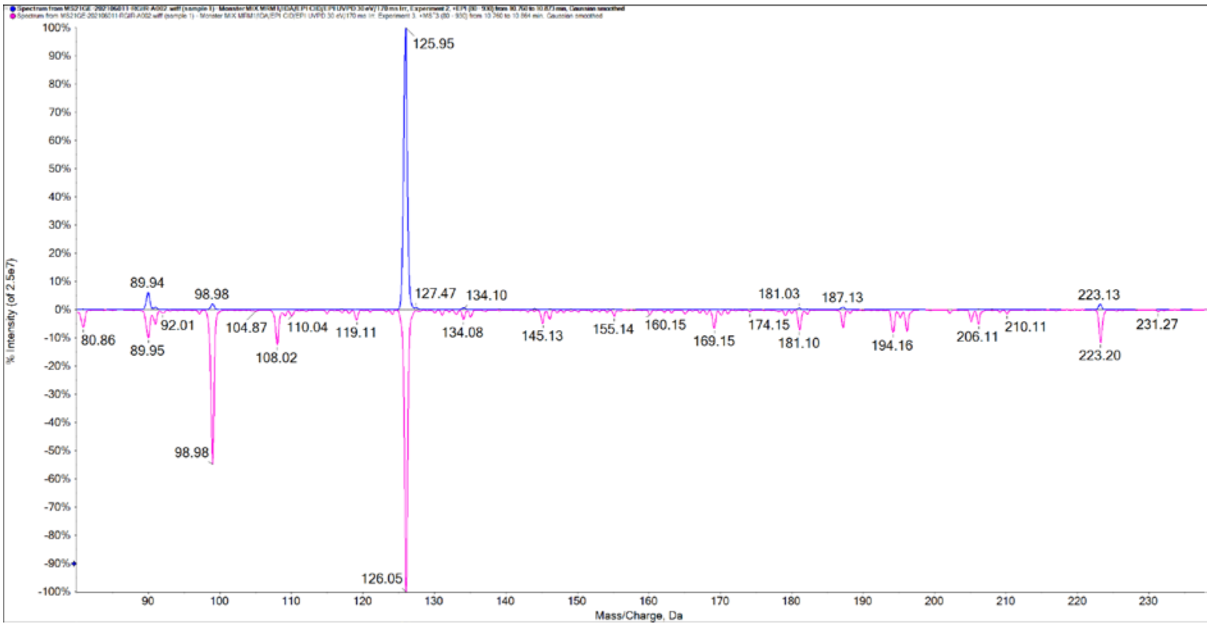

Figure S18. Acetamiprid CID (upper) and UVPD (lower) fragmentation spectra zoom from 80 to 240 *m/z*.

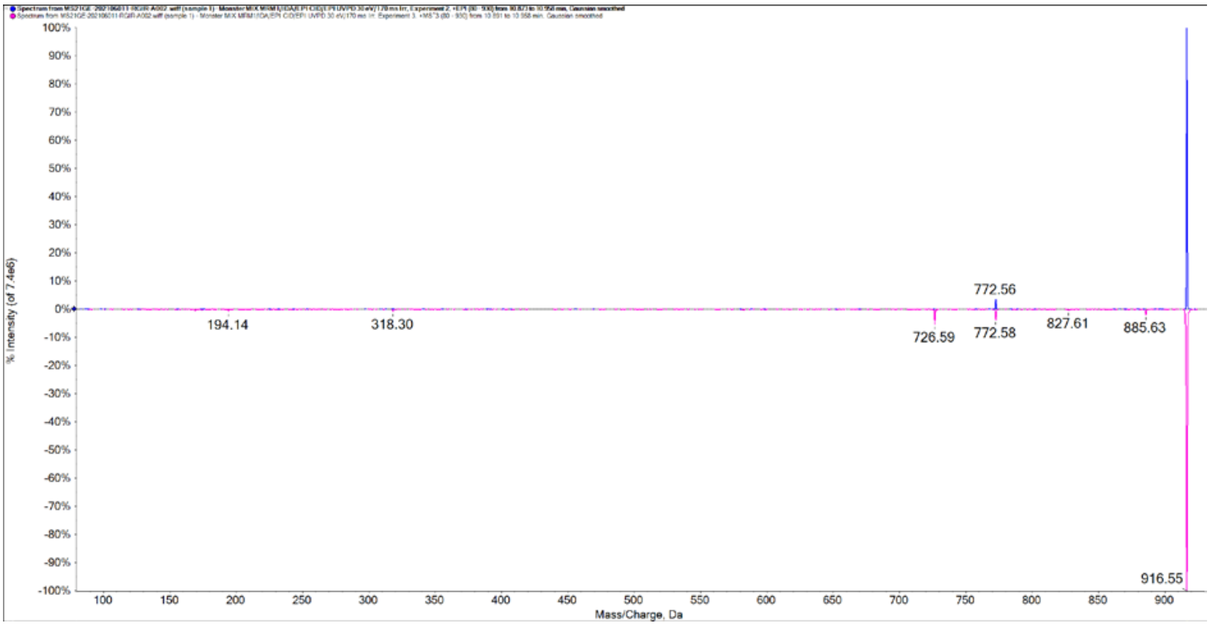

Figure S19. Tylosin CID (upper) and UVPD (lower) fragmentation spectra zoom from 80 to 930 *m/z*.

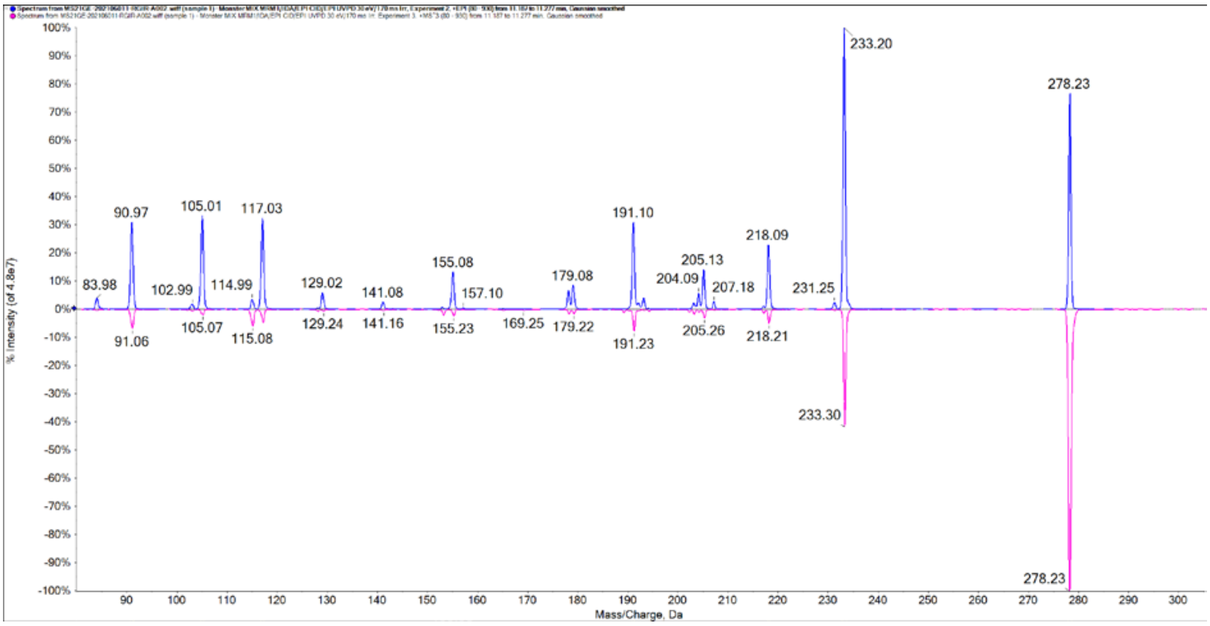

Figure S20. Amitriptyline CID (upper) and UVPD (lower) fragmentation spectra zoom from 80 to 305 *m/z*.

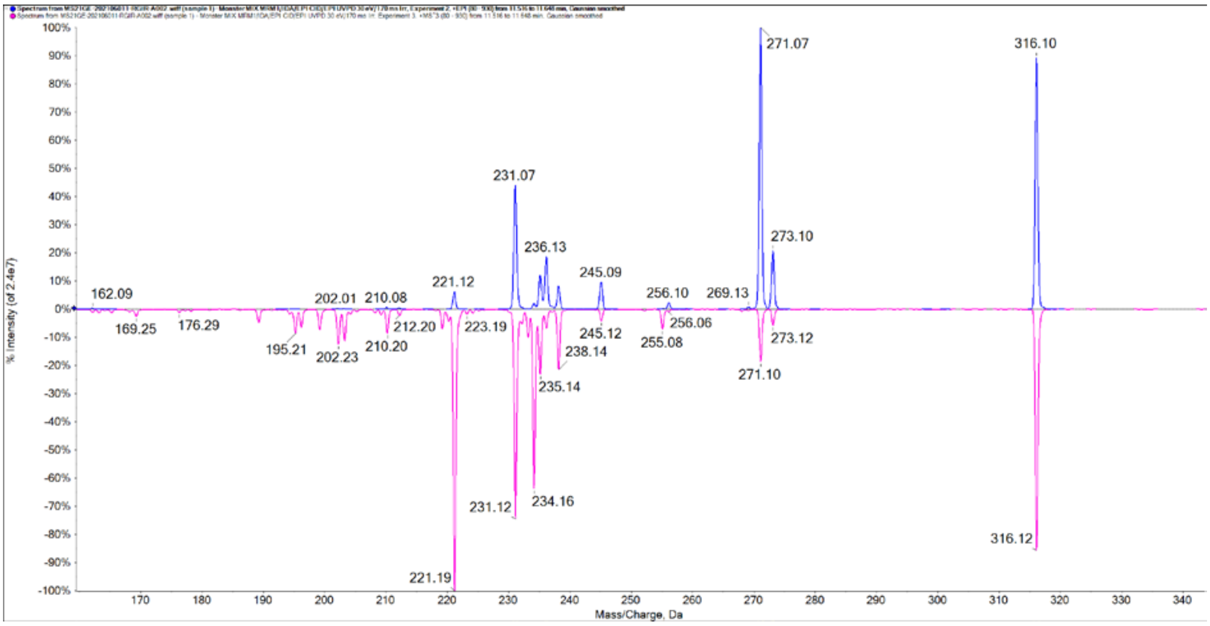

Figure S21. Chlorprothixene CID (upper) and UVPD (lower) fragmentation spectra zoom from 80 to 345 *m/z*.

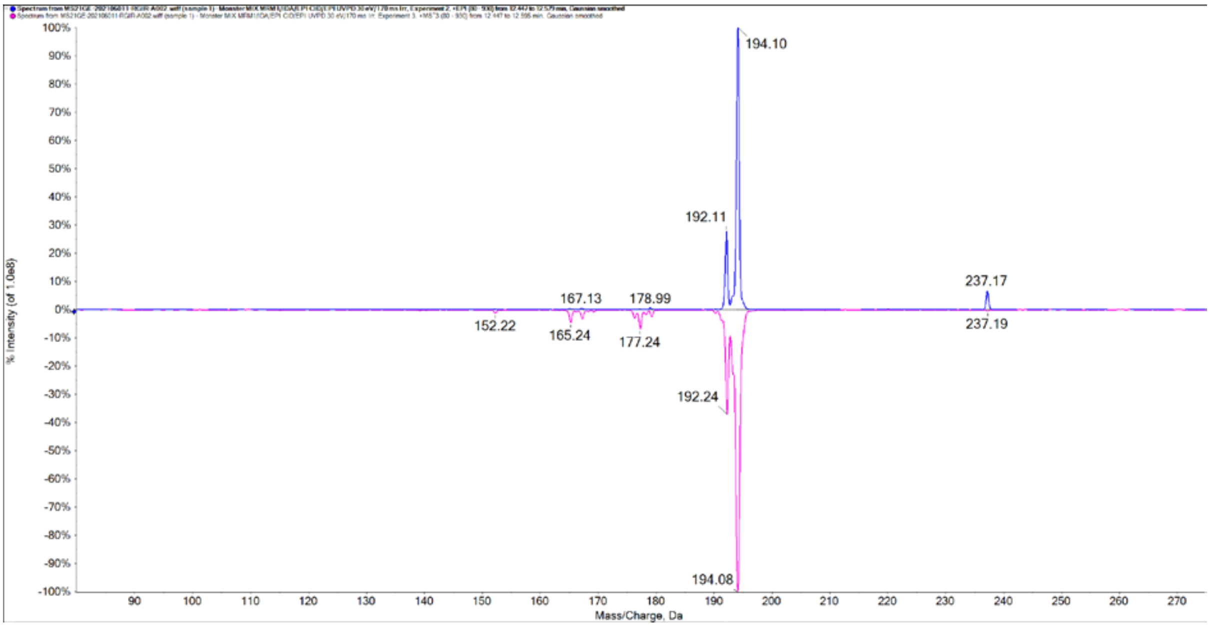

Figure S22. Carbamazepine CID (upper) and UVPD (lower) fragmentation spectra zoom from 80 to 275 *m/z*.

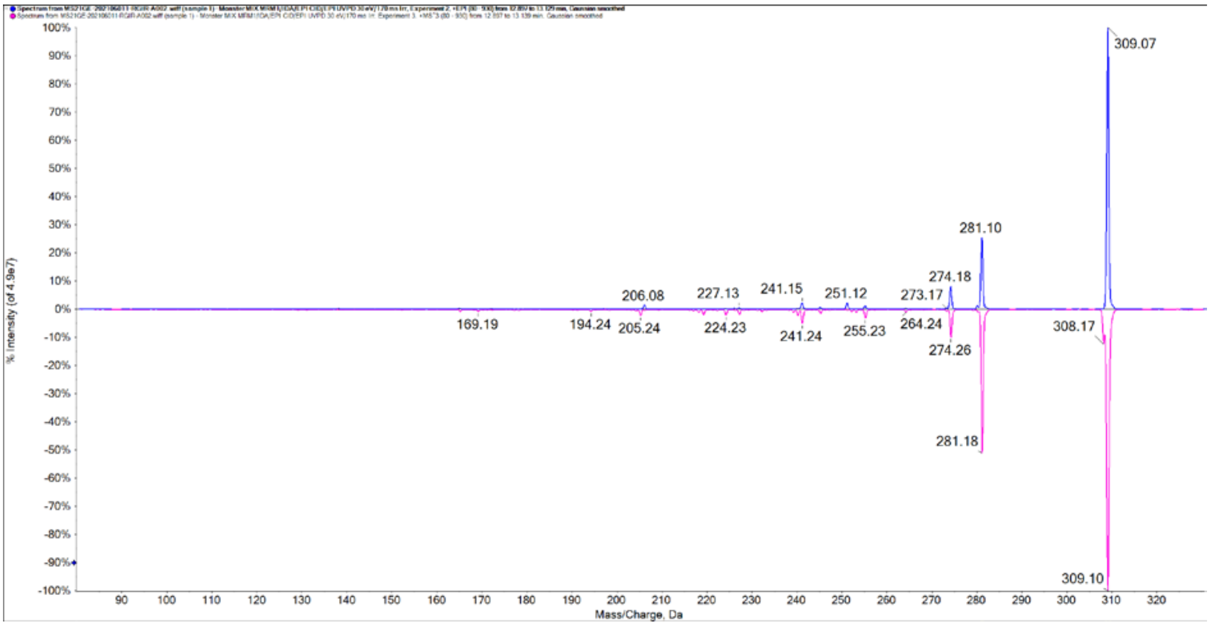

Figure S23. Alprazolam CID (upper) and UVPD (lower) fragmentation spectra zoom from 80 to 325 *m/z*.

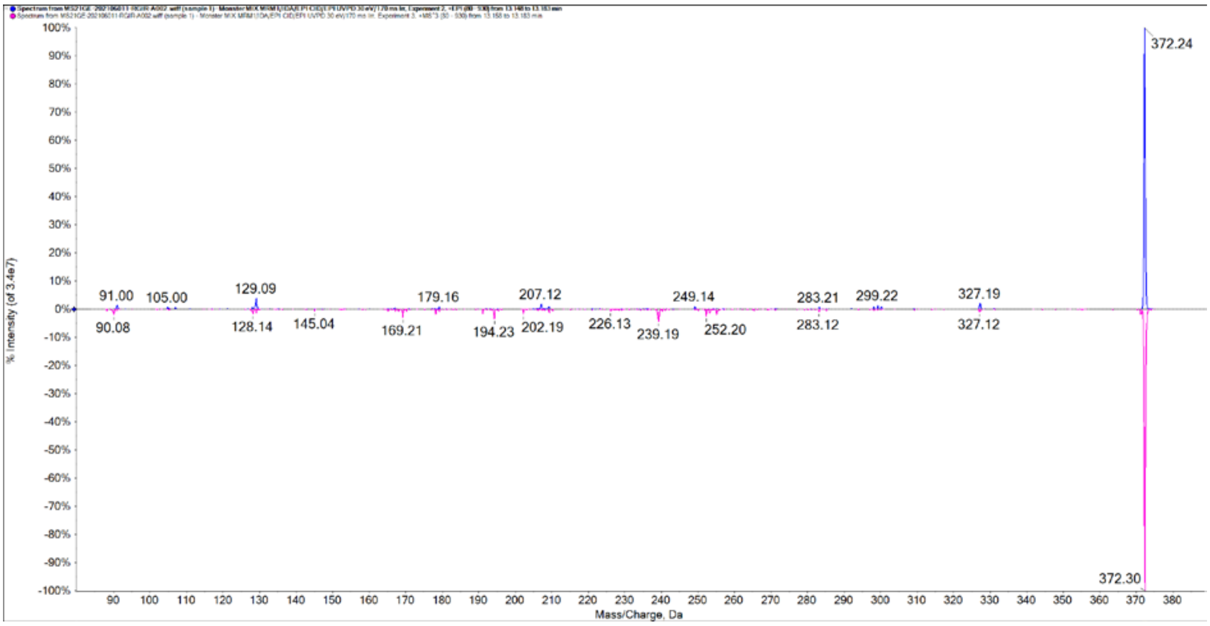

Figure S24. Tamoxifen CID (upper) and UVPD (lower) fragmentation spectra zoom from 80 to 390 *m/z*.

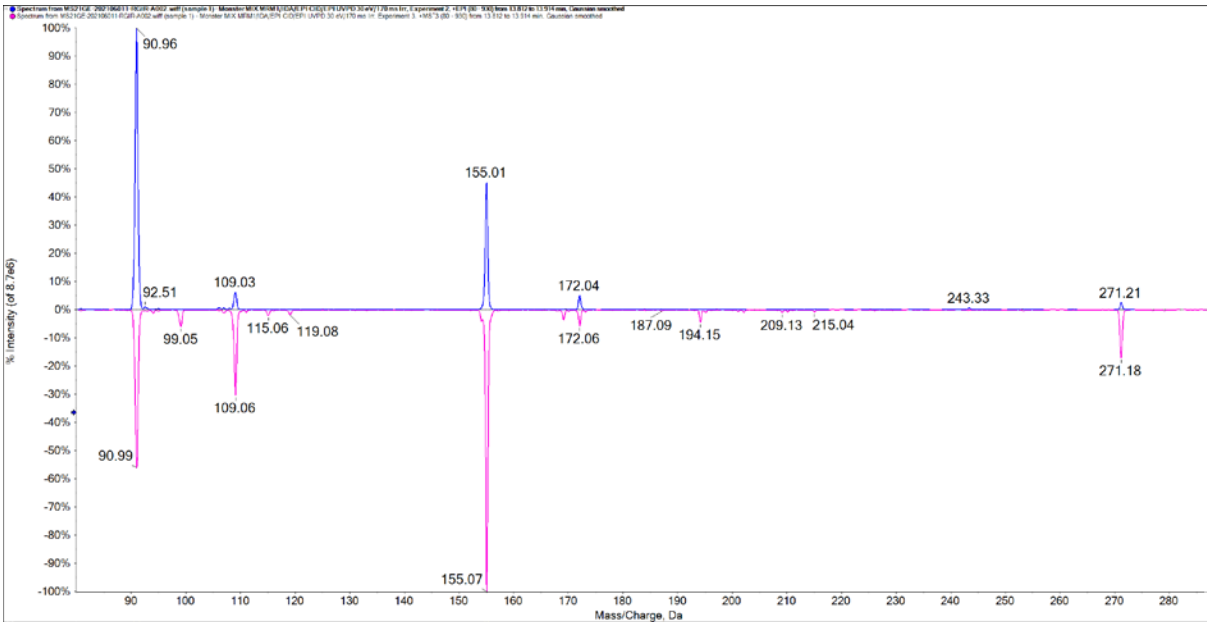

Figure S25. Tolbutamide CID (upper) and UVPD (lower) fragmentation spectra zoom from 80 to 290 *m/z*.

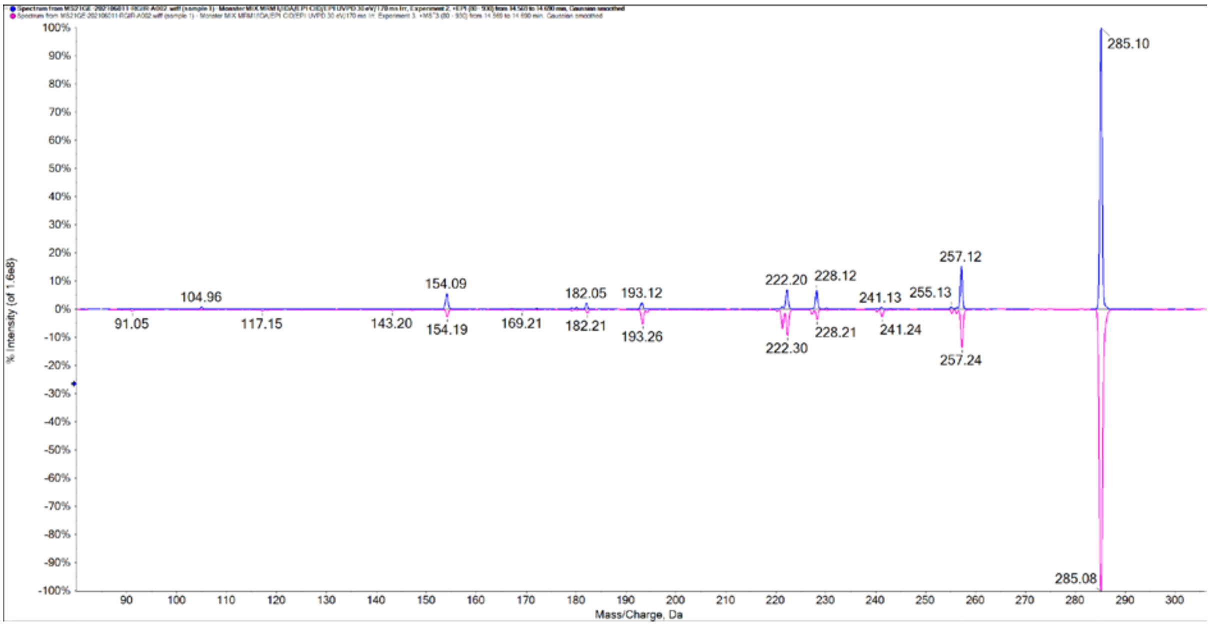

Figure S26. Diazepam CID (upper) and UVPD (lower) fragmentation spectra zoom from 80 to 305 *m/z*.

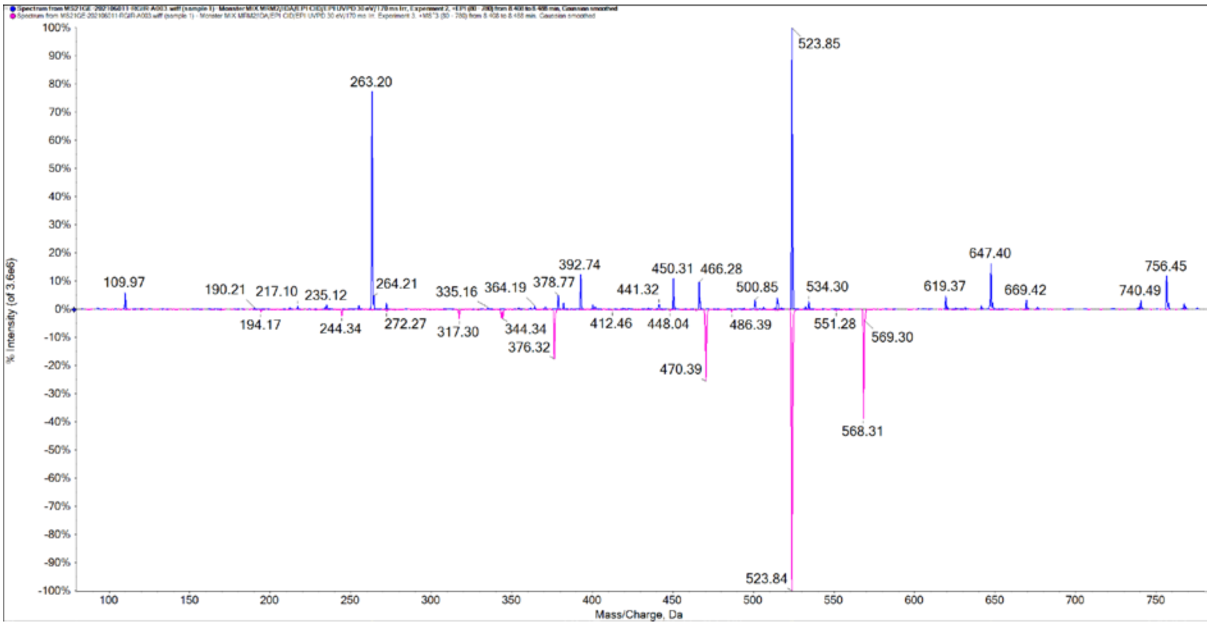

Figure S27. Angiotensin II 2+ CID (upper) and UVPD (lower) fragmentation spectra zoom from 80 to 780 *m/z*.

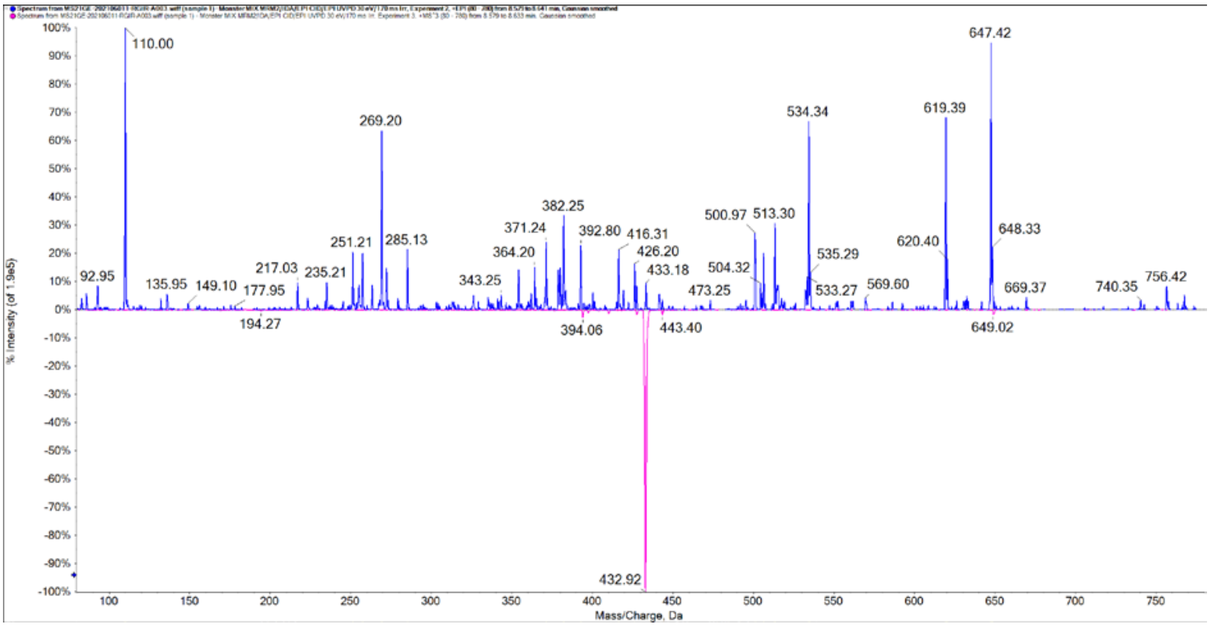

Figure S28. Angiotensin I 3+ CID (upper) and UVPD (lower) fragmentation spectra zoom from 80 to 780 *m/z*.

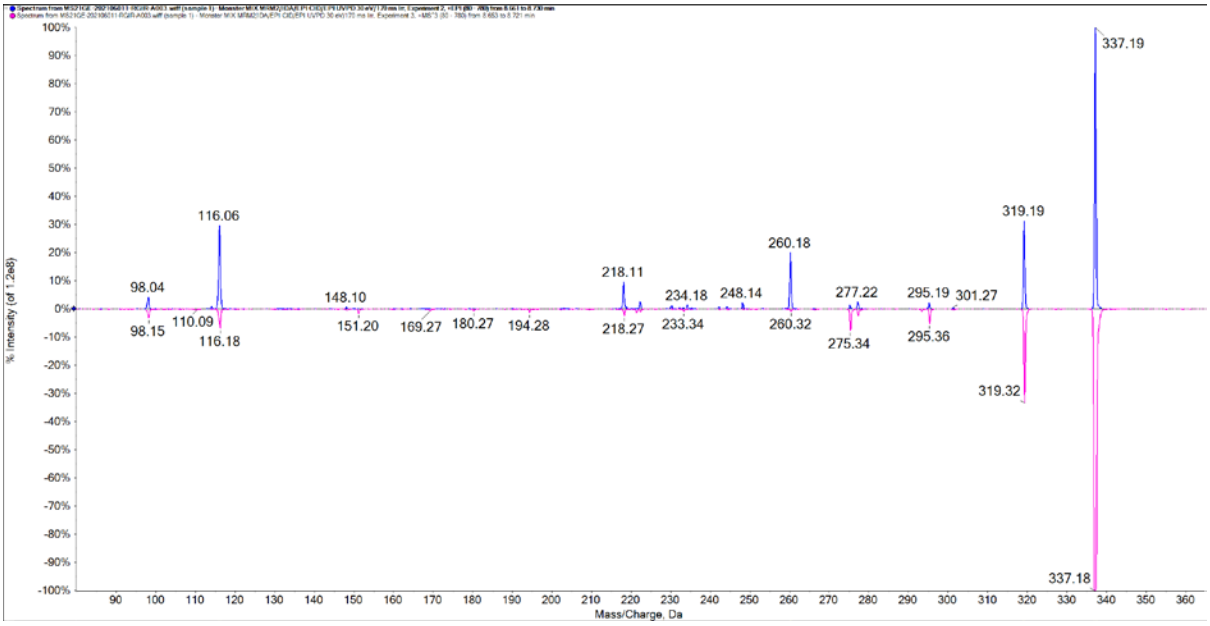

Figure S29. Acebutolol CID (upper) and UVPD (lower) fragmentation spectra zoom from 80 to 360 *m/z*.

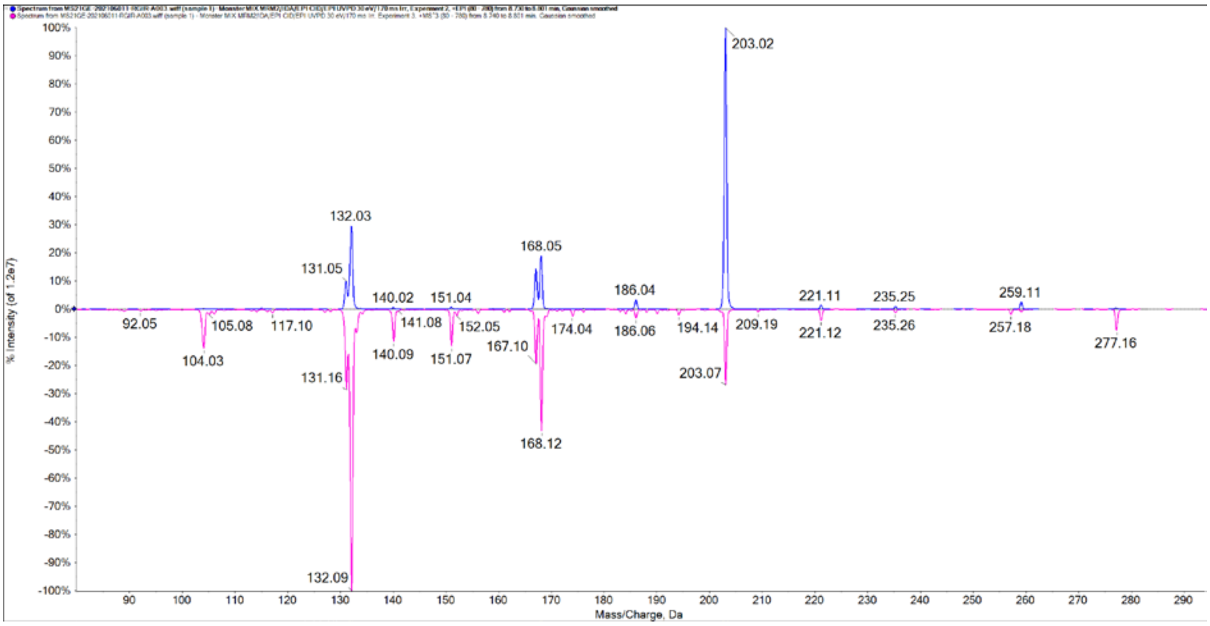

Figure S30. Clenbuterol CID (upper) and UVPD (lower) fragmentation spectra zoom from 80 to 290 *m/z*.

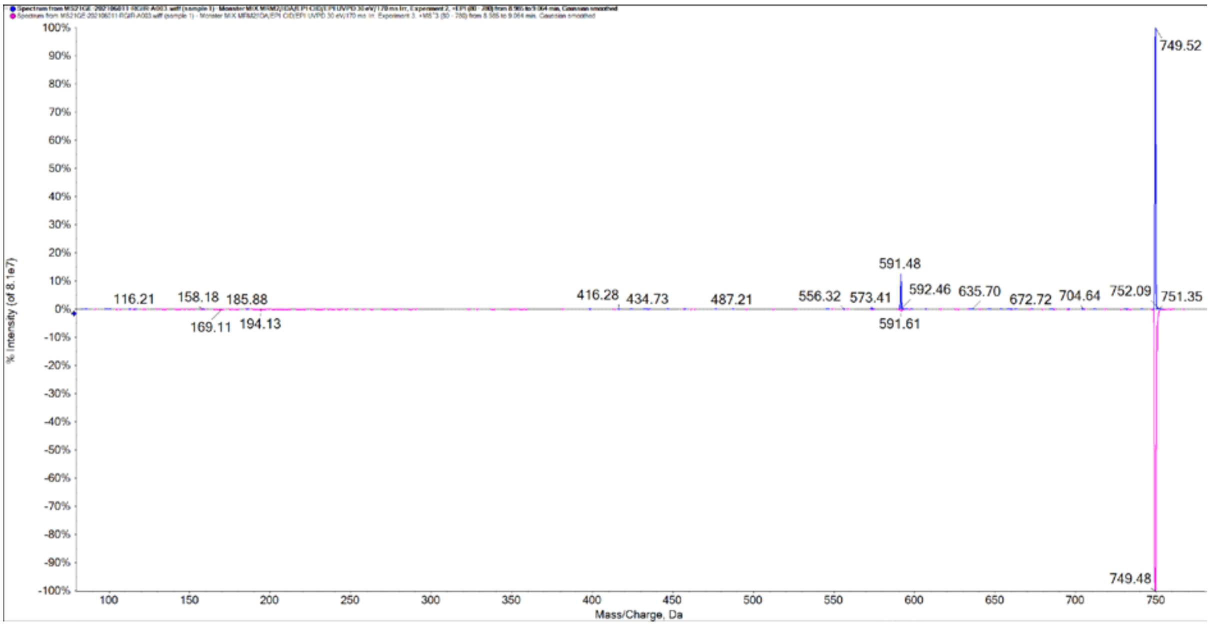

Figure S31. Azithromycin CID (upper) and UVPD (lower) fragmentation spectra zoom from 80 to 780 *m/z*.

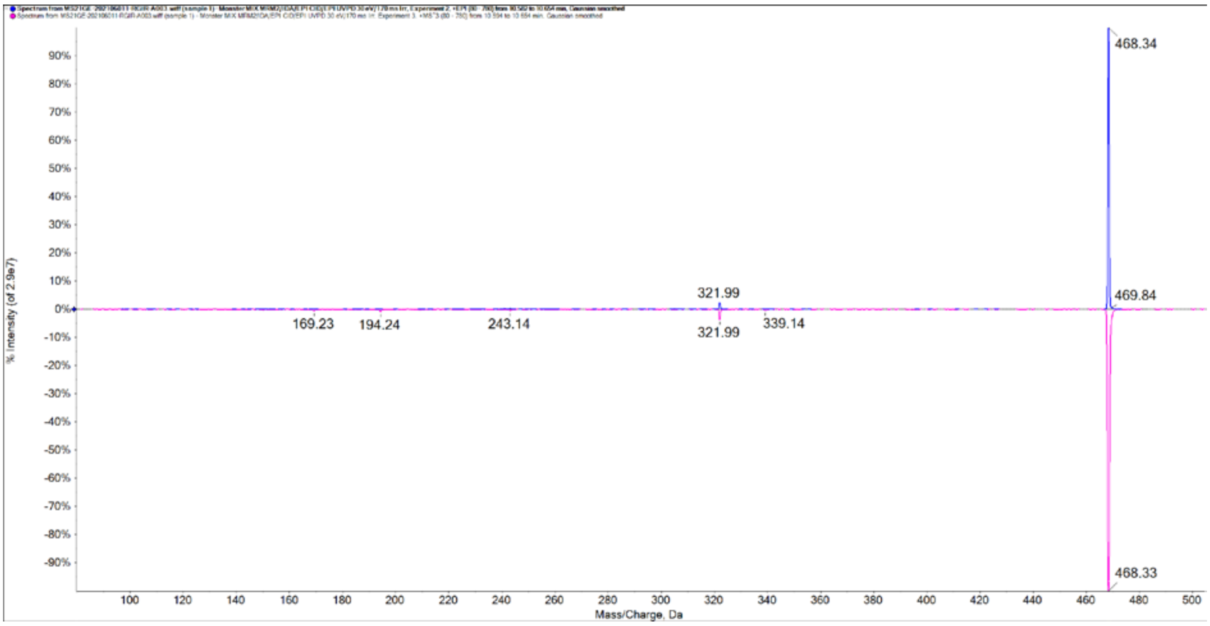

Figure S32. Buprenorphine CID (upper) and UVPD (lower) fragmentation spectra zoom from 80 to 500 *m/z*.

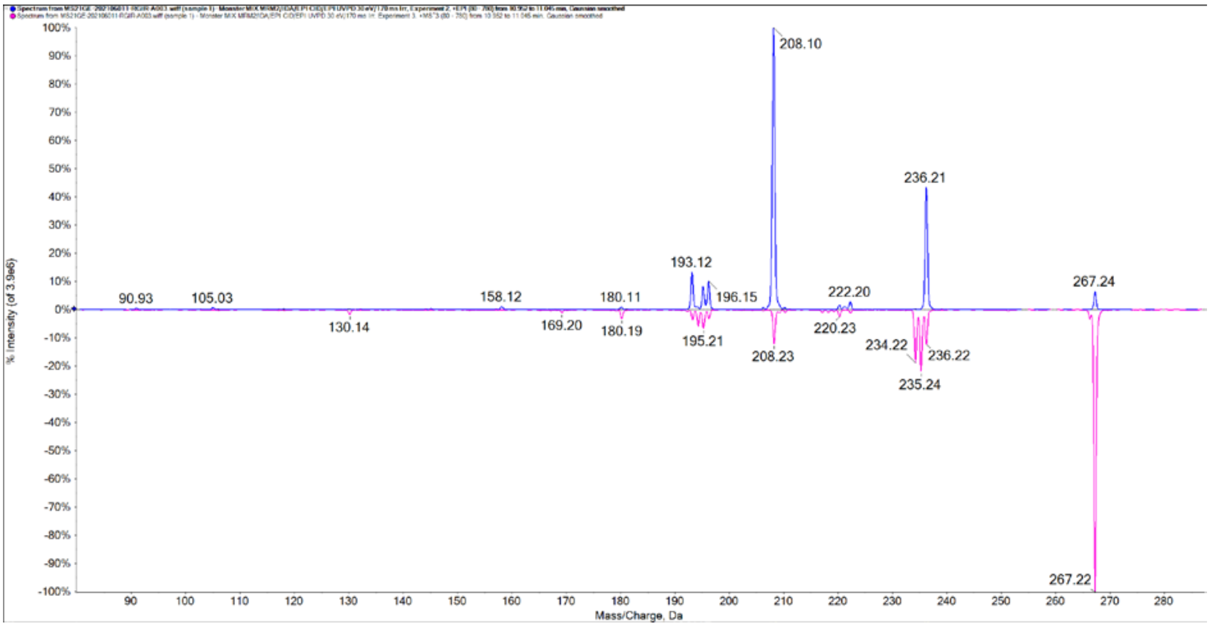

Figure S33. Desipramine CID (upper) and UVPD (lower) fragmentation spectra zoom from 80 to 290 *m/z*.

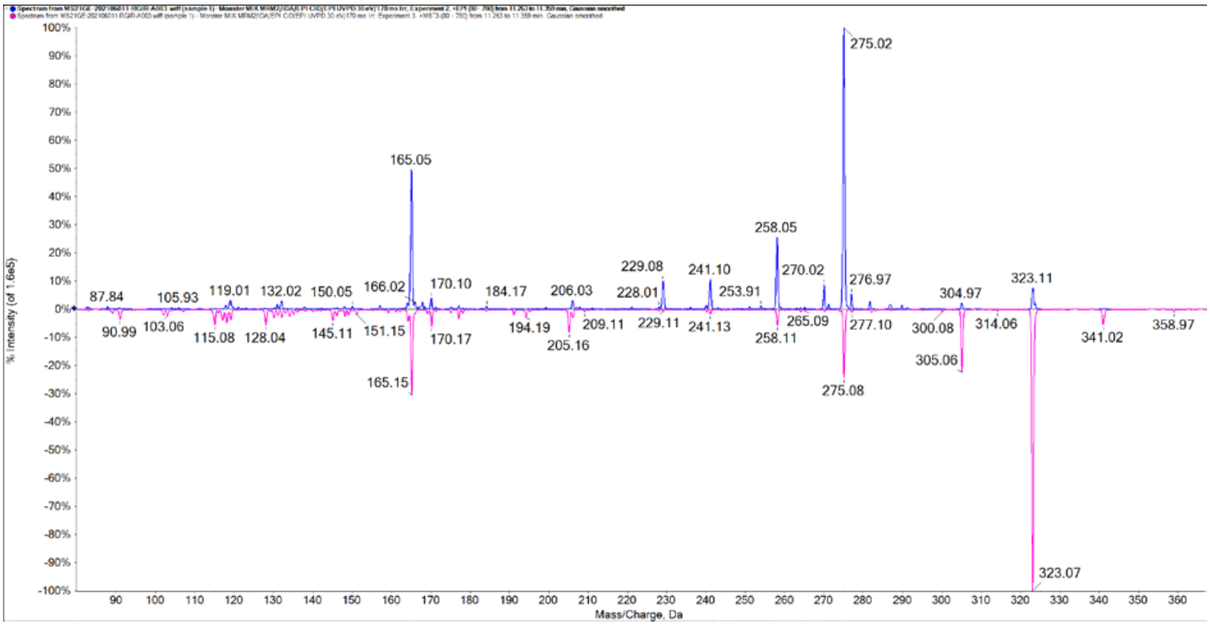

Figure S34. Chloramphenicol CID (upper) and UVPD (lower) fragmentation spectra zoom from 80 to 370 *m/z*.

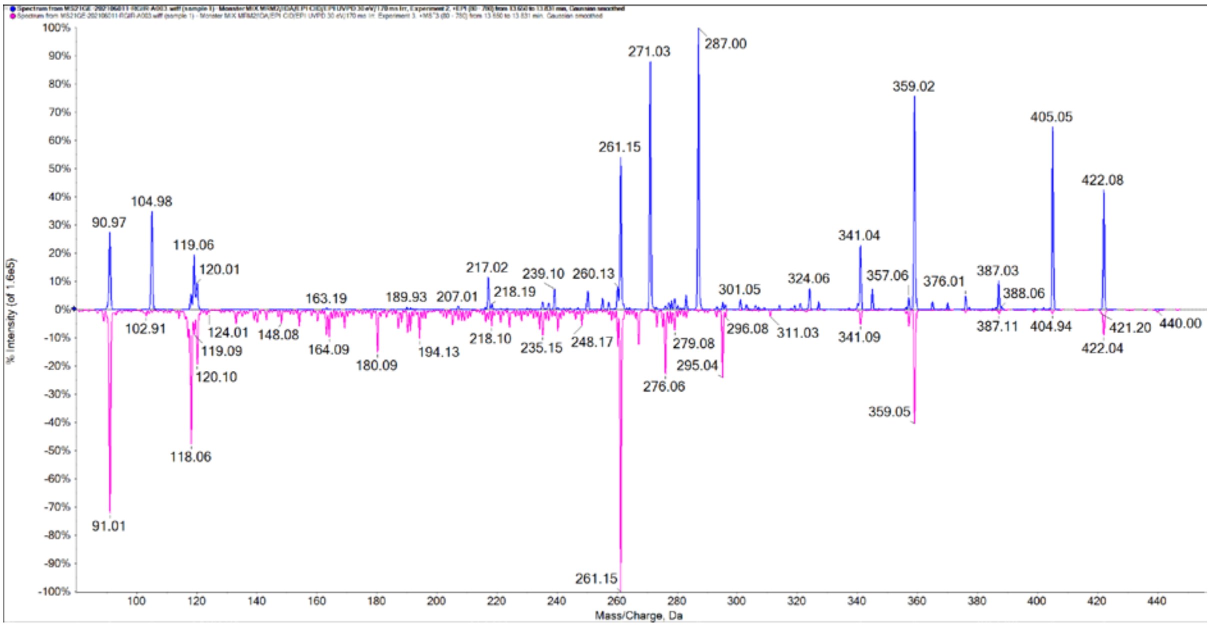

Figure S35. Bendroflumethiazide CID (upper) and UVPD (lower) fragmentation spectra zoom from 80 to 455 *m/z*.

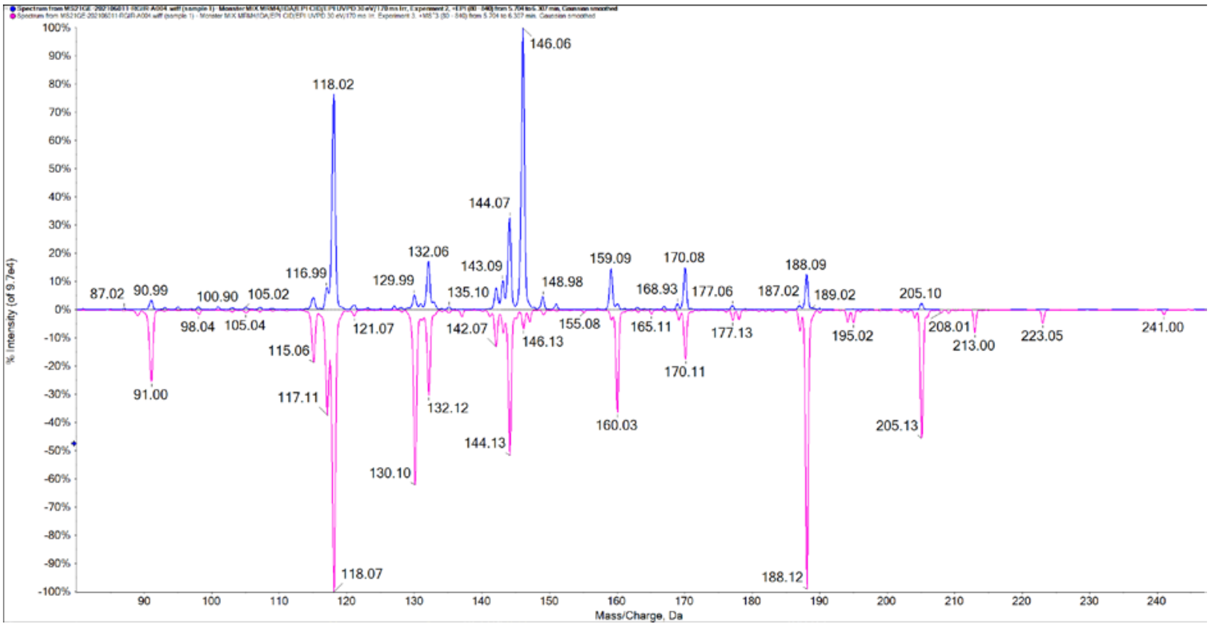

Figure S36. L-tryptophan CID (upper) and UVPD (lower) fragmentation spectra zoom from 80 to 250 *m/z*.

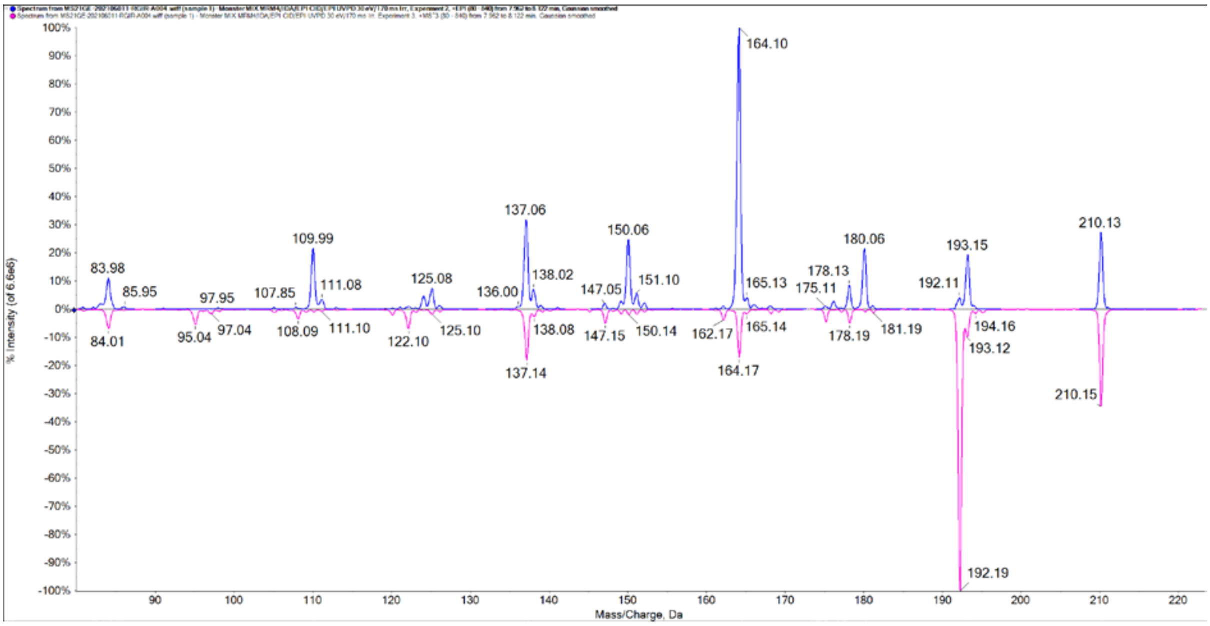

Figure S37. Minoxidil CID (upper) and UVPD (lower) fragmentation spectra zoom from 80 to 225 *m/z*.

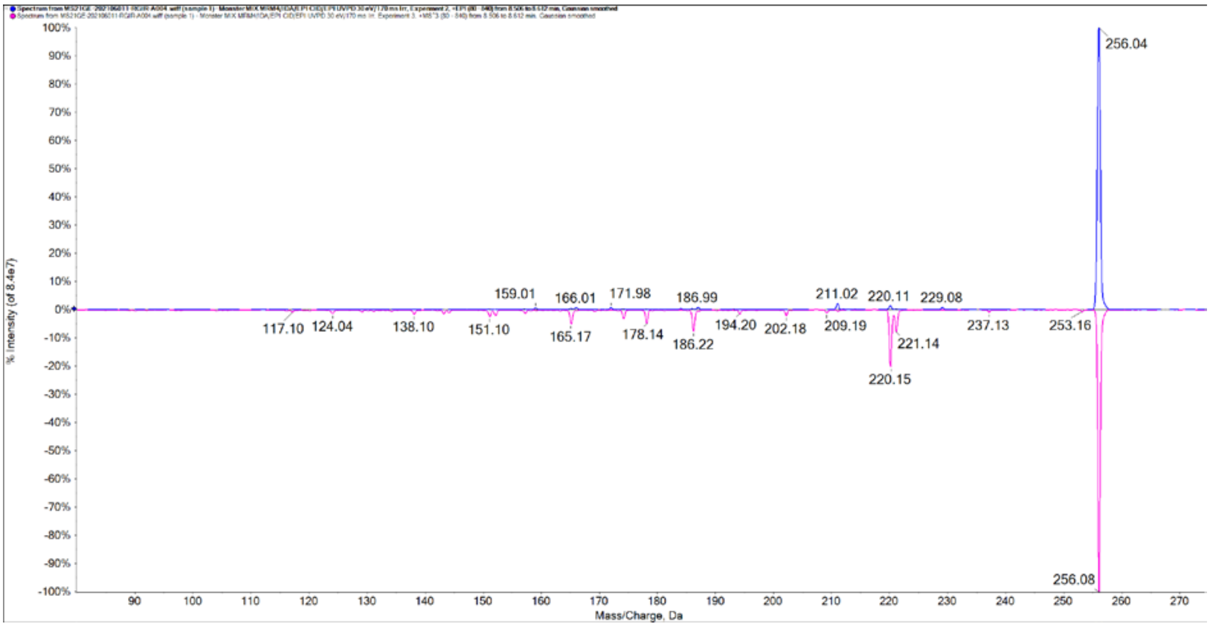

Figure S38. Lamotrigine CID (upper) and UVPD (lower) fragmentation spectra zoom from 80 to 275 *m/z*.

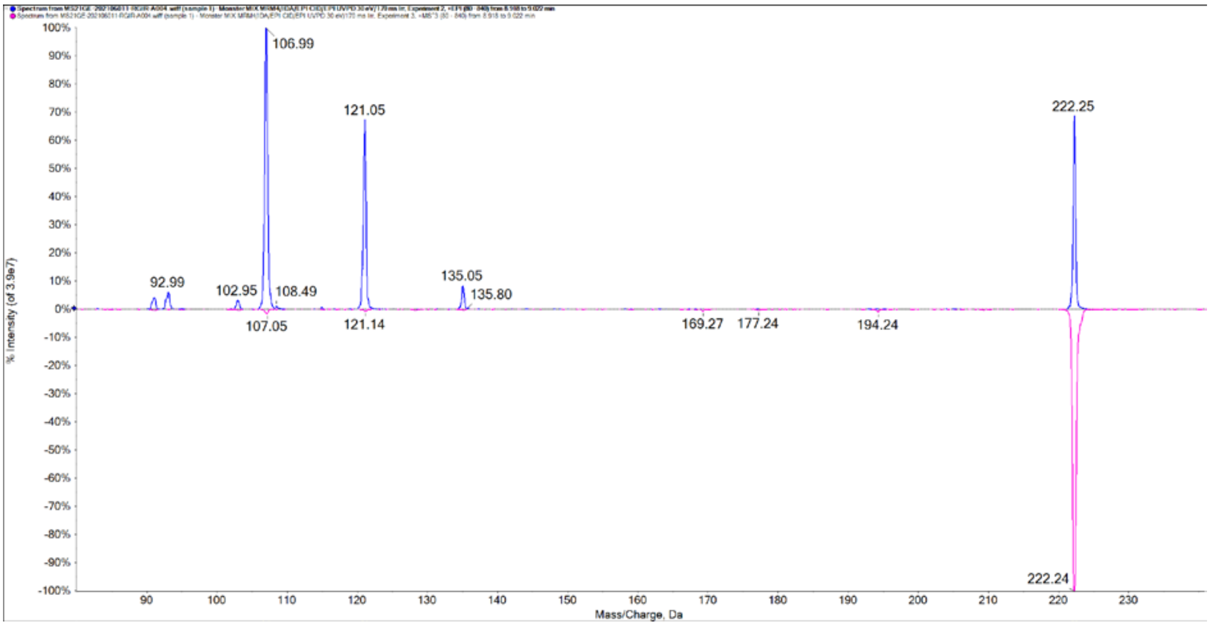

Figure S39. Tapentadol CID (upper) and UVPD (lower) fragmentation spectra zoom from 80 to 240 *m/z*.

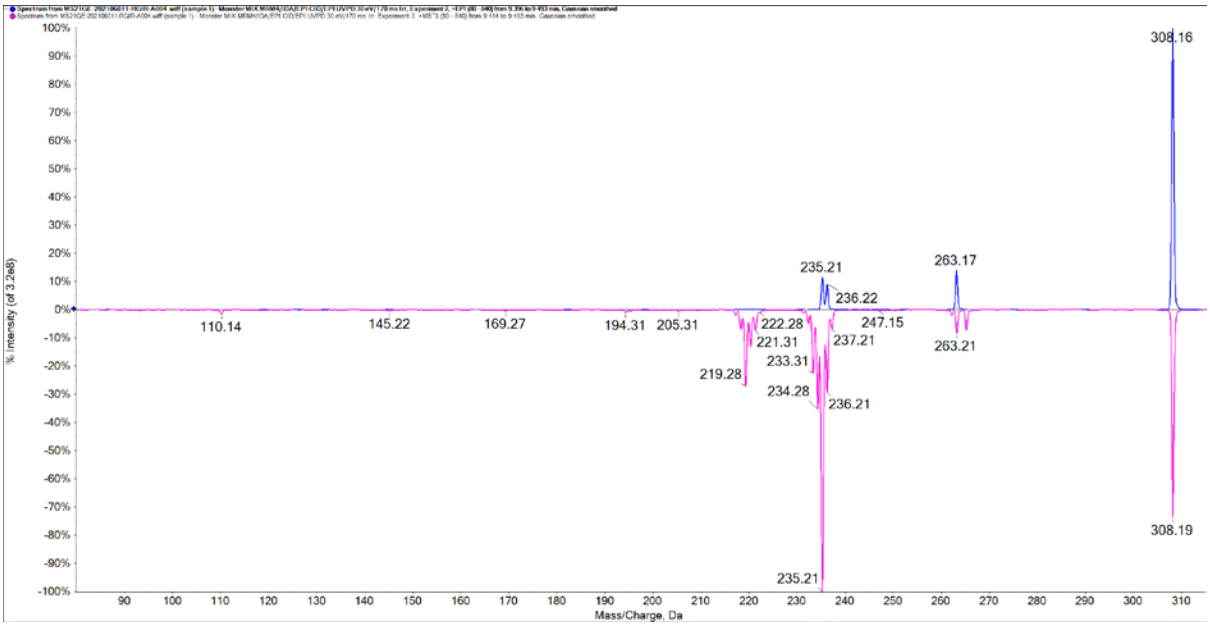

Figure S40. Zolpidem CID (upper) and UVPD (lower) fragmentation spectra zoom from 80 to 315 *m/z*.

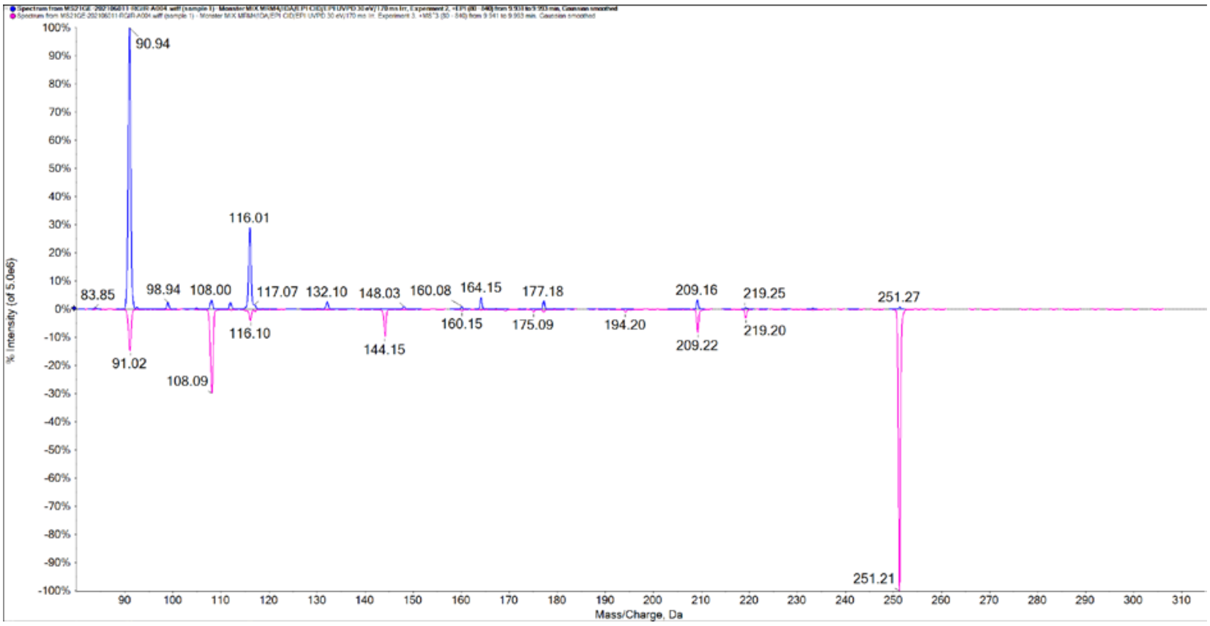

Figure S41. Lacosamide CID (upper) and UVPD (lower) fragmentation spectra zoom from 80 to 315 *m/z*.

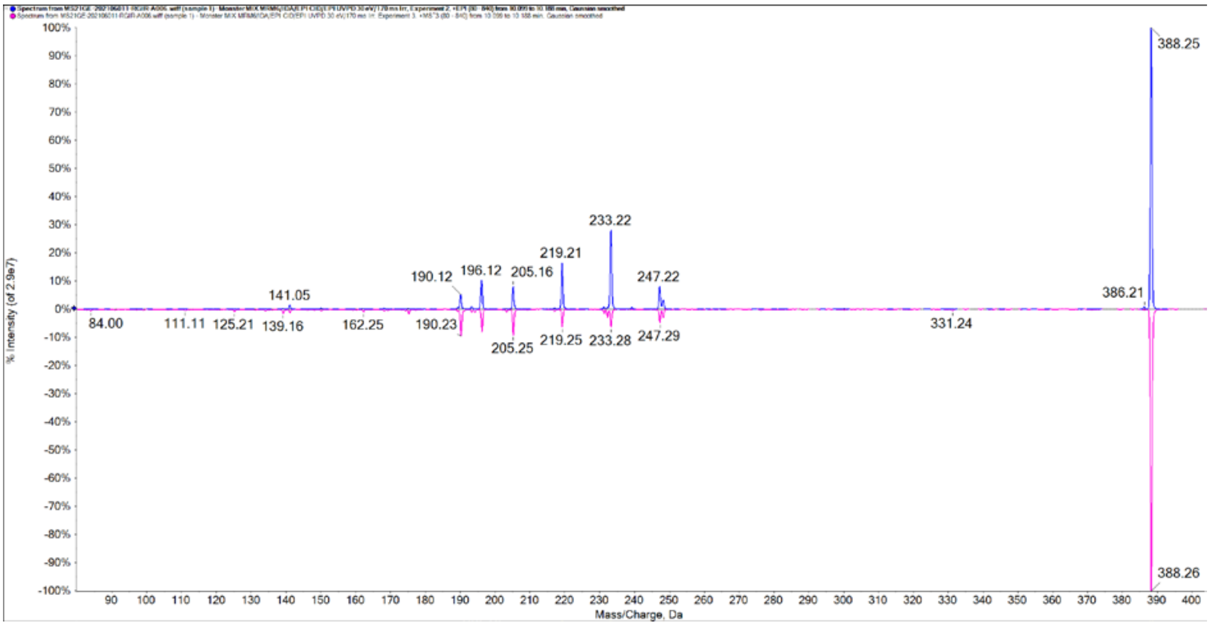

Figure S42. Urapidil CID (upper) and UVPD (lower) fragmentation spectra zoom from 80 to 400 *m/z*.

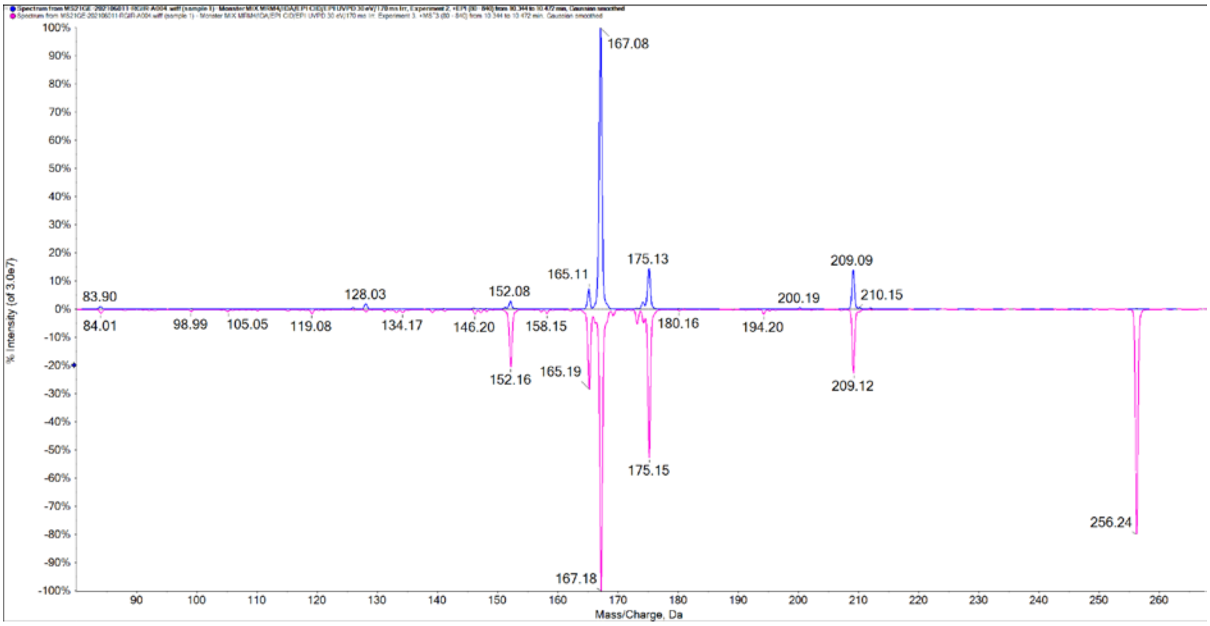

Figure S43. Imidacloprid CID (upper) and UVPD (lower) fragmentation spectra zoom from 80 to 270 *m/z*.

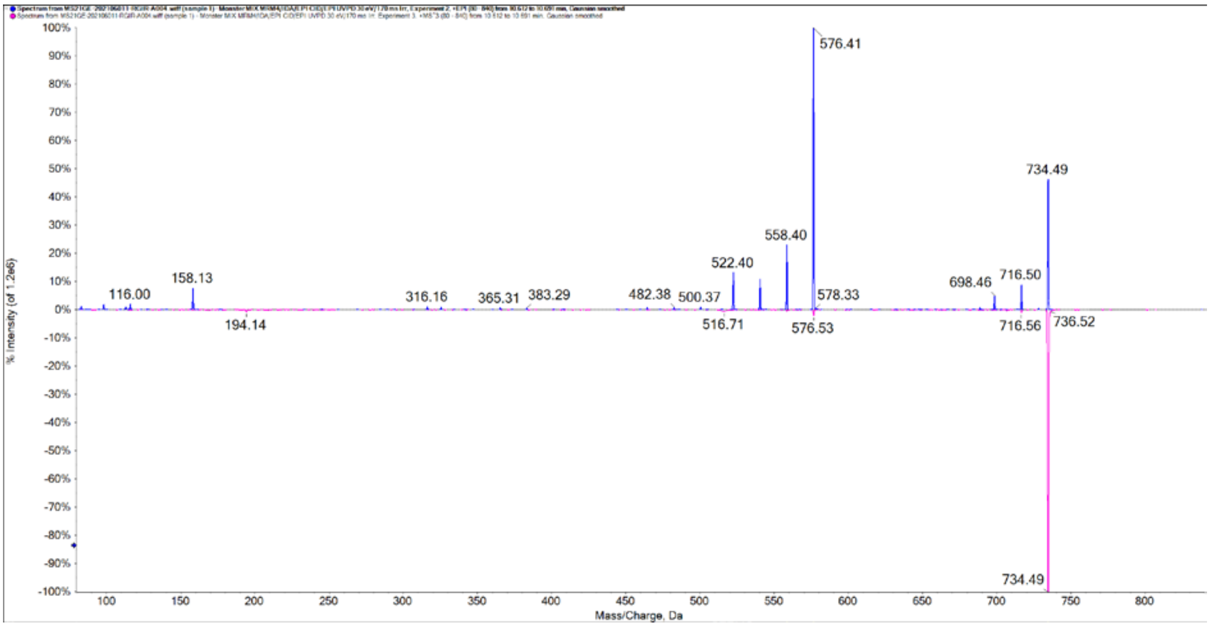

Figure S44. Erythromycin CID (upper) and UVPD (lower) fragmentation spectra zoom from 80 to 840 *m/z*.

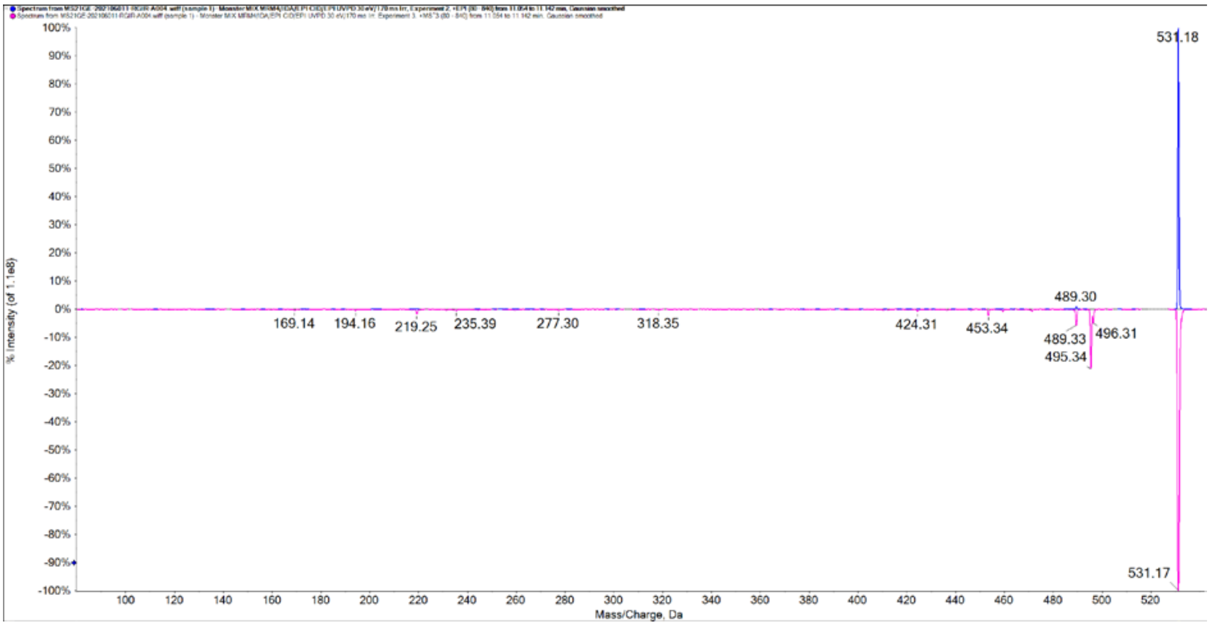

Figure S45. Ketoconazole CID (upper) and UVPD (lower) fragmentation spectra zoom from 80 to 530 *m/z*.

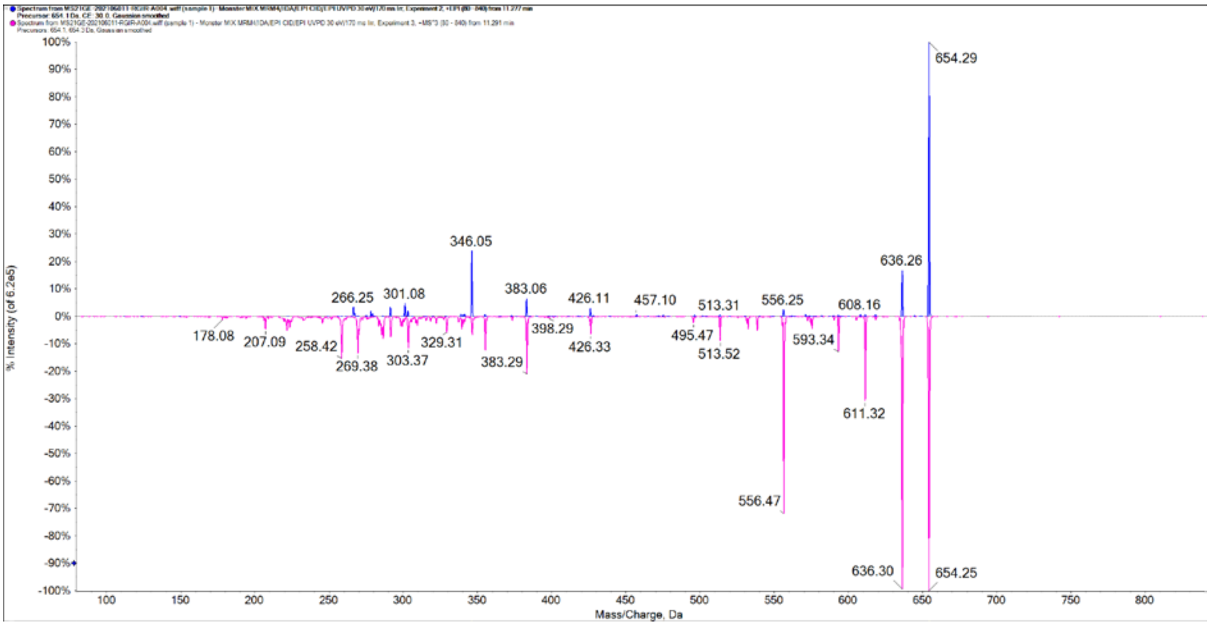

Figure S46. Bromocriptine CID (upper) and UVPD (lower) fragmentation spectra zoom from 80 to 840 *m/z*.

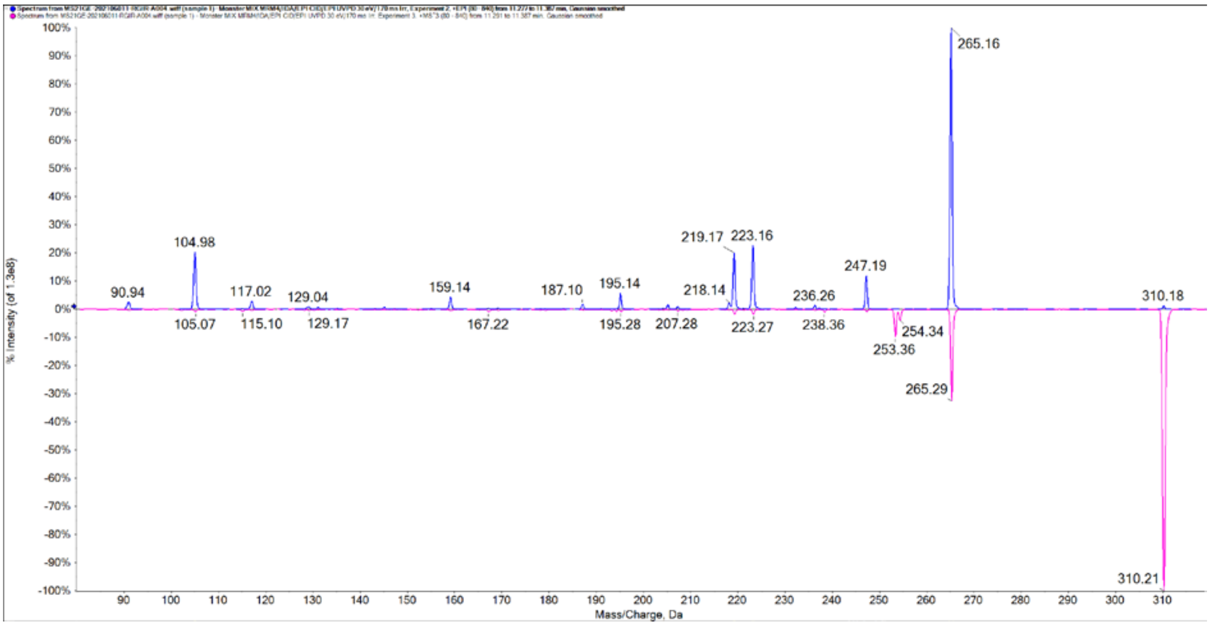

Figure S47. Methadone CID (upper) and UVPD (lower) fragmentation spectra zoom from 80 to 320 *m/z*.

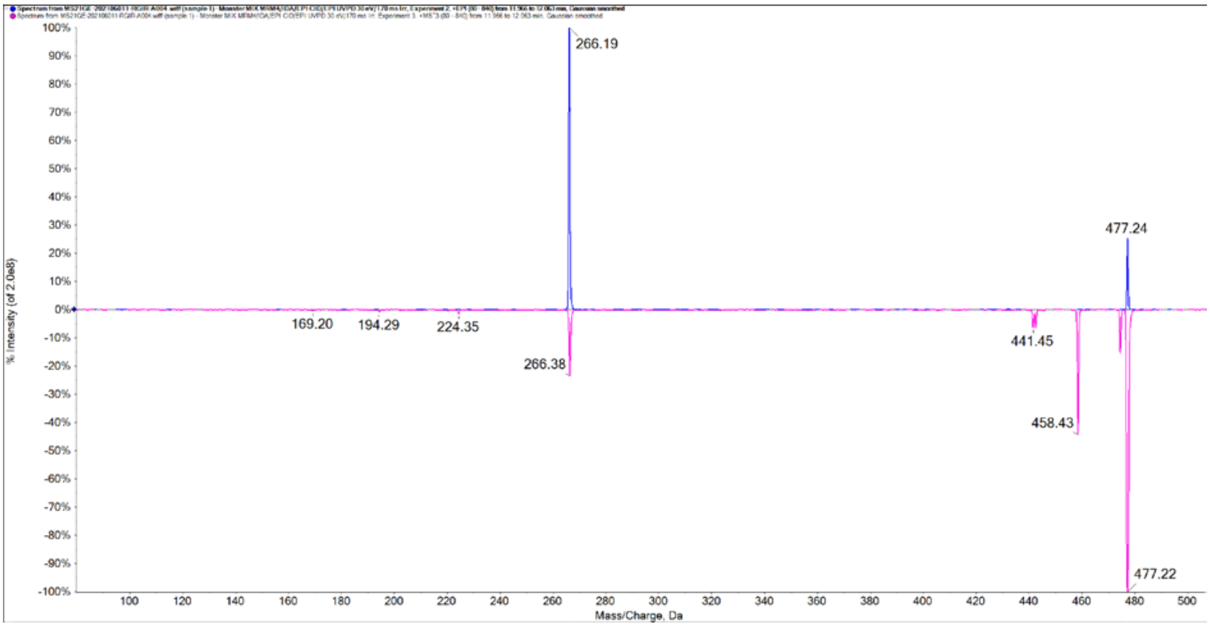

Figure S48. Loperamide CID (upper) and UVPD (lower) fragmentation spectra zoom from 80 to 500 *m/z*.

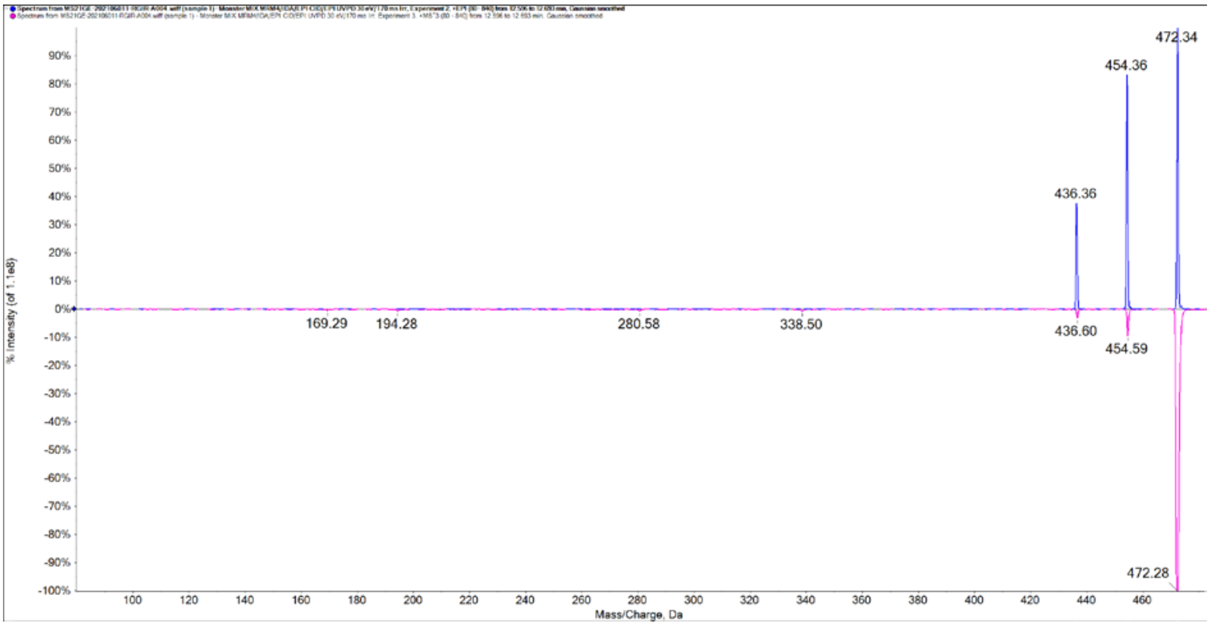

Figure S49. Terfenadine CID (upper) and UVPD (lower) fragmentation spectra zoom from 80 to 480 *m/z*.

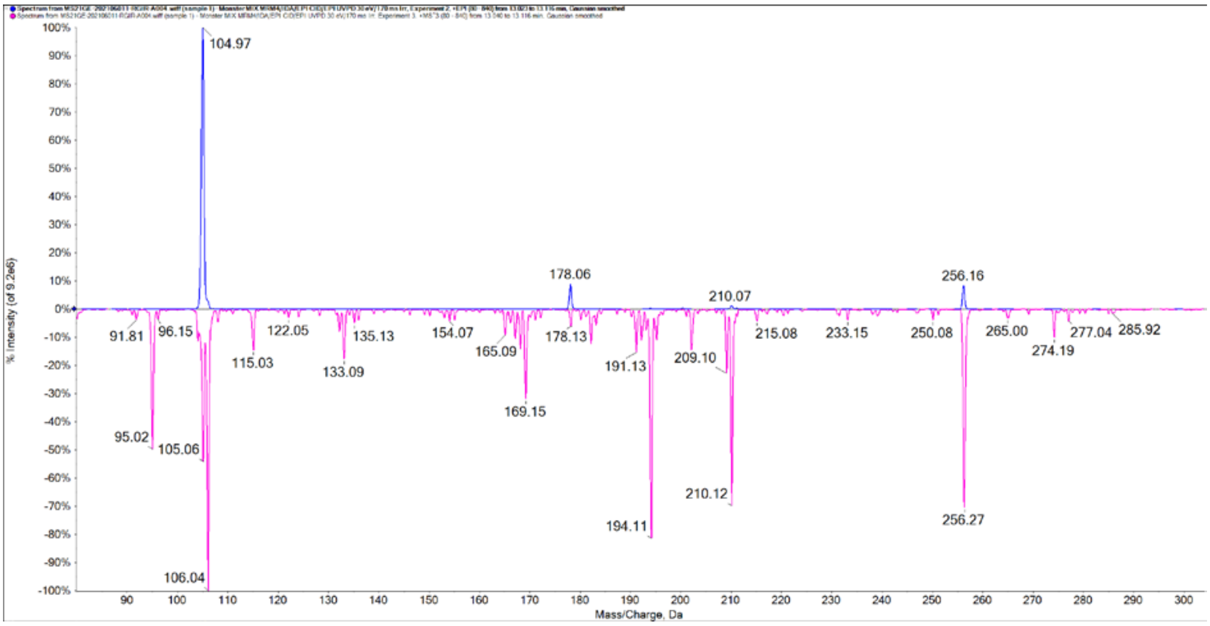

Figure S50. Ketorolac CID (upper) and UVPD (lower) fragmentation spectra zoom from 80 to 300 *m/z*.

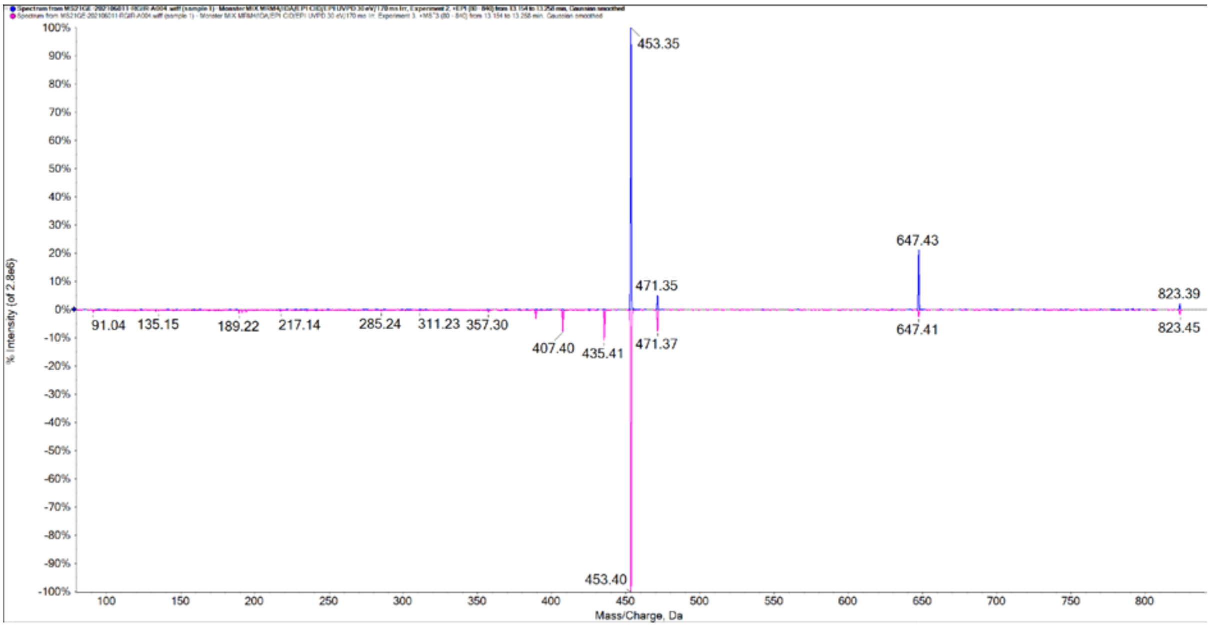

Figure S51. Glycyrrhizic acid CID (upper) and UVPD (lower) fragmentation spectra zoom from 80 to 840 *m/z*.

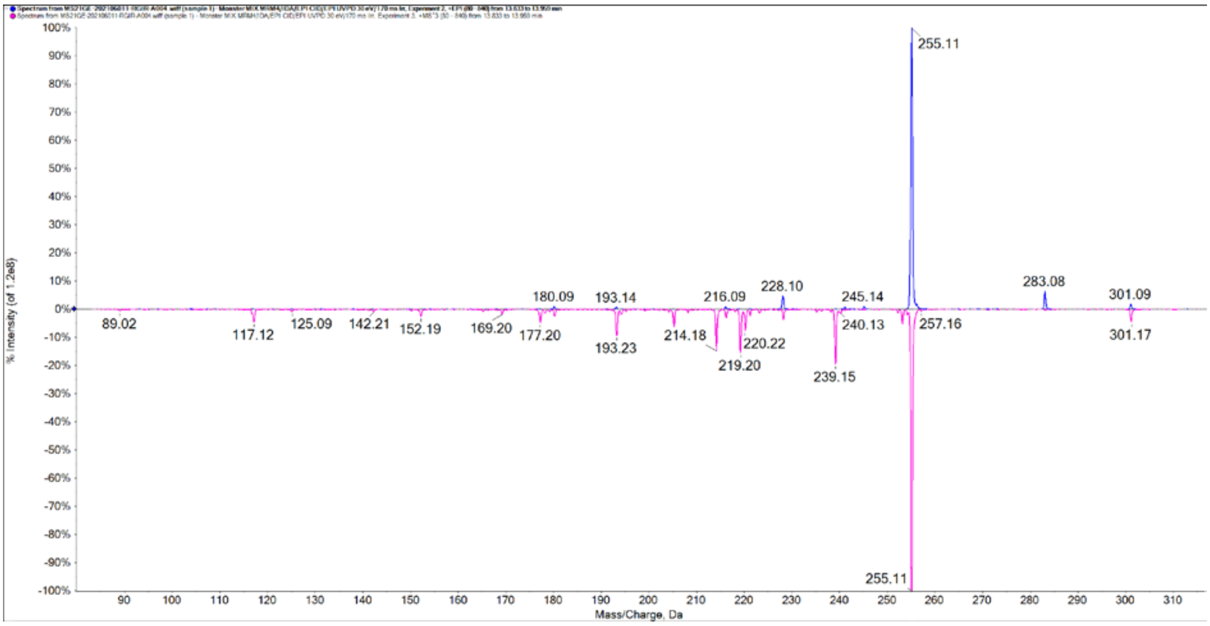

Figure S52. Temazepam CID (upper) and UVPD (lower) fragmentation spectra zoom from 80 to 315 *m/z*.

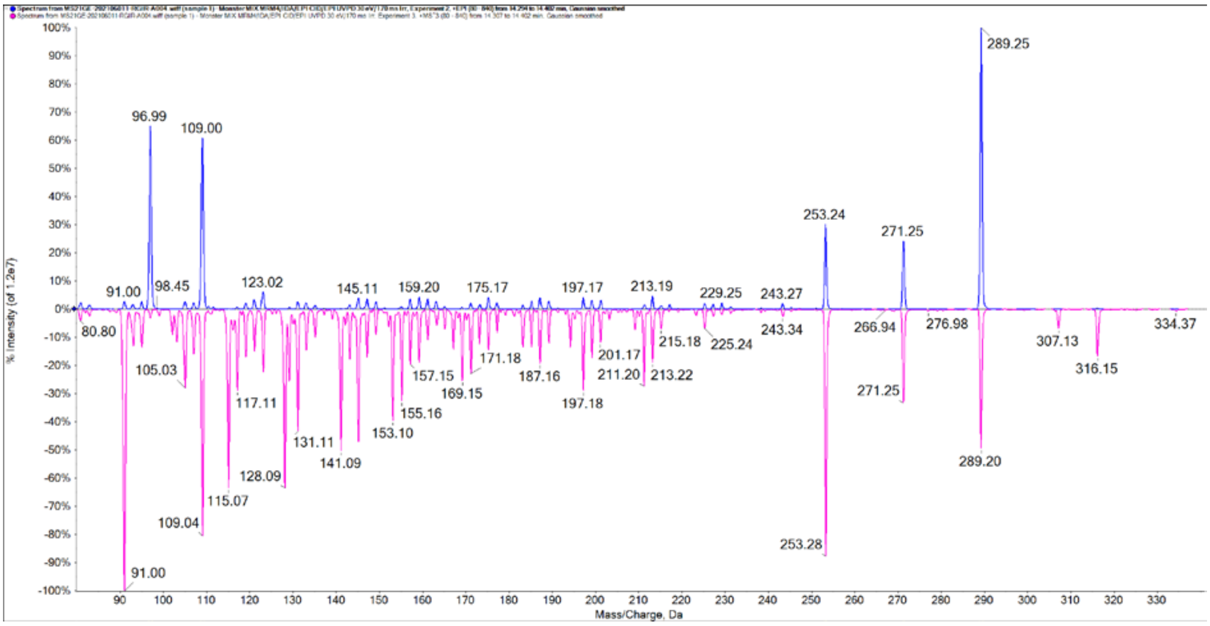

Figure S53. Testosterone CID (upper) and UVPD (lower) fragmentation spectra zoom from 80 to 340 *m/z*.

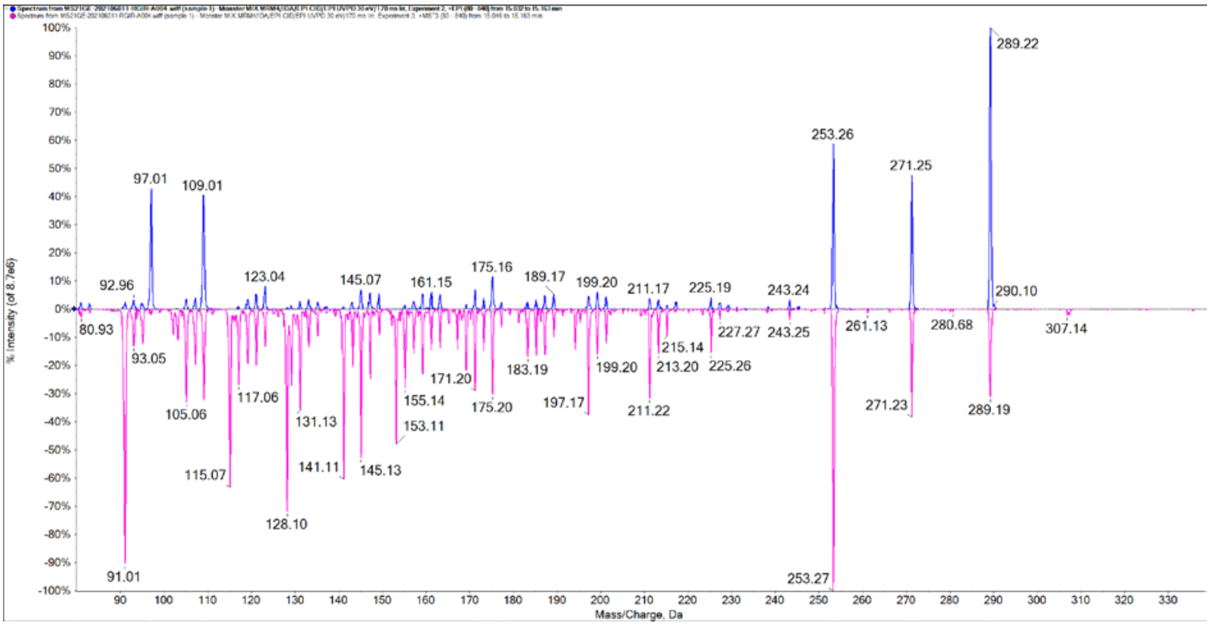

Figure S54. Epitestosterone CID (upper) and UVPD (lower) fragmentation spectra zoom from 80 to 340 *m/z*.

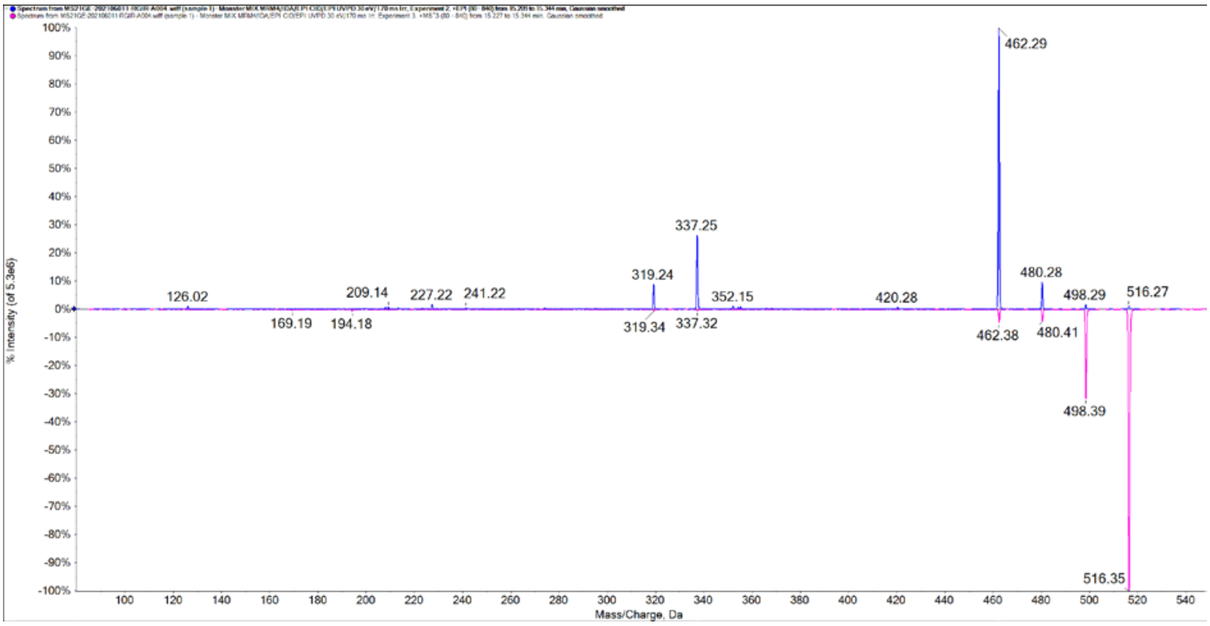

Figure S55. Taurocholic acid CID (upper) and UVPD (lower) fragmentation spectra zoom from 80 to 540 *m/z*.

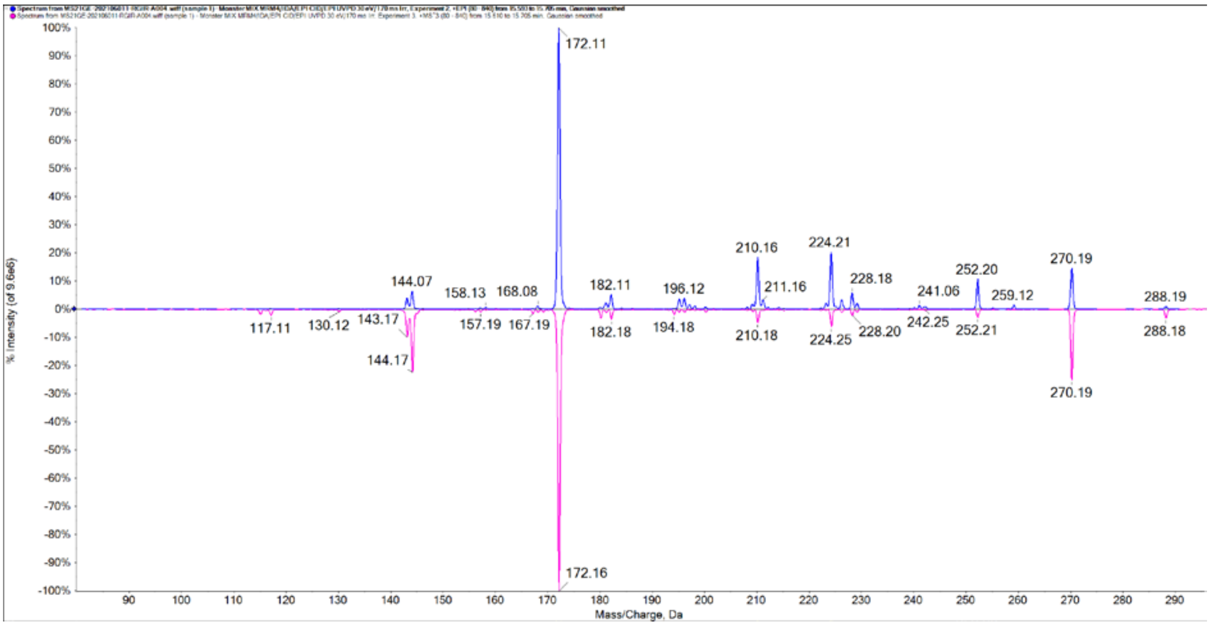

Figure S56. Etodolac CID (upper) and UVPD (lower) fragmentation spectra zoom from 80 to 295 *m/z*.

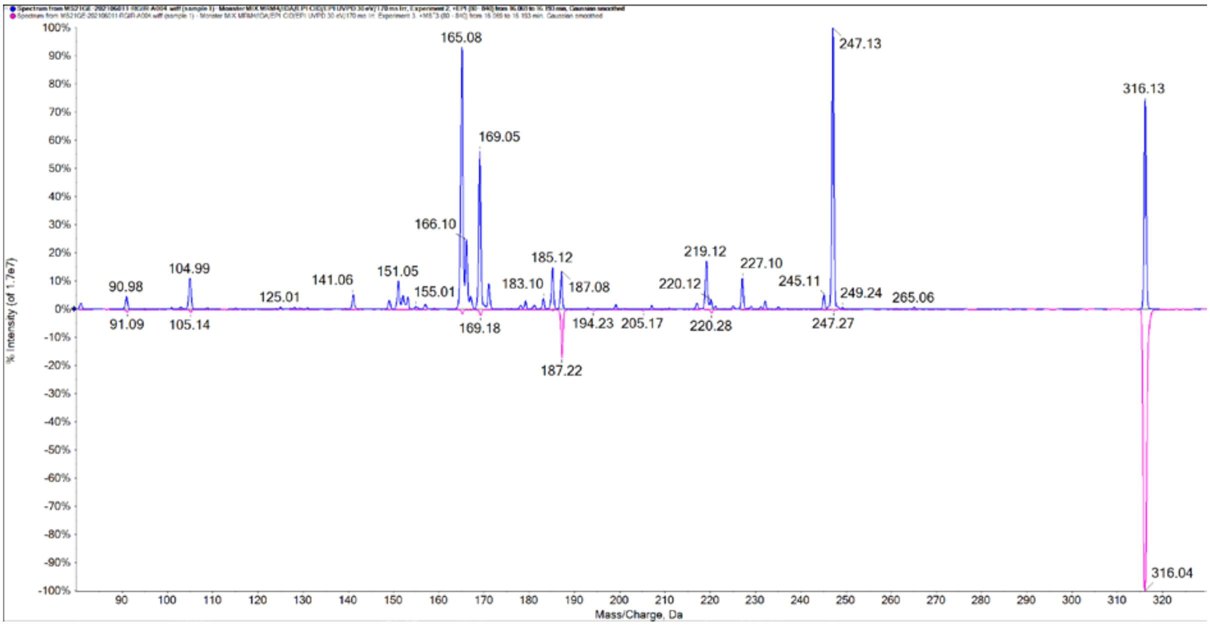

Figure S57. Flusilazole CID (upper) and UVPD (lower) fragmentation spectra zoom from 80 to 330 *m/z*.

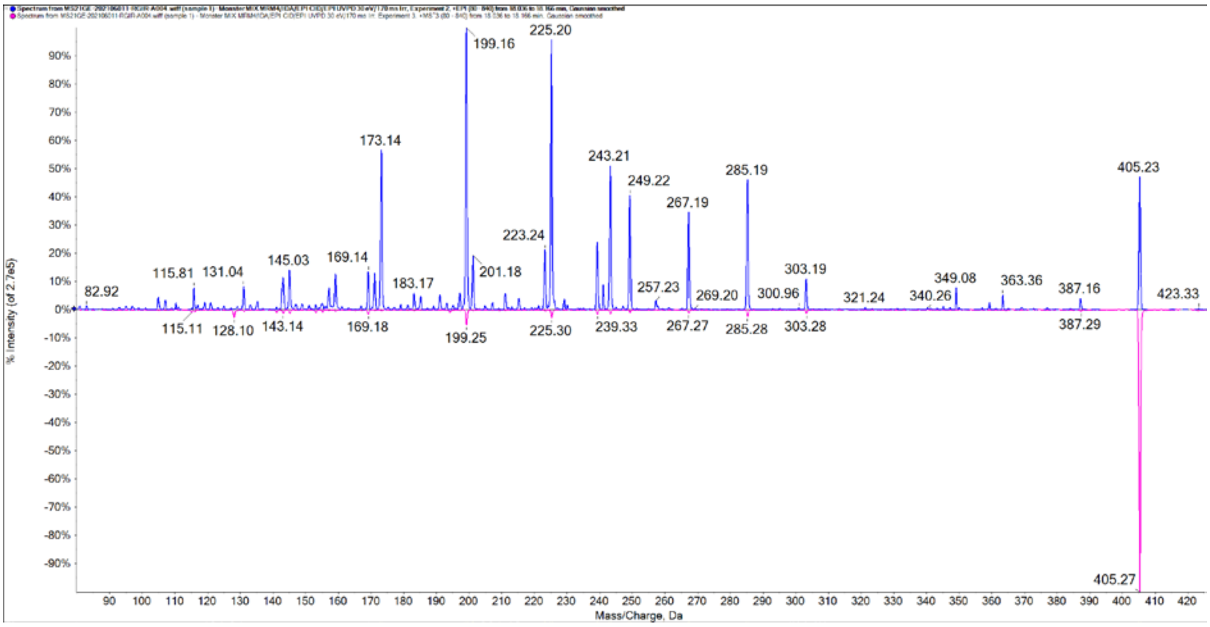

Figure S58. Lovastatin CID (upper) and UVPD (lower) fragmentation spectra zoom from 80 to 425 *m/z*.

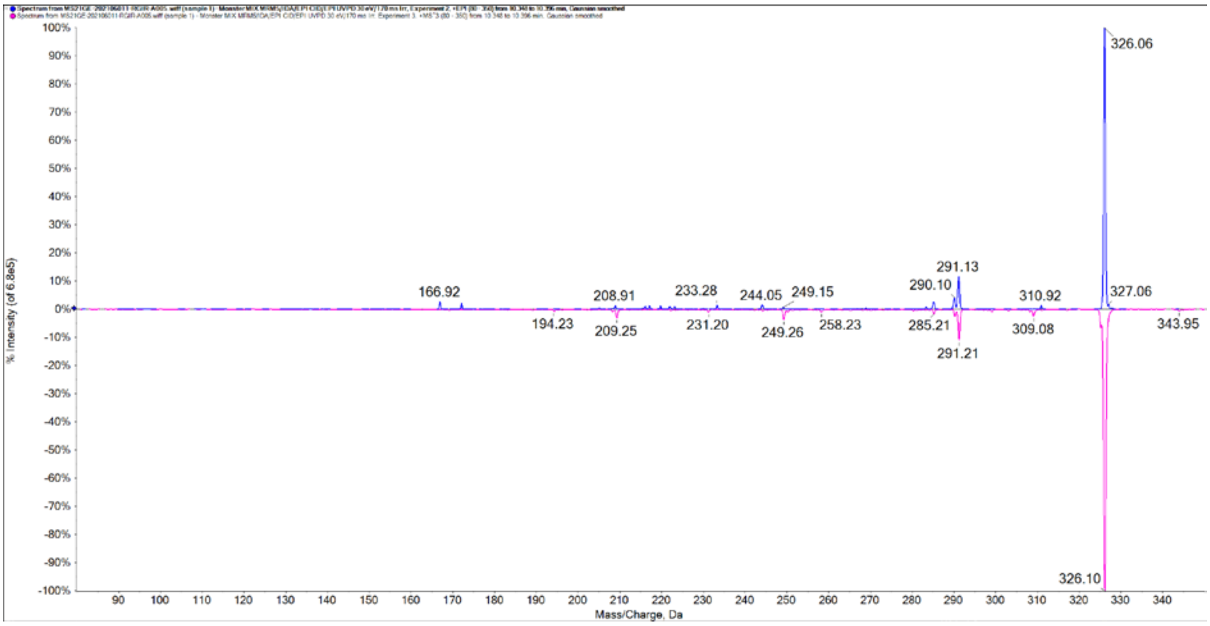

Figure S59. Midazolam CID (upper) and UVPD (lower) fragmentation spectra zoom from 80 to 350 *m/z*.

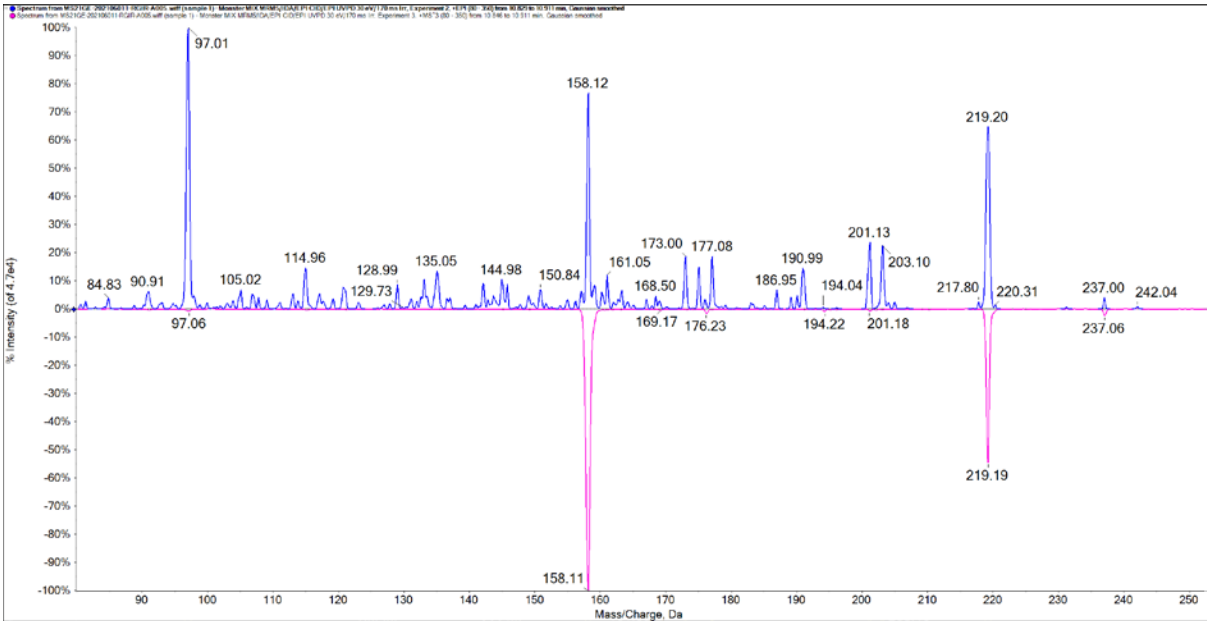

Figure S60. Meprobamate CID (upper) and UVPD (lower) fragmentation spectra zoom from 80 to 250 *m/z*.

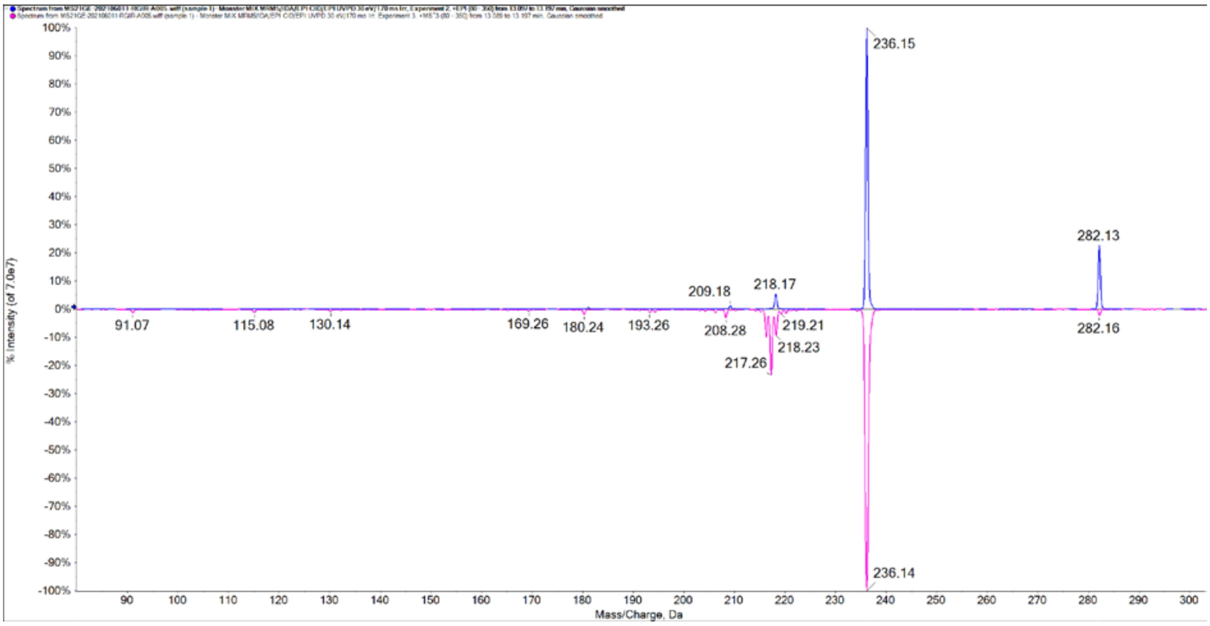

Figure S61. Indoprofen CID (upper) and UVPD (lower) fragmentation spectra zoom from 80 to 300 *m/z*.

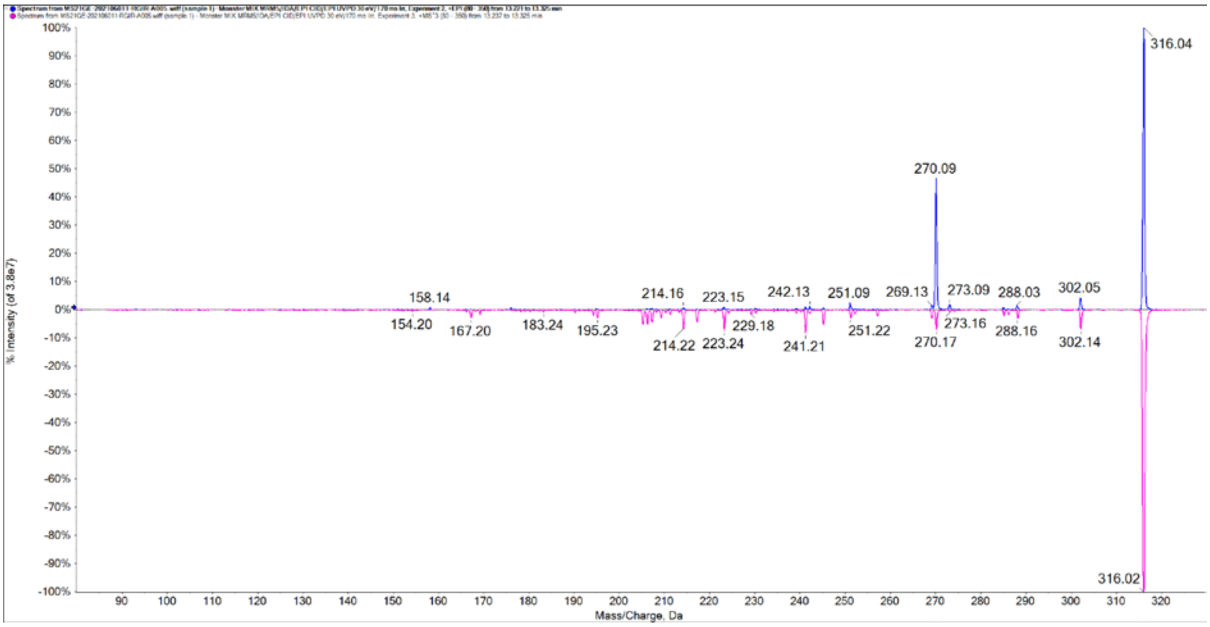

Figure S62. Clonazepam CID (upper) and UVPD (lower) fragmentation spectra zoom from 80 to 330 *m/z*.

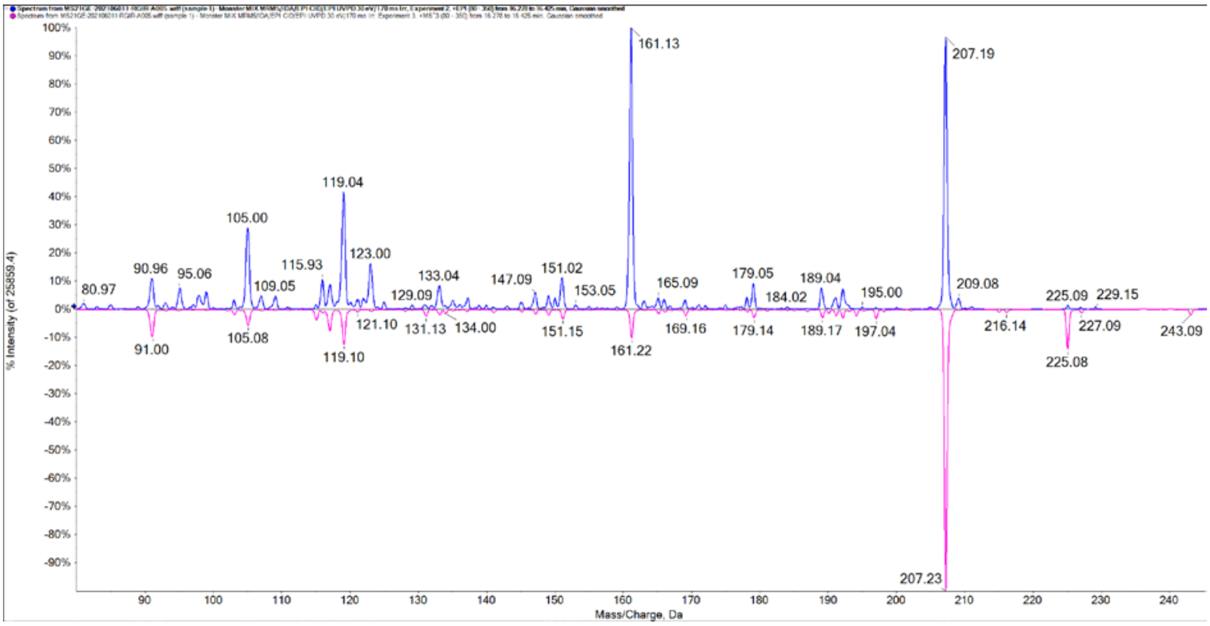

Figure S63. Ibuprofen CID (upper) and UVPD (lower) fragmentation spectra zoom from 80 to 250 *m/z*.

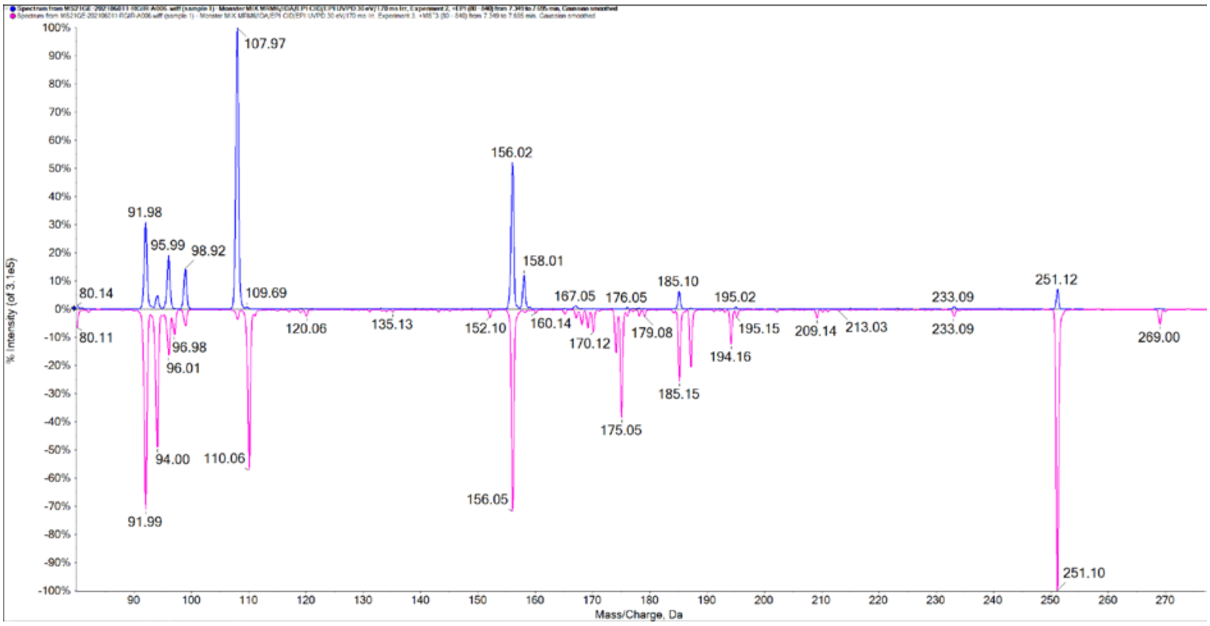

Figure S64. Sulfadiazine CID (upper) and UVPD (lower) fragmentation spectra zoom from 80 to 280 *m/z*.

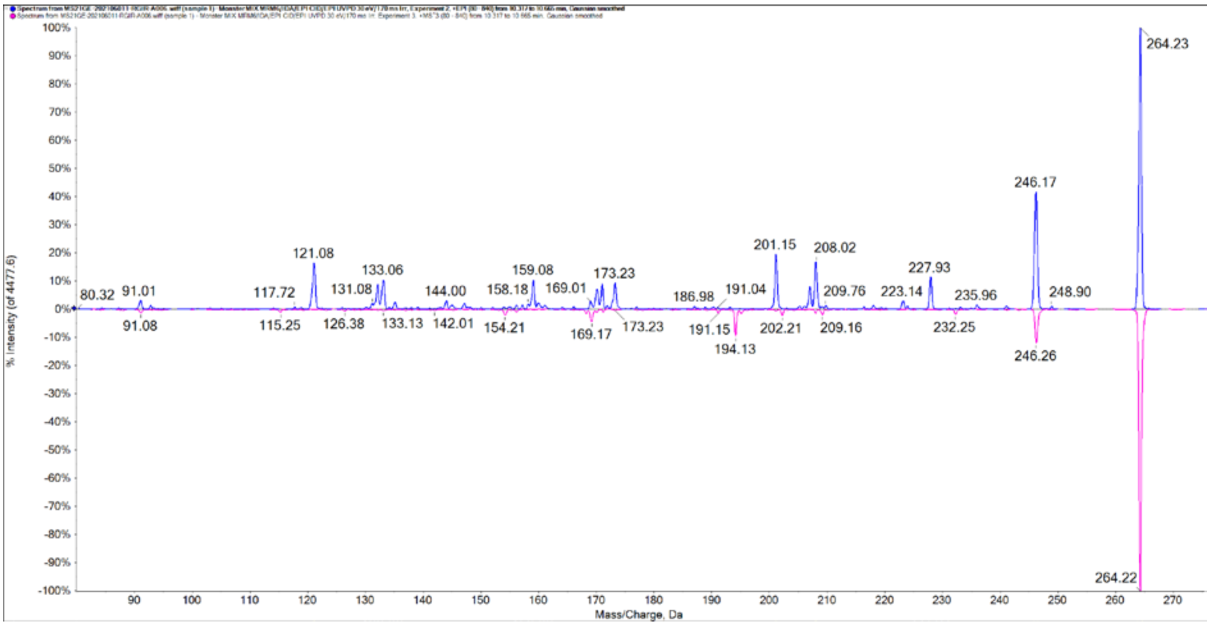

Figure S65. Tramadol CID (upper) and UVPD (lower) fragmentation spectra zoom from 80 to 275 *m/z*.

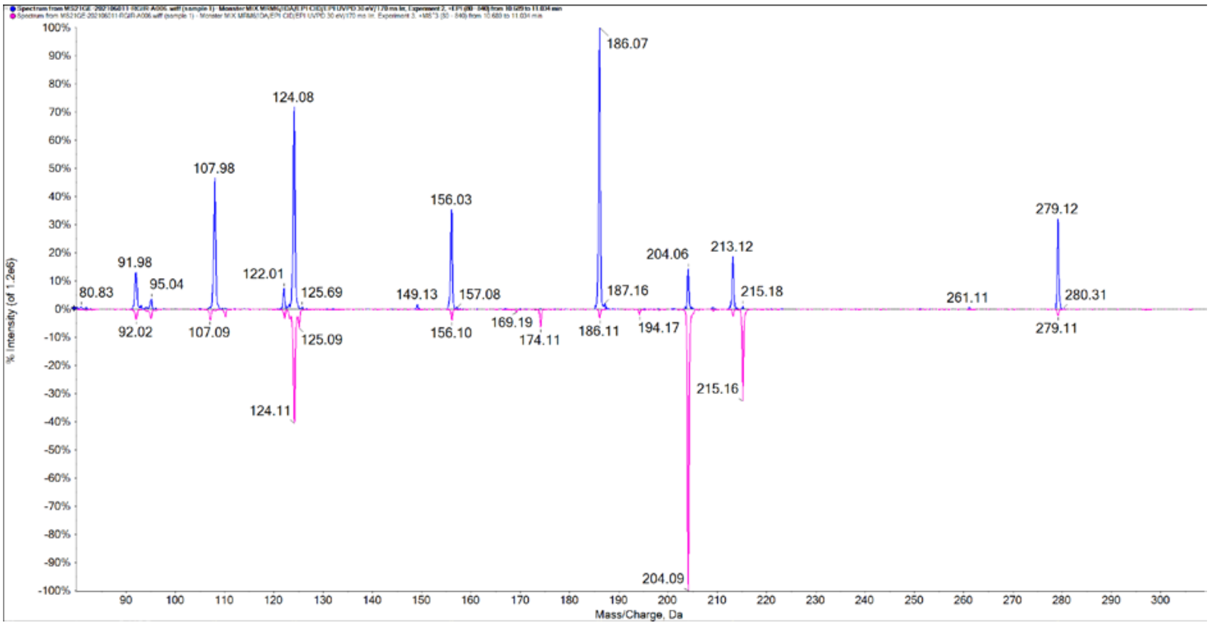

Figure S66. Sulfamidine CID (upper) and UVPD (lower) fragmentation spectra zoom from 80 to 310 *m/z*.

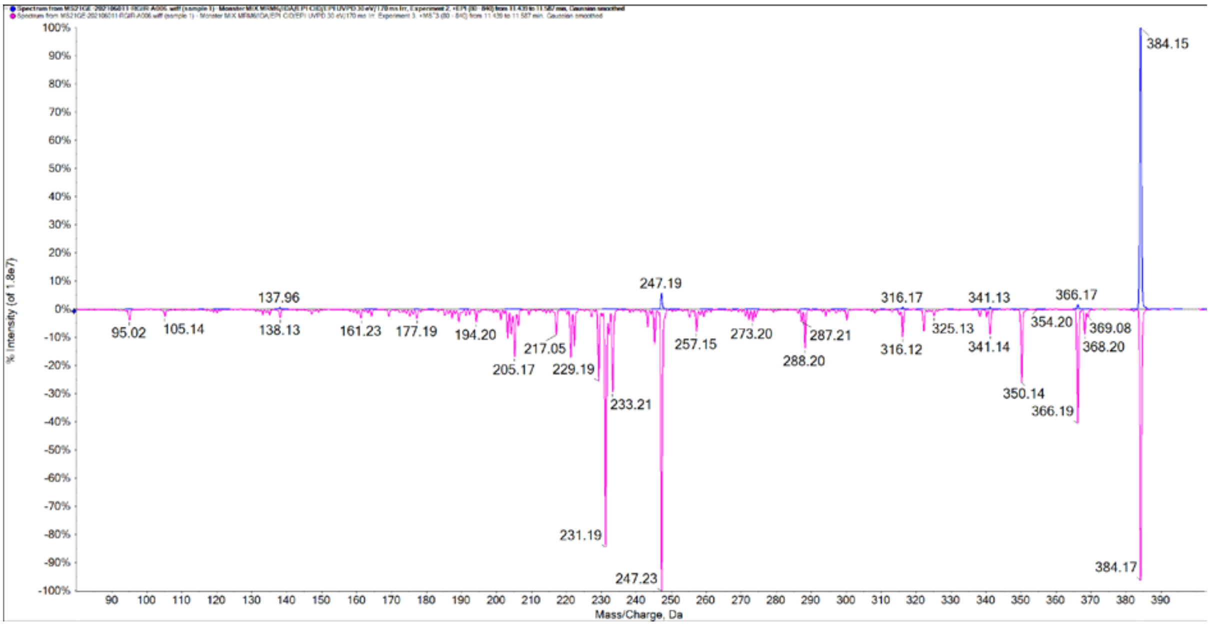

Figure S67. Prazosin CID (upper) and UVPD (lower) fragmentation spectra zoom from 80 to 400 *m/z*.

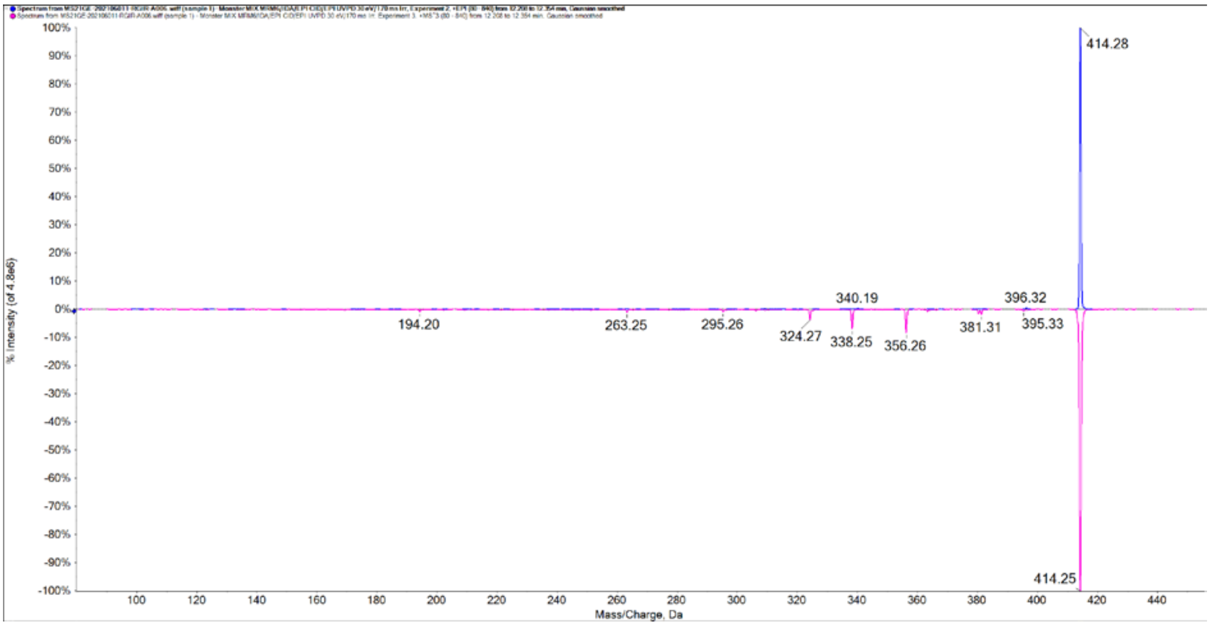

Figure S68. Norbuprenorphine CID (upper) and UVPD (lower) fragmentation spectra zoom from 80 to 455 *m/z*.

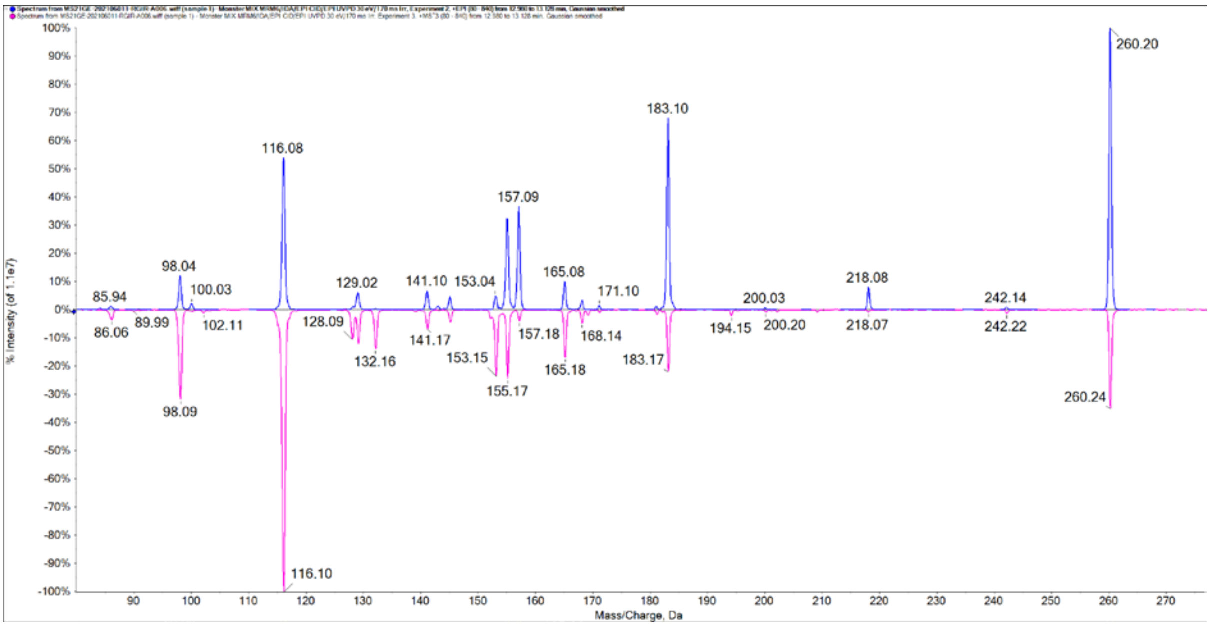

Figure S69. Propranolol CID (upper) and UVPD (lower) fragmentation spectra zoom from 80 to 275 *m/z*.

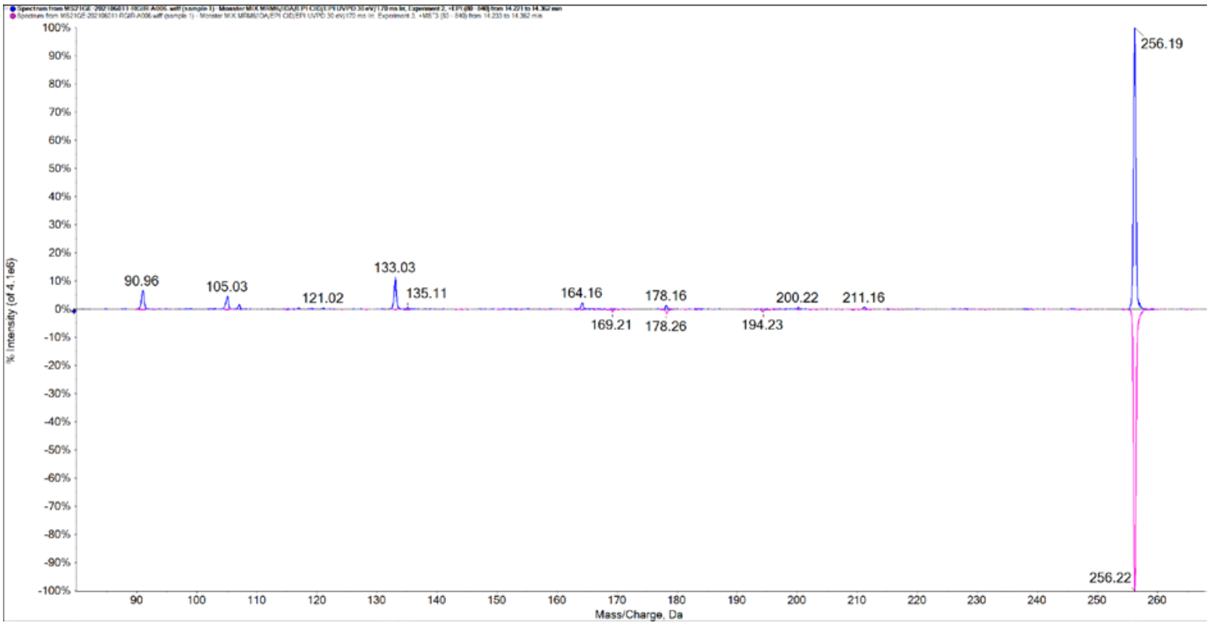

Figure S70. Phenyltoloxamine CID (upper) and UVPD (lower) fragmentation spectra zoom from 80 to 270 *m/z*.

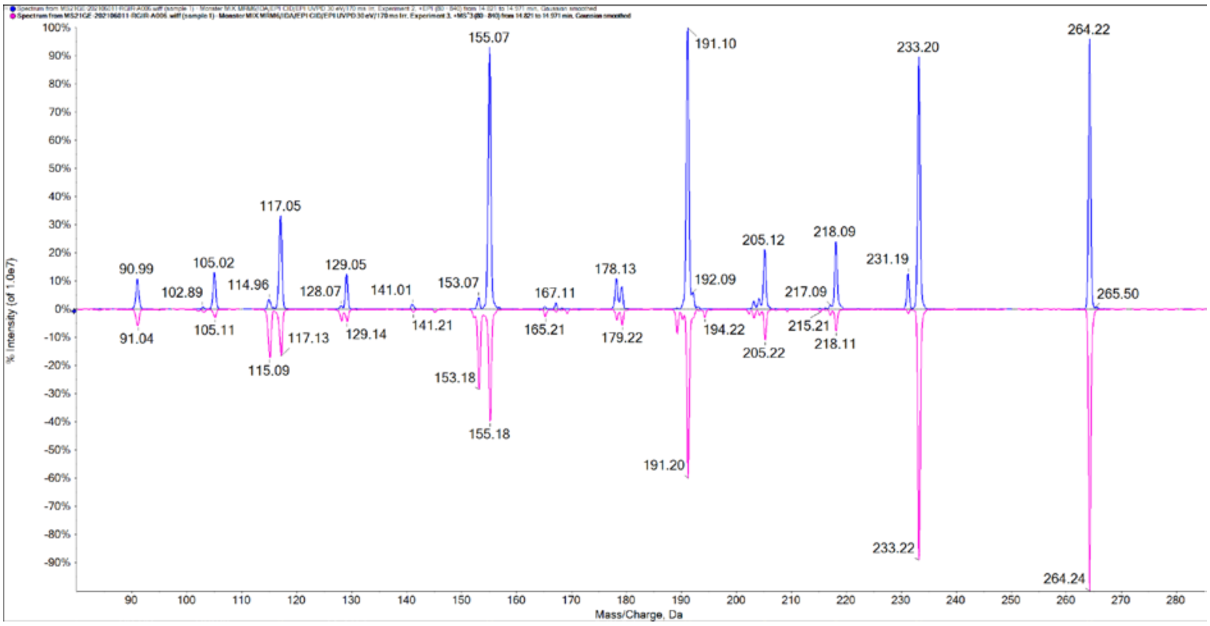

Figure S71. Nortriptyline CID (upper) and UVPD (lower) fragmentation spectra zoom from 80 to 285 *m/z*.

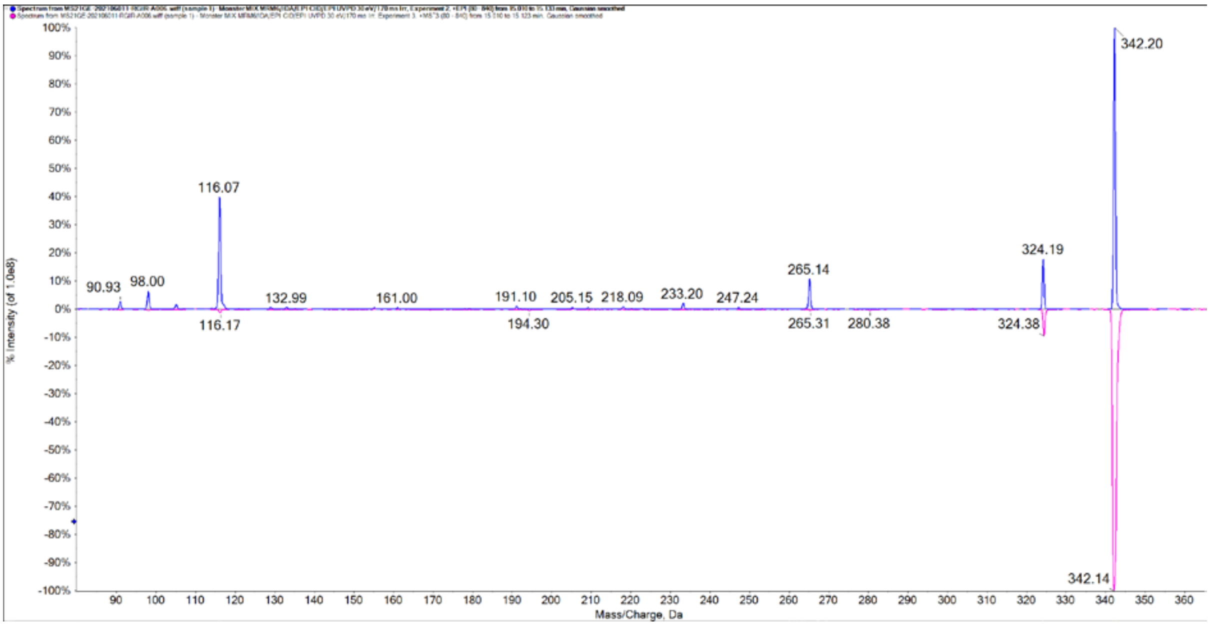

Figure S72. Propafenone CID (upper) and UVPD (lower) fragmentation spectra zoom from 80 to 360 *m/z*.

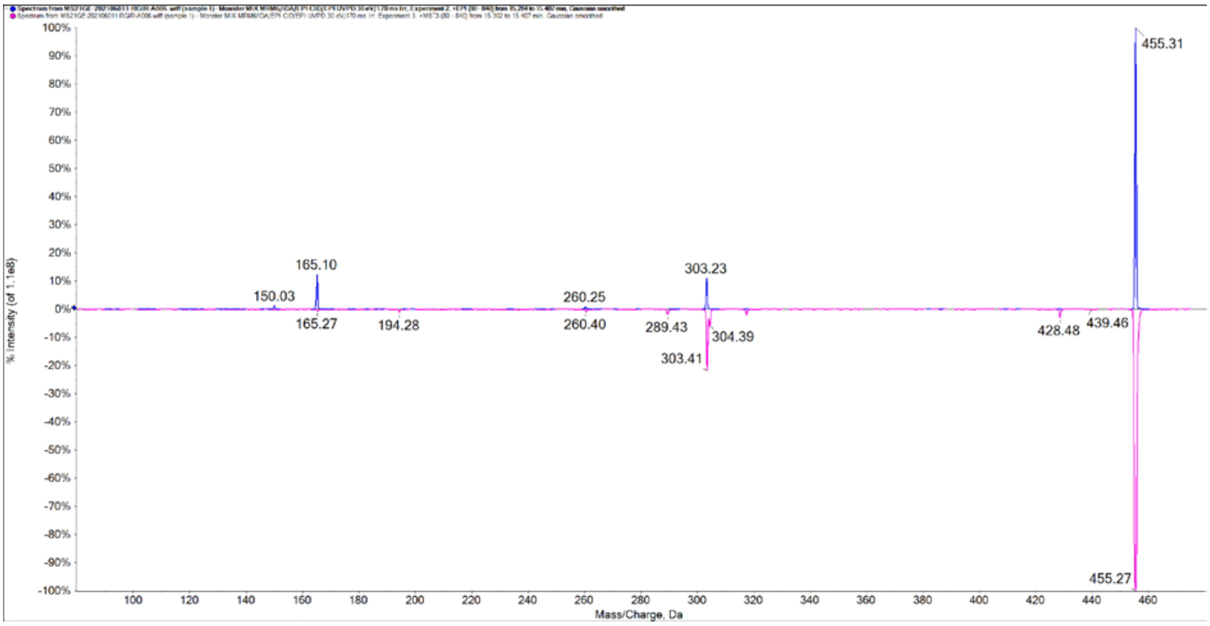

Figure S73. Verapamil CID (upper) and UVPD (lower) fragmentation spectra zoom from 80 to 470 *m/z*.

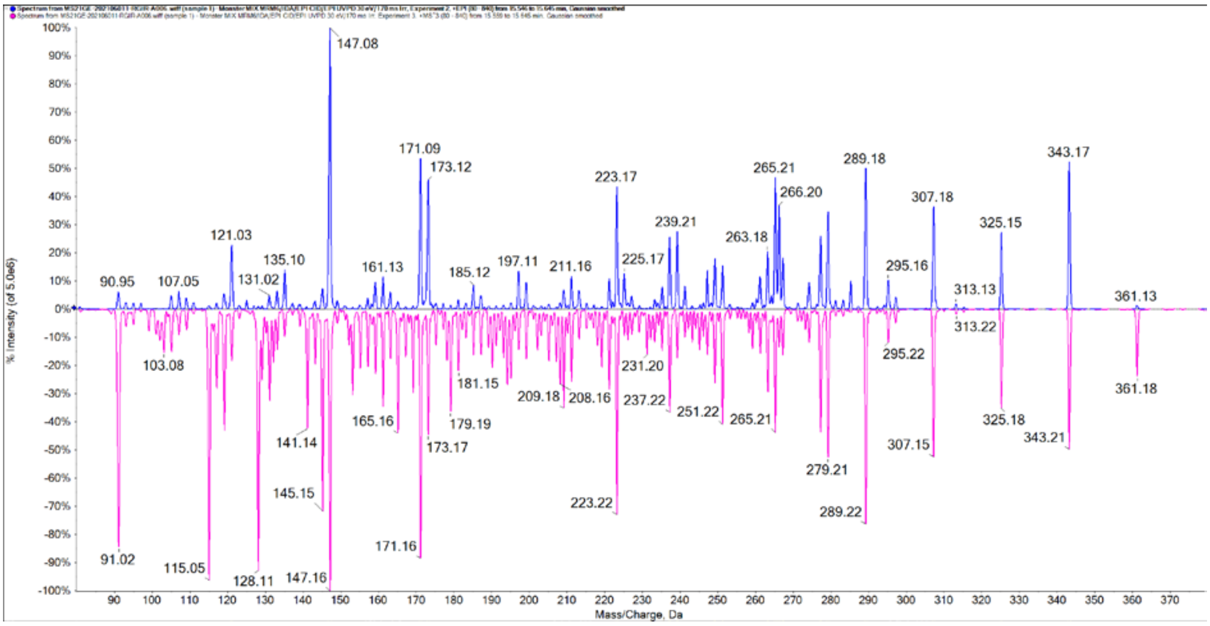

Figure S74. Prednisolone CID (upper) and UVPD (lower) fragmentation spectra zoom from 80 to 380 *m/z*.

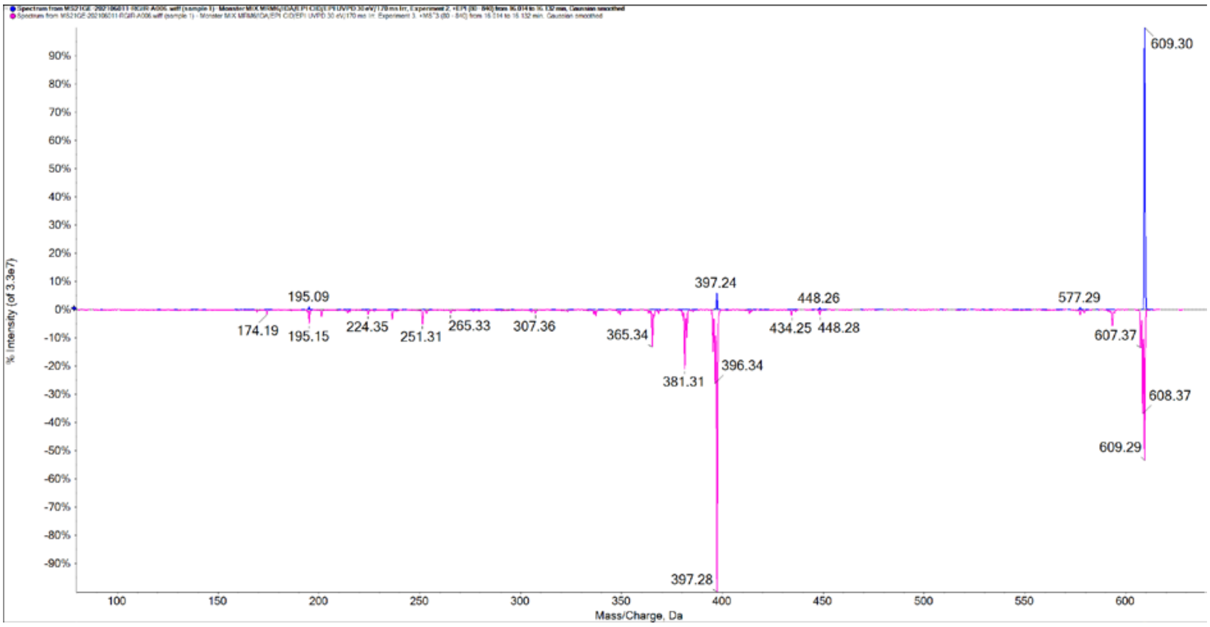

Figure S75. Reserpine CID (upper) and UVPD (lower) fragmentation spectra zoom from 80 to 640 *m/z*.

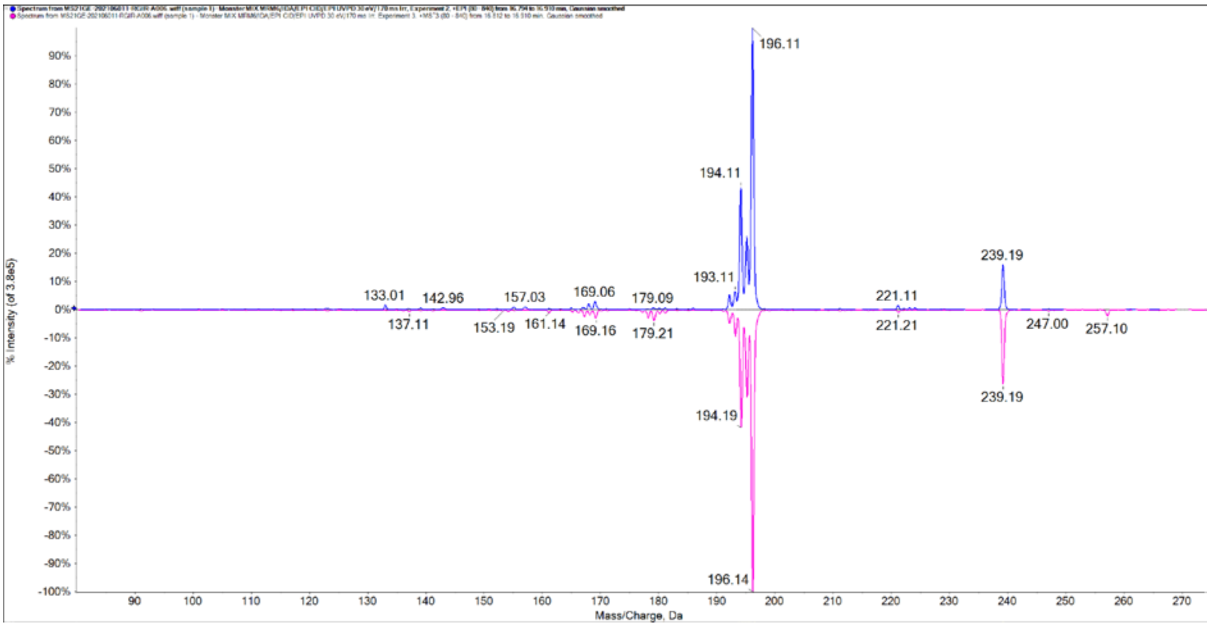

Figure S76. Secobarbital CID (upper) and UVPD (lower) fragmentation spectra zoom from 80 to 270 *m/z*.

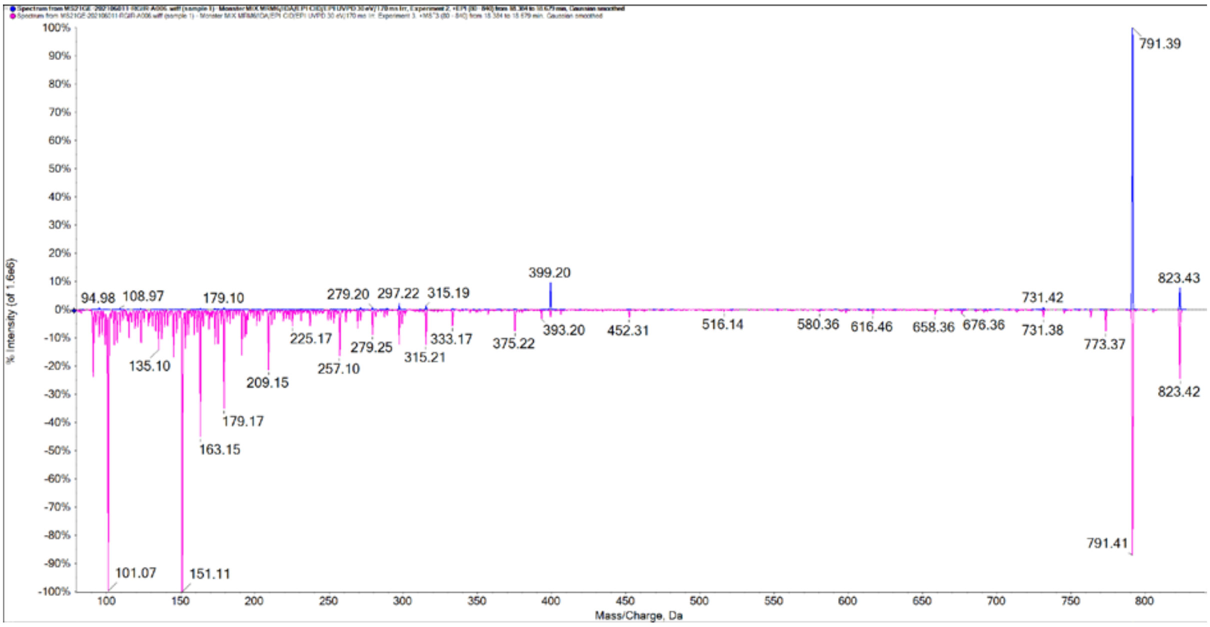

Figure S77. Rifampicin CID (upper) and UVPD (lower) fragmentation spectra zoom from 80 to 840 *m/z*.

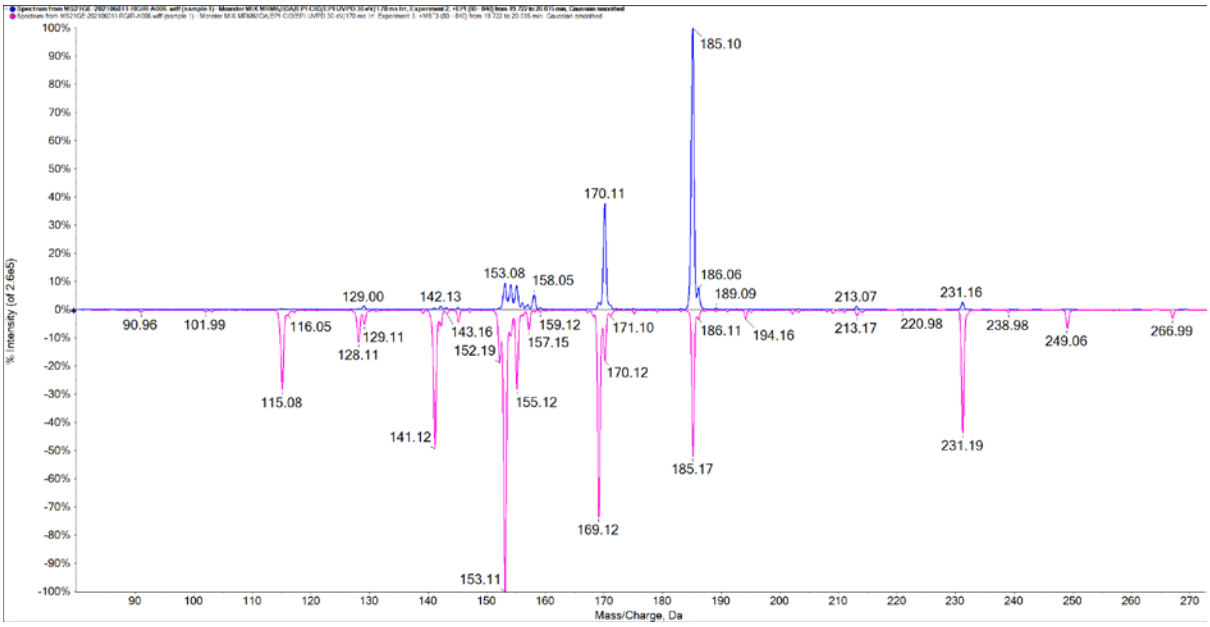

Figure S78. Naproxen CID (upper) and UVPD (lower) fragmentation spectra zoom from 80 to 270 *m/z*.

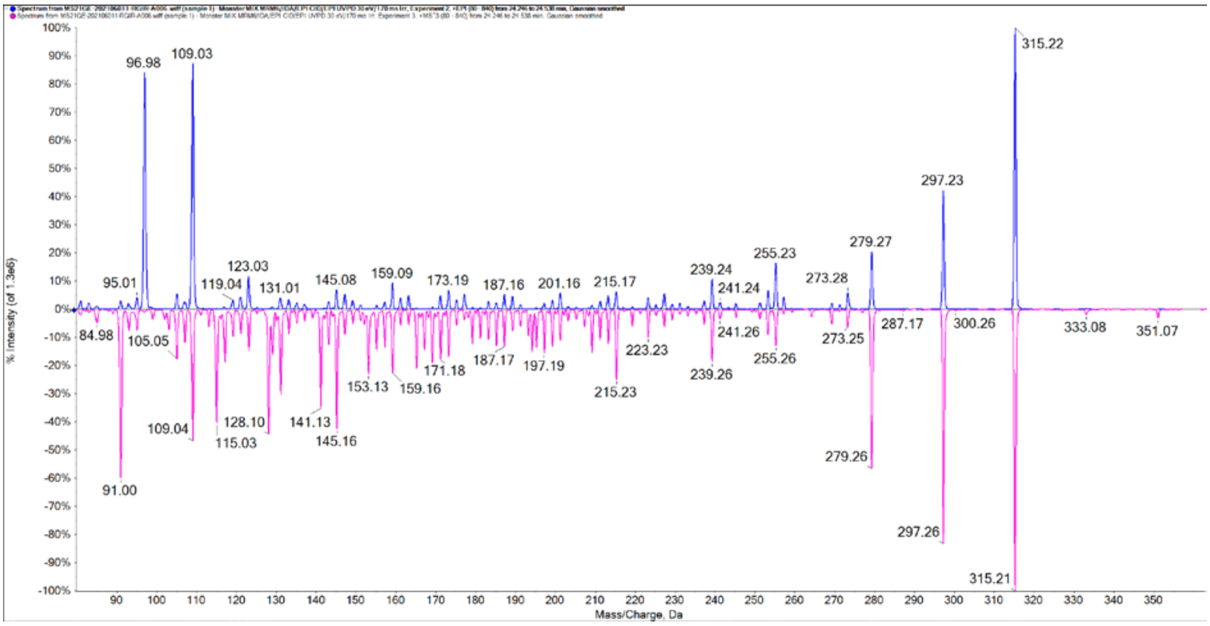

Figure S79. Progesterone CID (upper) and UVPD (lower) fragmentation spectra zoom from 80 to 360 *m/z*.

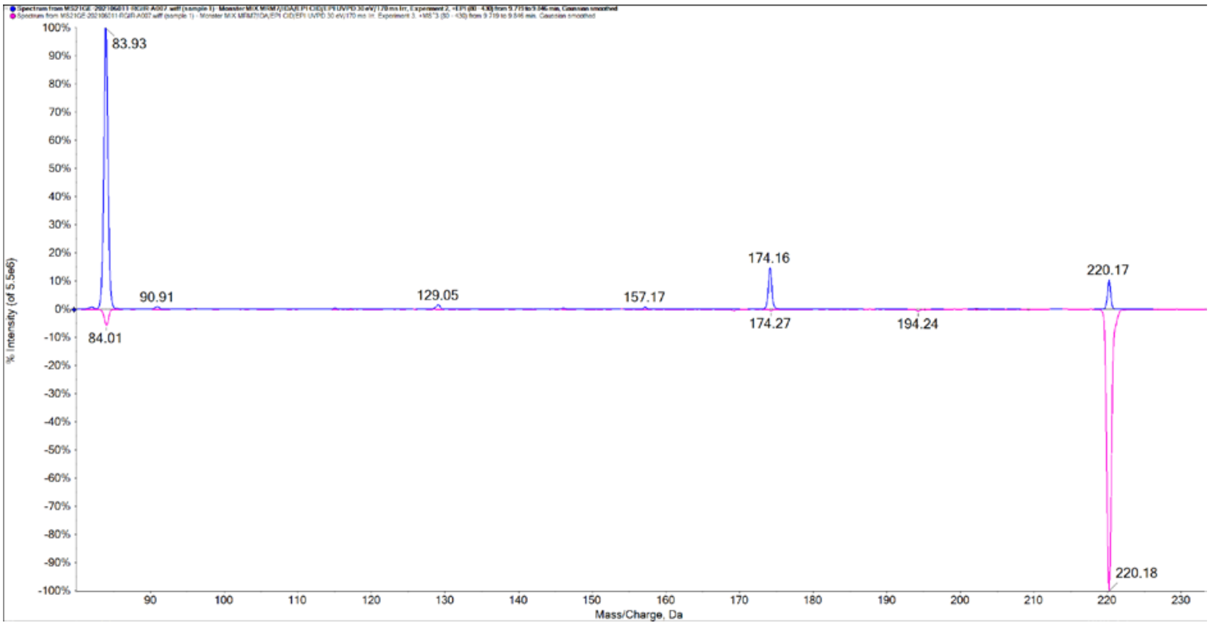

Figure S80. Ritanilic acid CID (upper) and UVPD (lower) fragmentation spectra zoom from 80 to 230 *m/z*.

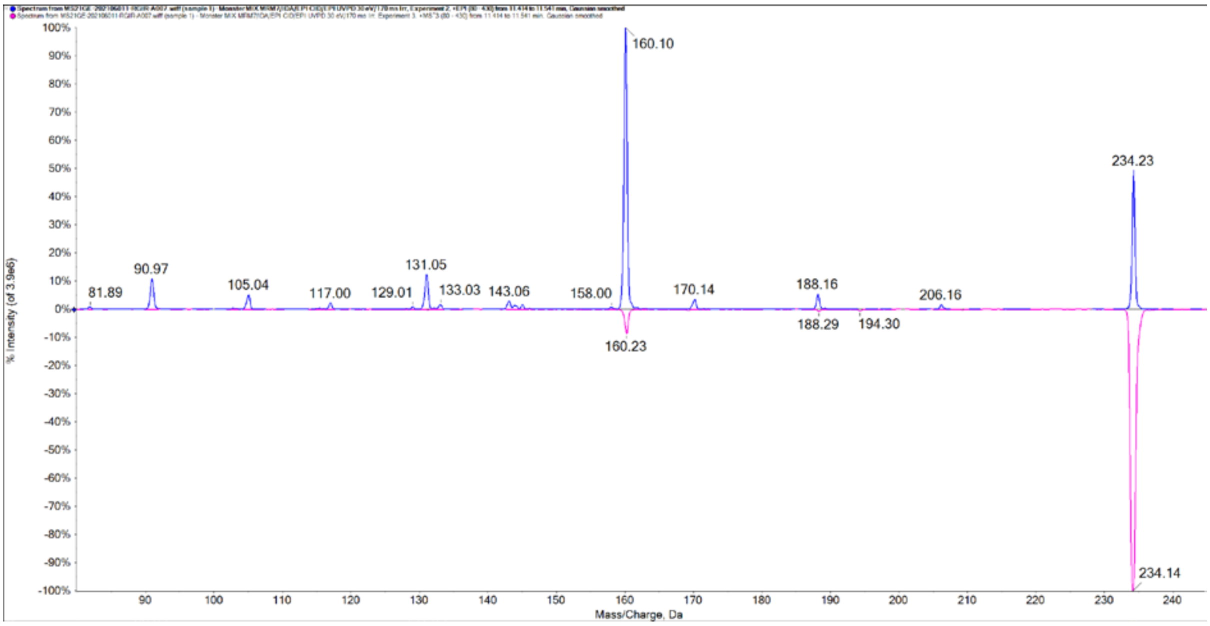

Figure S81. Normeperidine CID (upper) and UVPD (lower) fragmentation spectra zoom from 80 to 245 *m/z*.

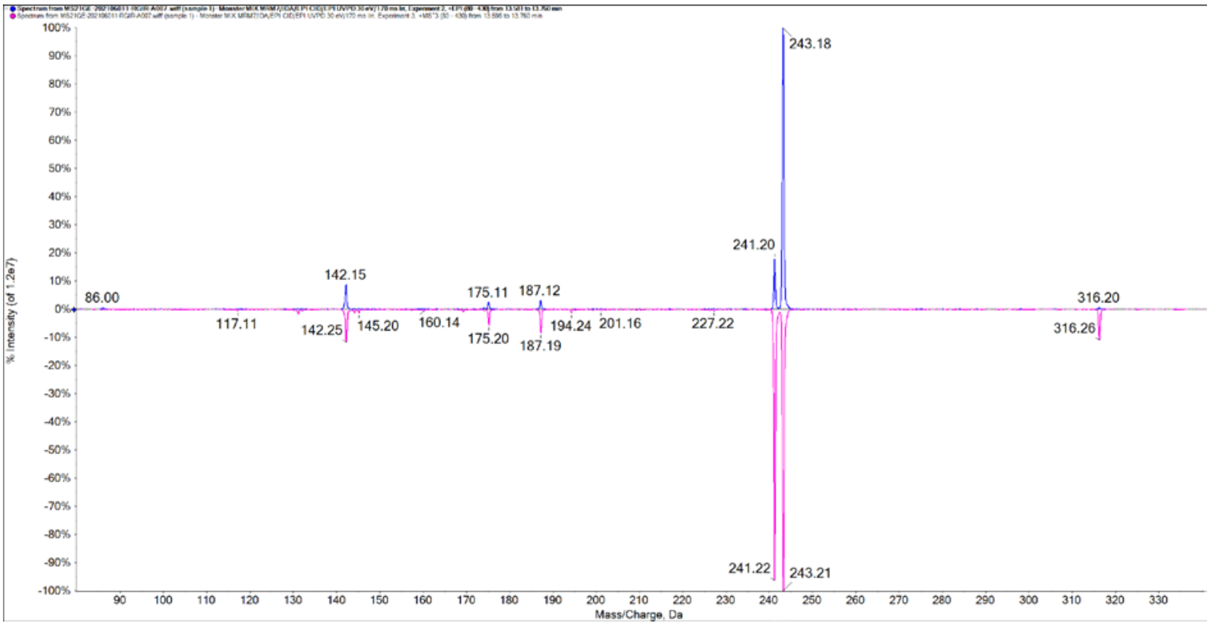

Figure S82. Pamaquine CID (upper) and UVPD (lower) fragmentation spectra zoom from 80 to 340 *m/z*.

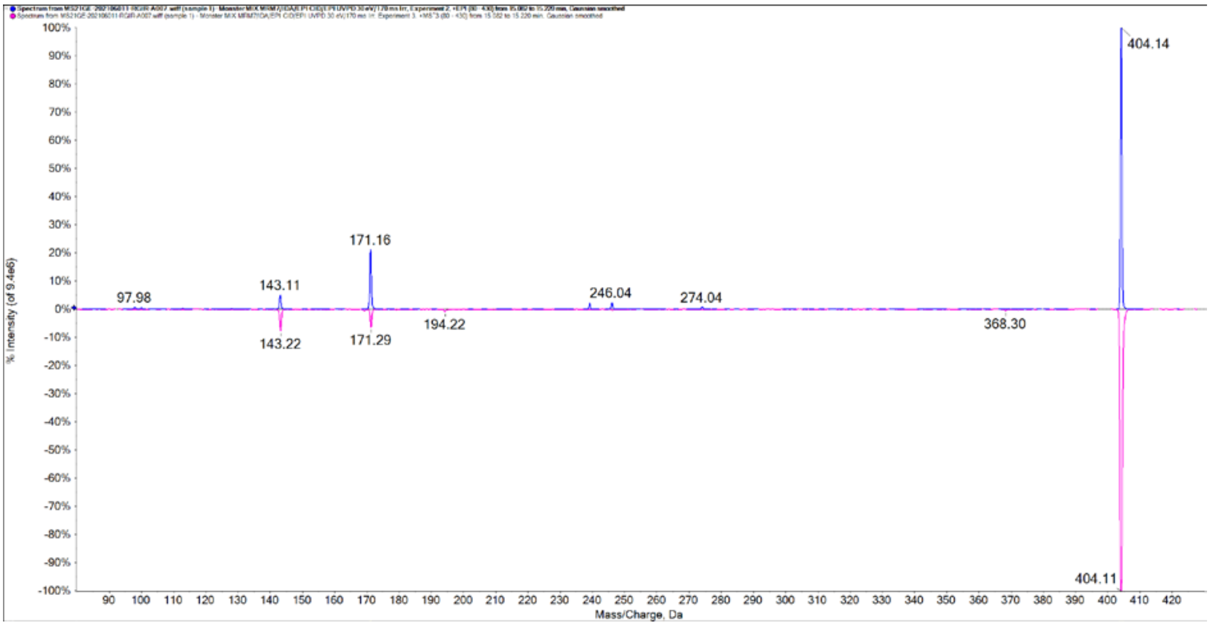

Figure S83. Perphenazine CID (upper) and UVPD (lower) fragmentation spectra zoom from 80 to 430 *m/z*.

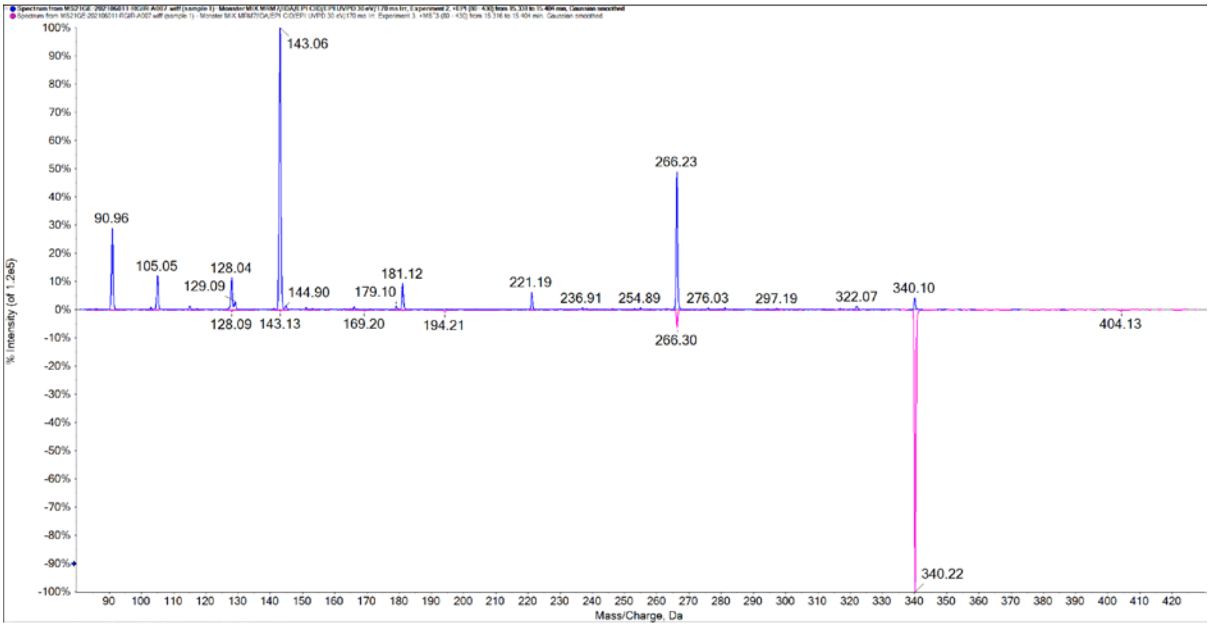

Figure S84. Propoxyphene CID (upper) and UVPD (lower) fragmentation spectra zoom from 80 to 430 *m/z*.

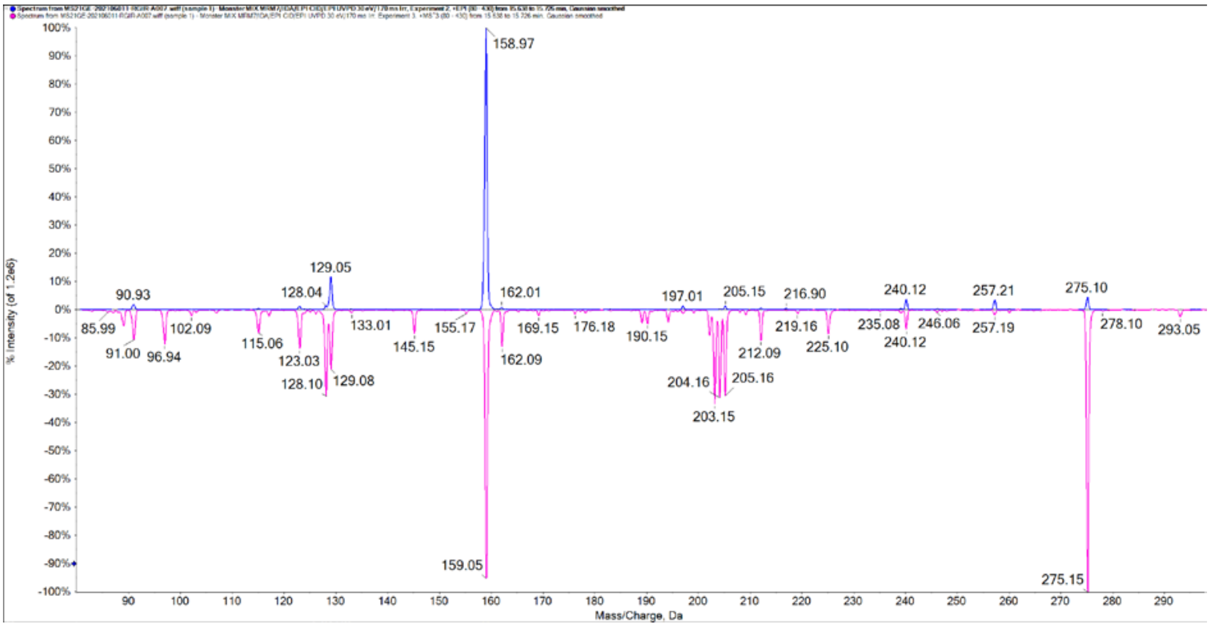

Figure S85. Norsertraline HCL CID (upper) and UVPD (lower) fragmentation spectra zoom from 80 to 300 *m/z*.

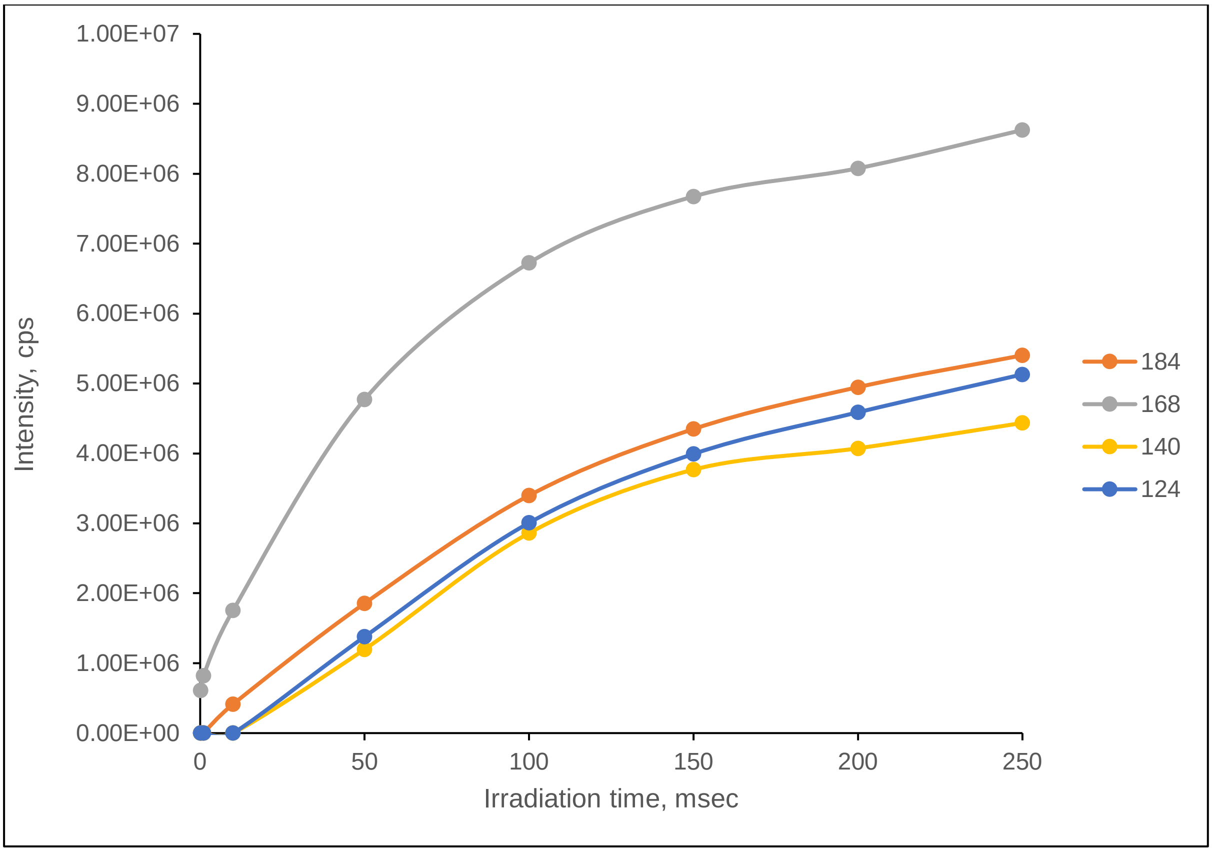

Figure S86. Benzoylecgonine fragments intensities according to the irradiation time (msec)

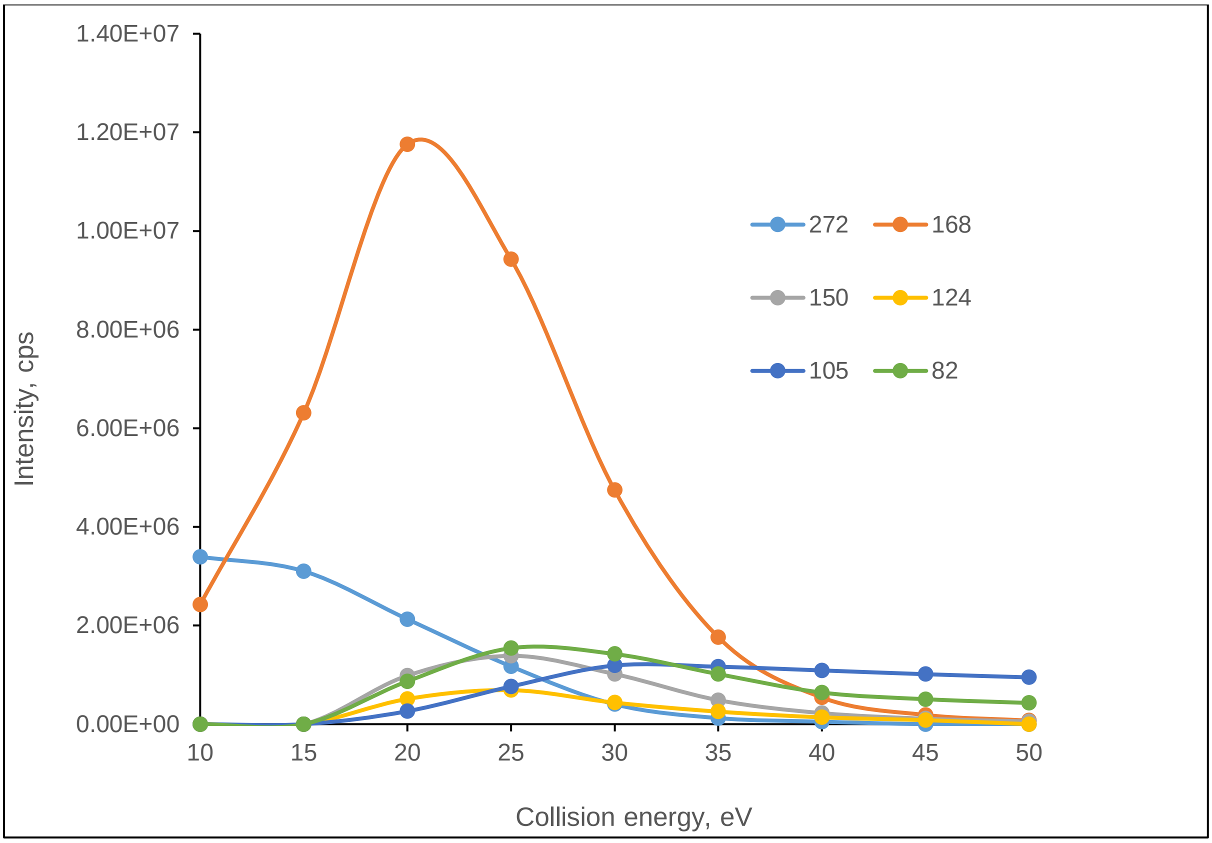

Figure S87. Benzoylecgonine fragments intensities according to the collision energy (eV)

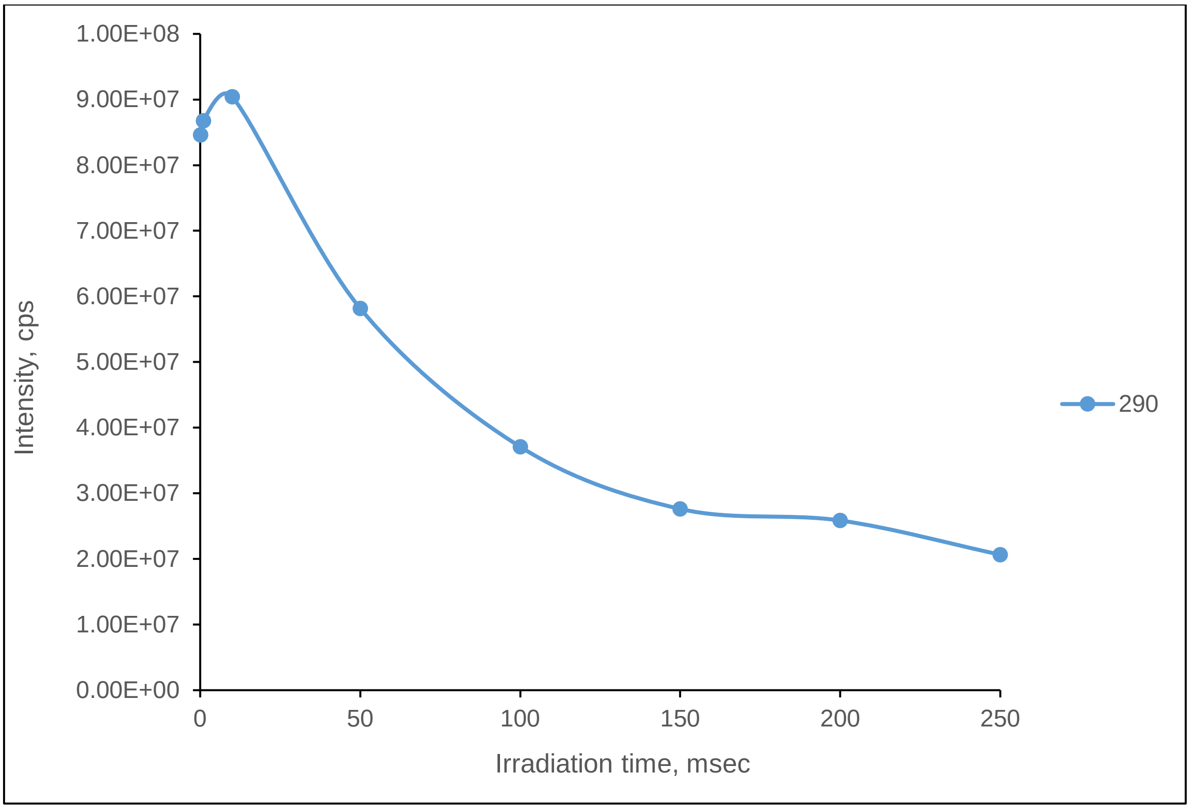

Figure S88. Residual benzoylecgonine precursor intensity according to the irradiation time (msec)

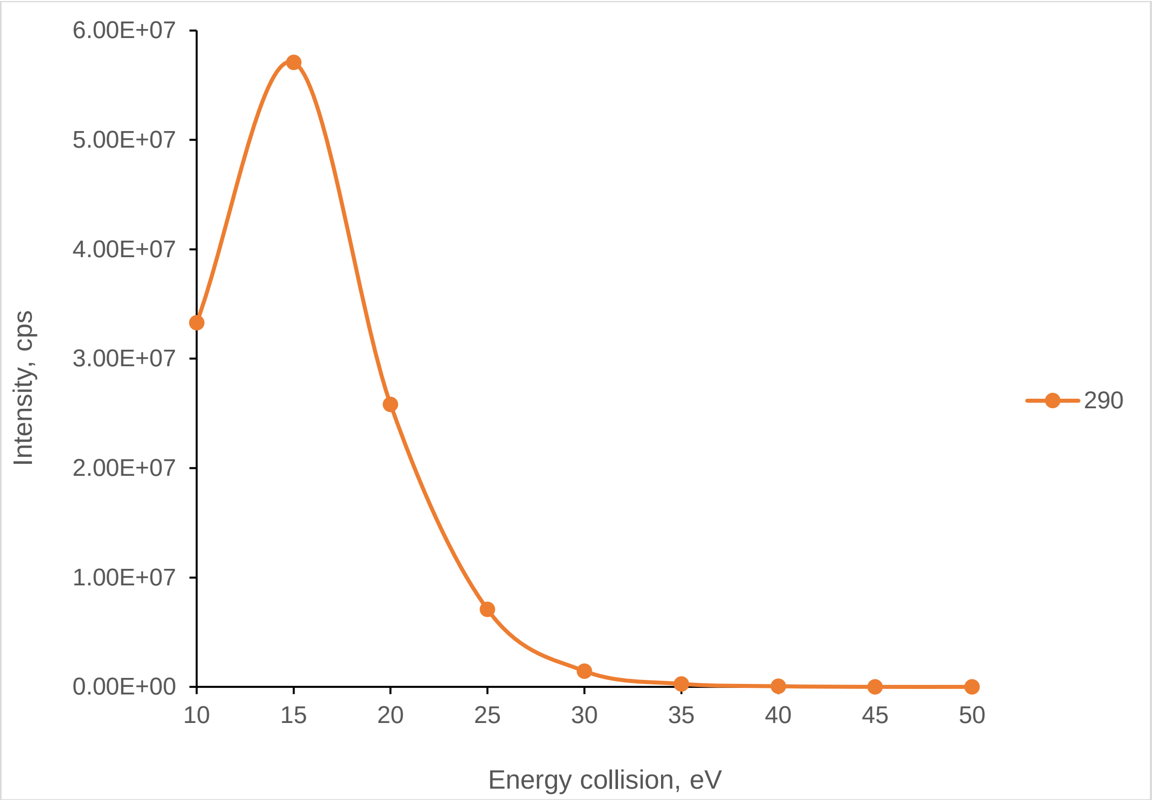

Figure S89. Residual benzoylecgonine precursor intensity according to the collision energy (eV)

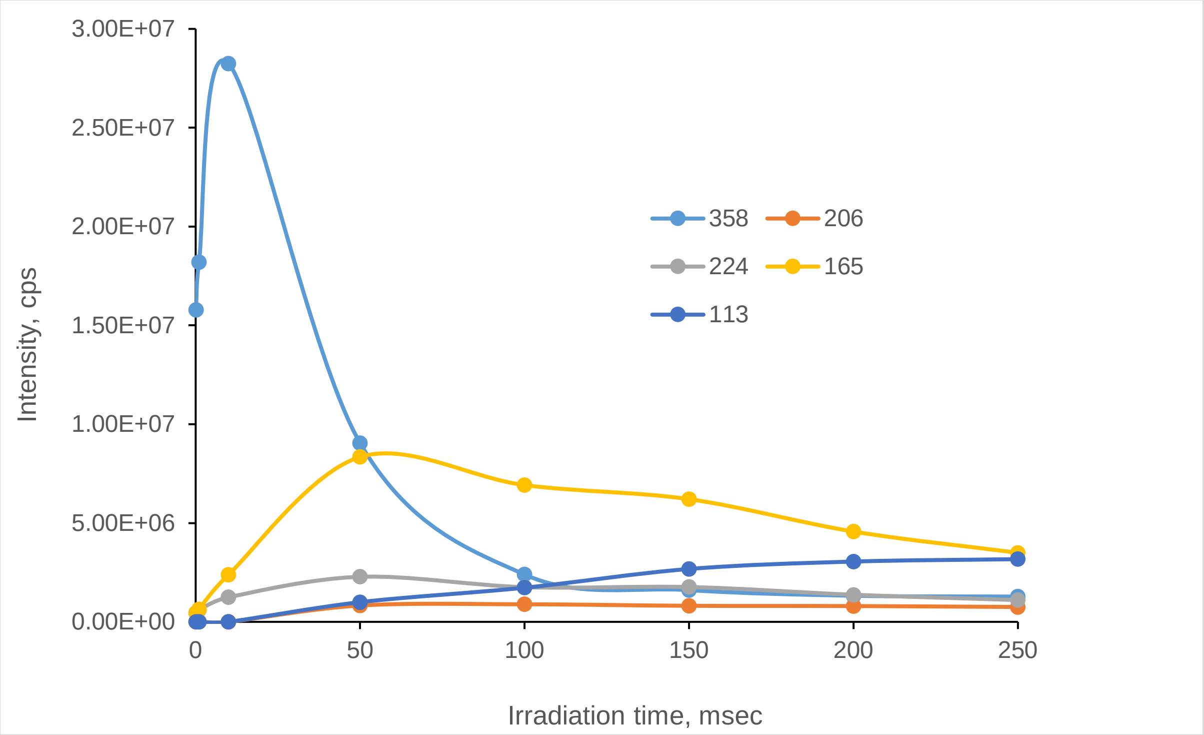

Figure S90. Haloperidol fragments intensities according to the irradiation time (msec)

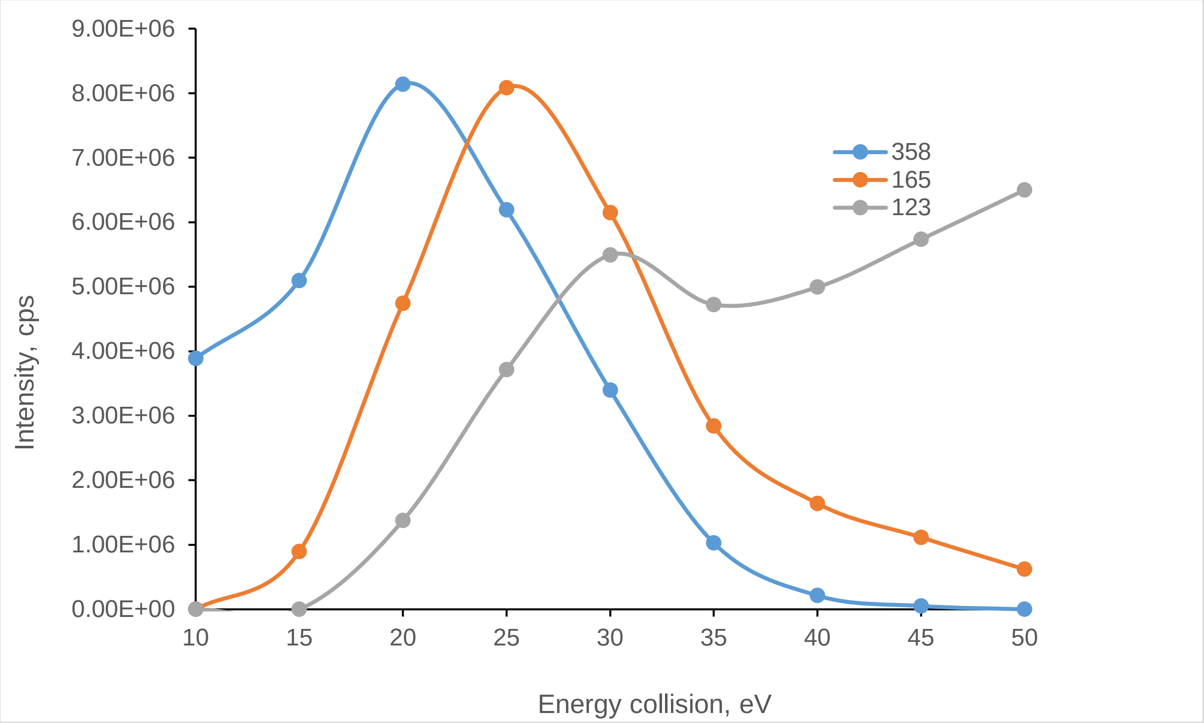

Figure S91. Haloperidol fragments intensities according to the collision energy (eV)

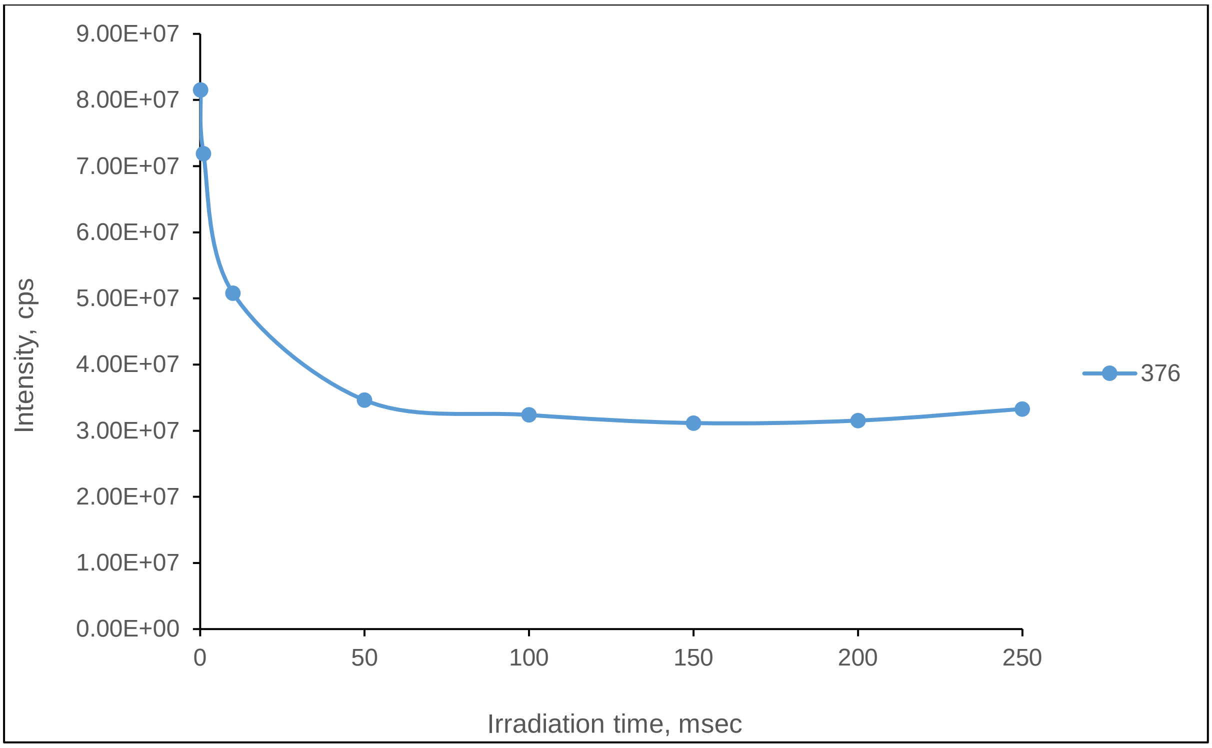

Figure S92. Residual haloperidol precursor intensity according to the irradiation time (msec)

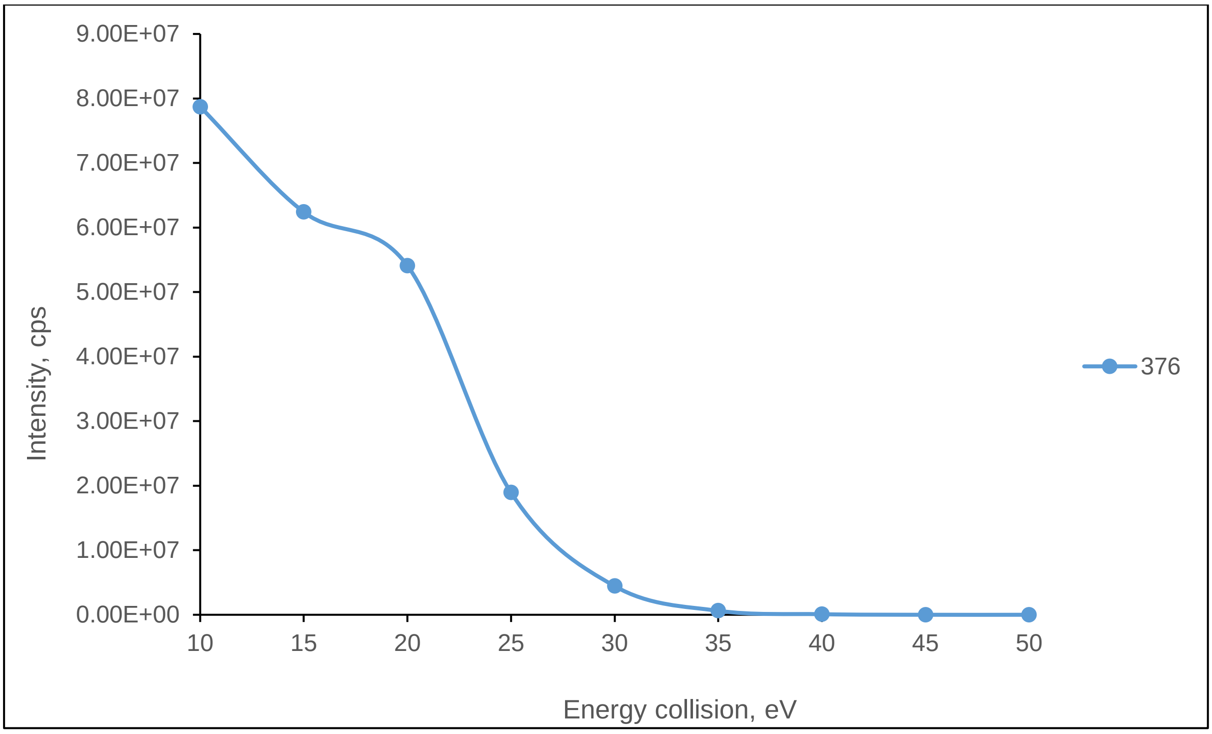

Figure S93. Residual haloperidol precursor intensity according to the collision energy (eV)

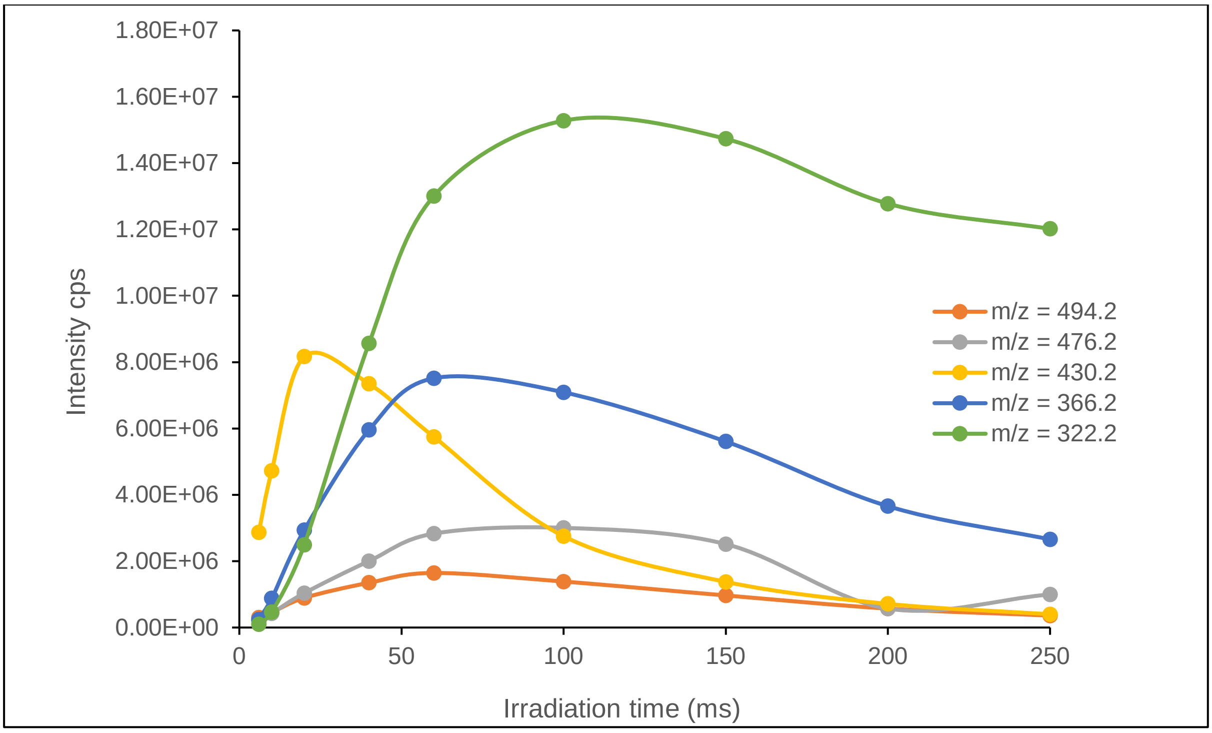

Figure S94. Desmethyl bosentan fragments intensities according to the irradiation time (msec)

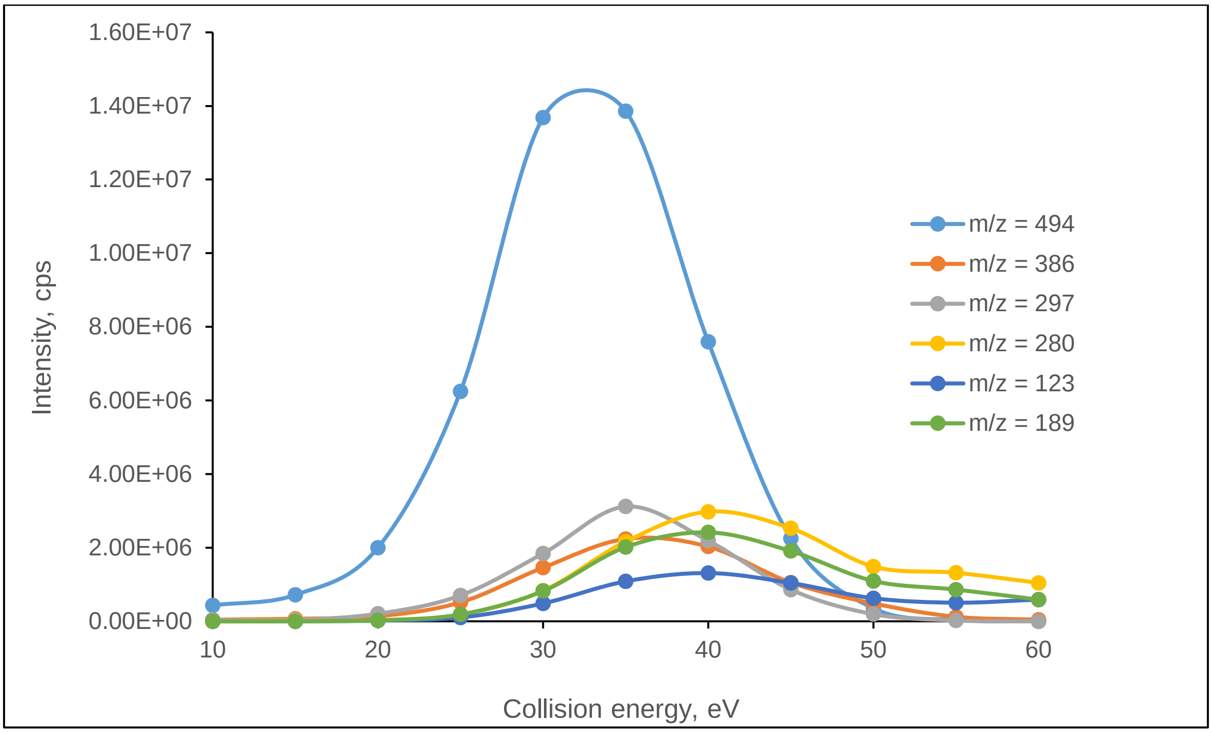

Figure S95. Desmethyl bosentan fragments intensities according to the collision energy (eV)

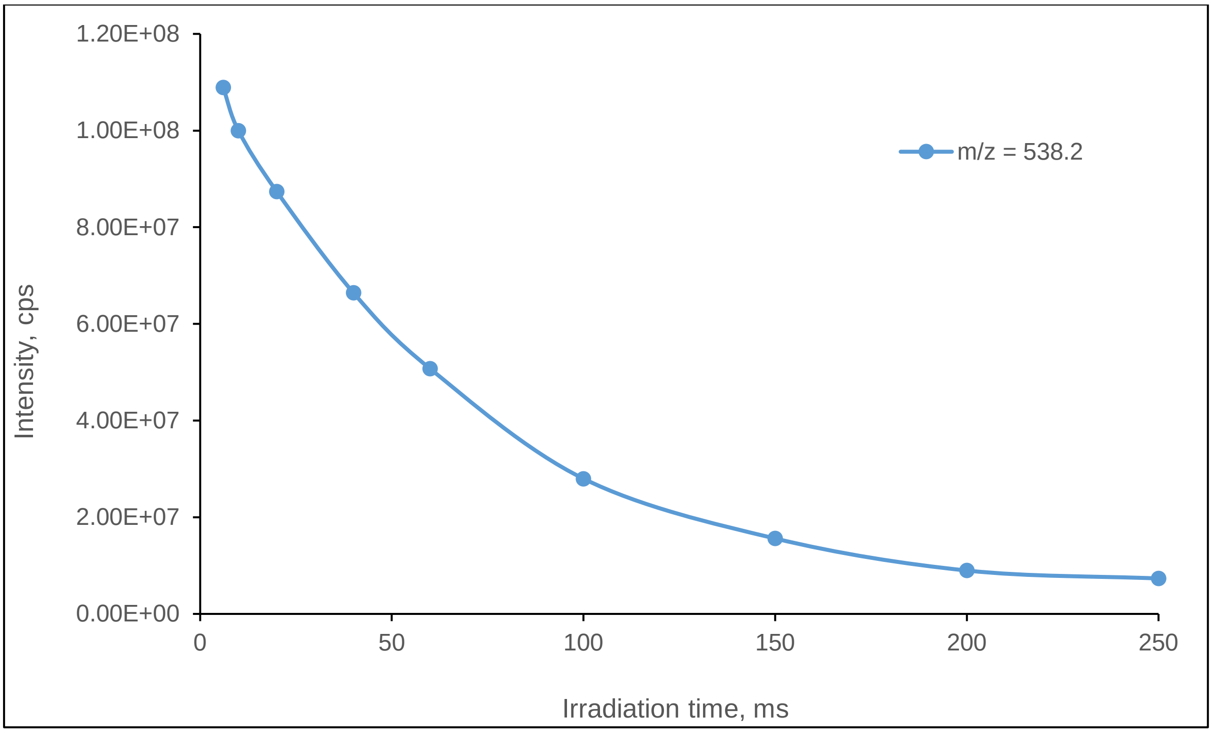

Figure S96. Residual desmethyl bosentan precursor intensity according to the irradiation time (msec)

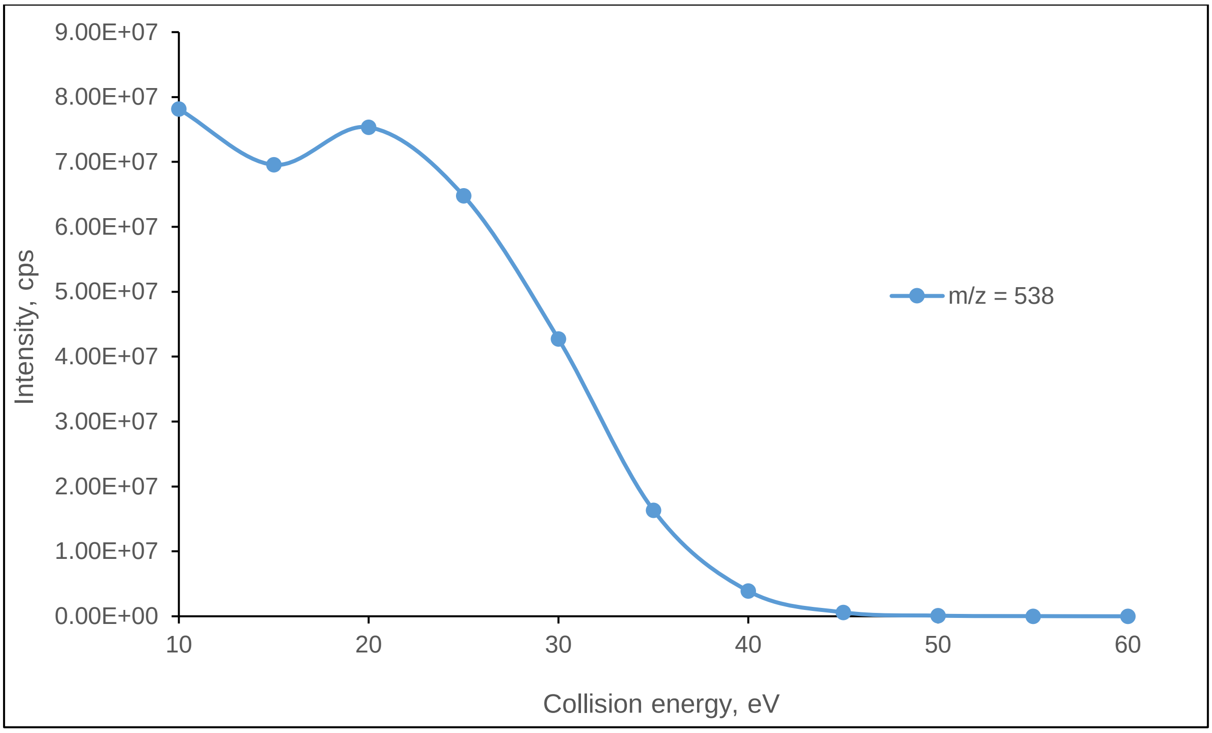

Figure S97. Residual desmethyl bosentan precursor intensity according to the collision energy (eV)

Table S2. Transitions MRM CID parameters for the bosentan and one of its metabolite quantification method apply to plasma sample.

Name	Q1	Q3	Dwell time (msec)	CE	CXP	EP	DP
**Bosentan**	552.2	202.2	10	46	10	10	86
**Desmethyl bosentan**	538.2	494.2	10	36	14	10	96
**Bosentan d4**	556.2	202.2	10	46	10	10	96
**Desmethyl bosentan d4**	542.2	494.2	10	36	14	10	86
